# The effectiveness of aerobic exercise on pain and disability in individuals with neck pain: A systematic review and meta‐analysis

**DOI:** 10.1113/EP091884

**Published:** 2024-11-19

**Authors:** Ana lzabela Sobral de Oliveira‐Souza, Marie Kempe, Sofia Grimmelsmann, Luiz Felipe Tavares, Ester Moreira De Castro‐Carletti, Angela Viegas Andrade, Liz Dennett, Harry Von Piekartz, Jorge Fuentes Contreras, Susan Armijo‐Olivo

**Affiliations:** ^1^ Faculty of Economics and Social Sciences University of Applied Sciences Osnabrück Osnabrück Germany; ^2^ Postgraduate program in Physical Therapy Federal University of São Carlos (UFSCar) São Carlos São Paulo Brazil; ^3^ Master's in Science of Rehabilitation Federal University of Minas Gerais Belo Horizonte Minas Gerais Brazil; ^4^ Geoffrey and Robyn Sperber Health Sciences Library Edmonton Alberta Canada; ^5^ Faculty of Rehabilitation Medicine University of Alberta Edmonton Alberta Canada; ^6^ Faculty of Health Sciences, Department of Physical Therapy, Clinical Research Lab Catholic University of Maule Talca Chile; ^7^ Faculties of Rehabilitation Medicine and Medicine and Dentistry, Department of Physical Therapy University of Alberta Edmonton Alberta Canada

**Keywords:** aerobic exercise, disability evaluation, neck pain, pain, physical therapy, systematic review, randomized controlled trials

## Abstract

The present review aimed to investigate the effectiveness of aerobic exercise (AE) compared to other interventions in decreasing pain intensity and reducing disability in individuals with neck pain. A systematic review (SR) of randomized controlled trials was conducted. This SR was registered in PROSPERO (CRD42021231231). Searches were conducted in five electronic databases (MEDLINE, Embase, CINAHL, Cochrane and SCOPUS). Studies were selected if they included adults over 18 years old with neck pain. The primary outcomes were pain intensity and physical function. A meta‐analysis was conducted when applicable. Cochrane RoB Tool‐2 was used to determine the risk of bias of included studies, and the certainty of the evidence was determined using the GRADE approach. Out of 4669 initial records screened, six studies published in 12 articles were included. AE was not statistically different compared to no‐treatment or other interventions (e.g., localized exercise or acupuncture) on pain intensity measured with a visual analogue scale (VAS) (mean difference (MD) [95%CI]: 5.16 mm [−6.38, 16.70]). Contrarily, strengthening exercise was better than AE for pain intensity (MD [95%CI]: −11.34 mm [−21.6, −1.09]) after treatment. However, when AE was combined with other therapy (strengthening exercises or acupuncture), the combined therapy was better than isolated treatments (MD [95%CI]: 7.71 mm [1.07, 14.35]). A high heterogeneity was observed between protocols, comparisons, and results (magnitudes and directions). In conclusion, AE had positive results only when combined with other therapies to reduce pain intensity and disability in patients with neck pain. However, the evidence is limited, low‐quality, and heterogeneous.

## Abstract (highlights)

- **What is the central question of this study?**
  What is the effectiveness of aerobic exercise (AE) in decreasing pain intensity and reducing disability in individuals with neck pain?
- **What is the main finding and its importance?**
  Aerobic exercise (AE) was shown to be equally effective as other active treatment modalities in reducing neck pain, although the overall certainty of the evidence was rated as low. When comparing before and after treatment, AE can also improve disability, functionality, and emotional status. Combining AE with other treatments seems to be a better alternative for reducing pain intensity than aerobic exercise alone. Potential advantages of AE include ease of performance, versatility, cost benefit, and ease of application in groups.

## 1 INTRODUCTION

Neck pain (NP) is one of the most common musculoskeletal (MSK) disorders, along with back pain, joint pain, and headaches (Breivik et al., 2013). The prevalence of NP has increased over the years; in 2020, an estimated 203 million people reported NP worldwide, an increase of 77.3% compared to 1990. Also, according to the Global Burden of Disease 2019, NP represents the 11th leading cause of years lost to disability (YLDs) (Vos et al., 2020). In addition, the total number of YLDs increased by approximately 76.2% in the last 30 years (Wu et al., 2024). Up to 70% of the general population experience NP at some point in their lives, and, on average, every person suffers from severe NP at least once (Palacios‐Cena et al., 2015). The prevalence of NP is higher in females than males, similar to the YLDs rates (Wu et al., 2024), peaking between the ages of 45 and 74. Moreover, a very recent global burden of disease study was developed and highlighted that the prevalence estimates for NP are about to increase by around 32.5% by 2050 (Wu et al., 2024). Consequently, a negative effect is observed related to work productivity (Wu et al., 2024).

Because NP has a high socio‐economic impact on society, providing conservative and low‐cost solutions for management can positively impact work productivity and reduce the burden on individuals' lives, the health care system, and the economy. Non‐pharmacological treatments such as self‐management advice, psychosocial intervention, and exercise therapy have been reported to help manage NP, with moderate to strong effect sizes (Babatunde et al., 2017; Polaski et al., 2019). Pharmacological interventions and complementary therapies have also demonstrated positive effects in the short‐term (Eftekharsadat et al., 2018; Machado et al., 2017). However, medications are not ideal for long‐term use due to their side effects (Babatunde et al., 2017). Therefore, non‐pharmacological treatment strategies are preferred for managing this condition. Exercise is a beneficial strategy in this context due to its advantages and minimal adverse effects (Geneen et al., 2017). Common types of exercises to treat MSK pain include strength training, motor control, endurance, aquatics, aerobic exercise (AE), and Thai Chi, among others (Polaski et al., 2019). In the last few decades, AE has emerged as a promising treatment for managing MSK disorders (De la Corte‐Rodriguez et al., 2024). For example, studies have highlighted that AE could improve different outcomes such as pain, quality of life, and function in individuals with varying types of MSK pain, including NP (Wassinger et al., 2020; Wewege et al., 2018).

AE is a collective term that describes training characterized by repetitive, structured physical activity that requires the body's metabolic system to use oxygen and produce energy (Millstein, 2013). AE is associated with pain reduction in individuals with different types of MSK pain and is often related to exercise‐induced hypoalgesia (EIH), which is characterized by a reduced sensitivity to noxious stimuli and pain perception due to exercise (Nicolini et al., 2021; Wassinger et al., 2020; Wewege et al., 2018). Despite the worldwide use of exercise to manage MSK disorders, its effect on pain modulation is not yet fully understood (Vaegter & Jones, 2020). However, the impact of exercise could be attributed to different pain modulation mechanisms, such as exercise induced hypoalgesia (EIH), reduced temporal summation of pain, and altered pain perception (Fredin & Lorås, 2017). Also, it is important to highlight that AE may potentially benefit the cardiovascular and metabolic systems (Gasibat et al., 2019), cognitive and psychological health (Kregel et al., 2015), and sleep quality (Abd El‐Kader & Al‐Jiffri, 2019). Therefore, AE could address chronic pain's biological and psychological dimensions.

Several systematic reviews (de Zoete, Armfield, et al., 2020; de Zoete, Brown, et al., 2020; Dinler et al., 2009; García‐Correa et al., 2021; Gross et al., 2016; Irby et al., 2016; Nijs et al., 2015) report positive results of AE for different types of MSK pain such as low back pain, osteoarthritis, and fibromyalgia (Wewege et al., 2018) with moderate (Rooks et al., 2007) and large (Brosseau et al., 2012) effect sizes for pain relief. Other systematic reviews have also confirmed the effectiveness of general exercises, such as strength, motor control, and endurance exercises for individuals with NP (Binder, 2008; Gross et al., 2016; Hurwitz et al., 2009; Polaski et al., 2019; Ylinen, 2007); nonetheless, most of them did not include AE as a treatment modality. One systematic review focused on AE in patients with NP, with important limitations (Paraskevopoulos et al., 2023). For example, searches were conducted in only two databases and limited to English and Greek language, no statistical analysis of data was performed, and some of the published trials were not included, consequently missing an in‐depth analysis about the effectiveness of the AE on NP management. Also, the inclusion of multimodal exercises in the analysis may have biased AE results (Paraskevopoulos et al., 2023).

One systematic review investigated the FITT components (i.e., frequency, intensity, time, and type) for different exercises in chronic NP (CNP) (O'Riordan et al., 2014). Although this review searched several databases, only articles written in English were included. In addition, the search was conducted up to 2012. Although the authors did not analyse the isolated effects of AEs, they concluded that AE components enhance the benefits of strengthening exercises since the combined AE and strengthening exercise presented better results than other groups (e.g., no treatment, waiting list, or placebo treatment). Another Cochrane systematic review investigated different forms of exercise for acute, subacute, and chronic NP (Gross et al., 2015). Although a comprehensive search strategy with no language restriction was conducted, the last search strategy was conducted up to 2014, and only three of the 27 included trials investigated the effectiveness of AE for any form of NP. However, only one of these three studies looked at the isolated effects of AE compared to a control group (Andersen, Kjaer, et al., 2008).

Although NP is highly prevalent and has an important impact on work productivity and life activities, the most effective intervention (s) for preventing or managing NP symptoms remains unclear. Thus, studies should concentrate on finding better causal explanations for NP and identifying more effective interventions for prevention and management to mitigate the burden of NP (Wu et al., 2024). Furthermore, no previous reviews have comprehensively focused on the effectiveness of AE on individuals with NP. In addition, research on this topic has emerged in recent years and requires an update. Thus, the aims of this systematic review were: (1) to compile and synthesize the evidence from randomized controlled trials (RCTs) and clinical trials regarding the effectiveness of AE in reducing pain and disability of individuals with NP when compared to other treatment strategies, such as localized exercises, medication, acupuncture, physical agents, and manual therapy; and (2) to explore which characteristics of AE (e.g., type, frequency, duration, and number of sessions) are the best to alleviate pain and disability and improve function in individuals with NP.

## 2 METHODS

This systematic review protocol was previously registered in PROSPERO (CRD42021231231). This study followed the updated PRISMA guidelines.3.5 (Page et al., 2021).

### 2.1 Study selection

This review was part of a large project looking at AE for several MSK conditions. The research team came up with an extensive list of potential search terms, and an experienced health sciences librarian conducted the searches in MEDLINE (Ovid MEDLINE(R) ALL), Embase (Ovid interface), CINAHL Plus with Full Text (EBSCOhost interface), Cochrane Trials database (CENTRAL) (Wiley Interface), and SCOPUS. The search combined an extensive list of subject headings and keywords for the concepts of NP (e.g., neck, cervical, cervicothoracic, cervicogenic, craniocervical, cervicodynia, and cervicalgia), and AE (e.g., physical conditioning, circuit‐based exercise, endurance training, high‐intensity interval training, running, jogging swimming, walking, stair climbing, physical endurance, physical exertion, physical fitness, and cardiorespiratory fitness). The last search was conducted from inception in all databases up to April 2023. The search was limited to RCTs and controlled clinical trials using a modified version of the RCT filter (Glanville et al., 2006). The complete search strategy is available in Supporting information Appendix 1. No language or date limits were applied. Conference abstracts were retrieved from the above databases where available and clinical trial registry records were searched through CENTRAL. Reference lists of included articles and reviews were checked for additional studies. In addition, the Web of Science database was used to search through all references of potentially suitable studies. When two or more articles presented data from the same population and the data came from the same study, we included all the articles in the review, but we considered them as one study.

### 2.2 Eligibility criteria

The inclusion and exclusion criteria are described based on the PICO (Population, Intervention, Comparison, and Outcomes) framework.

#### 2.2.1 Population

Studies with adults over 18 years old with non‐specific acute (less than two weeks), sub‐acute (from two to 12 weeks), or CNP (>12 weeks) as defined by the International Association for the Study of Pain (IASP) and the three degrees described by the NP task force (Supporting information, Appendix 2) were included. Studies including individuals with rheumatic, neurological, metabolic, vascular diseases, cancer, neuropathic pain, previous surgery in the cervical region, and pain that was not related to the MSK system were excluded. No limits were applied in terms of sex, ethnicity, and living country.

#### 2.2.2 Intervention

The intervention of interest was AE, defined as any repetitive, structured physical activity that requires the metabolic system to use oxygen and produce over an extended period of 20–60 min. Examples include cycling, running, hiking, swimming, walking, and others. All included studies should report how the exercise intensity was monitored (e.g., maximum heart rate (HR_max_), Borg rating of perceived exertion (RPE), metabolic equivalent of task (METs), among others). Studies were excluded if the effects of AE could not be isolated from other treatments.

#### 2.2.3 Comparators

AE was compared to any other conservative and non‐conservative therapy including but not limited to dry needling, electrotherapy, manual therapy, laser therapy, exercise therapy (e.g., strengthening training, resistance, stretching, among others), acupuncture, ultrasound, occlusal splint management, behavioral training, sham, medication therapy, surgery, placebo, or non‐treatment.

#### 2.2.4 Outcomes

The primary outcomes were pain intensity measured with any pain instrument and physical function measured with the Neck Disability Index (NDI). Secondary outcomes consider all the remaining domains from the Initiative on Methods, Measurement, and Pain Assessment in Clinical Trials (IMMPACT) recommendations, which include physical and emotional functioning, patients’ ratings of global improvement, adverse events, and participants’ disposition (i.e., adherence) (Turk et al., 2003).

#### 2.2.5 Designs

Only RCTs and controlled trials (CTs) were included since this review aimed to test AE's effectiveness.

#### 2.2.6 Time points

The following time points were considered: end of treatment (main time point of interest), short‐term (2–6 weeks after treatment), short‐to‐intermediate term (7–12 weeks), intermediate‐term (≥12 weeks to ≤12 months), and long‐term follow‐up (≥12 months).

### 2.3 Data screening

The search results were compiled in an EndNote database and then imported into Covidence (www.covidence.org), which was used for the screening process. Additionally, the PRISMA flowchart was used to organize and track the selection process (Higgins et al., 2019). Two independent reviewers performed article screening (abstract and full text), data extraction, and risk of bias assessments. In case of disagreement between the reviewers, a consensus meeting was held. A third reviewer was consulted if needed.

### 2.4 Data extraction

The research team developed and tested the standardized data extraction (DE) sheet used to extract data. Qualitative and quantitative data were extracted, including but not limited to study information, AE characteristics, outcomes, results, data analysis, conclusion, and limitations. Two independent reviewers performed the data extraction. In case of disagreement between the reviewers, a consensus meeting was held. All measures to compute effect sizes, such as mean, standard deviations, confidence intervals, and *P*‐values, were extracted. The authors were contacted to get unreported data in case of missing quantitative data (Öte Karaca et al., 2017; Saeterbakken et al., 2017).

### 2.5 Risk of bias assessment

The risk of bias (RoB) of the included studies was assessed by a compiled set of items (Olivo et al., 2008) and the updated version of the 2019 Cochrane Collaboration RoB‐2 Tool (RoB‐2) by two independent reviewers (Higgins et al., 2019). The RoB‐2 considers bias arising from five different domains: randomization process, deviations from intended intervention, missing outcome data, measurement of the outcome, and selection of the reported results. Based on the answers to these domains, an algorithm determined the overall RoB in low risk, some concerns, or high risk of bias (Higgins et al., 2019).

### 2.6 Data synthesis

The data were synthesized based on the type of intervention and outcome. Evidence tables and illustrations were used to compare study details, summarize results and outcomes, perform analysis (e.g., thematic or content‐related), and when appropriate, present qualitative and quantitative data. Studies were also checked for homogeneity, and the results were pooled when possible. Mean difference (MD) or standardized mean difference (SMD) was used for continuous data. Estimates and 95% confidence interval (CI) are provided for all meta‐analyses. Ordinal data were analysed as continuous data. The SMD was classified based on the recommendation by Cohen, considering small (*d* < 0.20), medium (*d* < 0.50), and large (*d* < 0.80) effect sizes (Cohen, 2013). Additionally, the strength of the evidence against the null hypothesis (*P*‐value) in the meta‐analysis results was interpreted based on the criteria proposed by Sterne and Smith (2001). Heterogeneity across studies was tested using *I* ^2^ statistics, with *I* ^2^ values of 25%, 50% and 75% representing low, medium, and high degrees of heterogeneity, respectively (Deeks et al., 2019). Due to the small number of studies, it was impossible to explore heterogeneity and perform sensitivity analyses exhaustively. In addition, due to the limited literature on this topic, funnel plots to explore publication bias were not conducted. Statistical analyses were performed using the Software Review Manager (RevMan) version 5.4.

### 2.7 Certainty/quality of the evidence

The Grading of Recommendations Assessment, Development, and Evaluation (GRADE approach) was used to rate the overall quality/certainty of the evidence, as recommended by Guyatt et al. (2011). The evidence can be classified as high quality (it is very unlikely that further research will change our confidence in the impact assessment), moderate quality (further research is likely to have an important impact on our confidence and estimated effects), low quality (further research is likely to have an important influence our confidence and estimated effects) and very low quality (any estimate of the effect is very uncertain). Domains that may reduce evidence are (a) study limitations, (b) inconsistency of results, (c) indirectness of evidence, (d) imprecision, and (e) reporting bias. Two independent reviewers implemented GRADE; any discrepancies in overall quality ratings were resolved by a consensus among the reviewers, and if necessary, the main investigator made the final decision.

## 3 RESULTS

### 3.1 General overview

**FIGURE 1:**
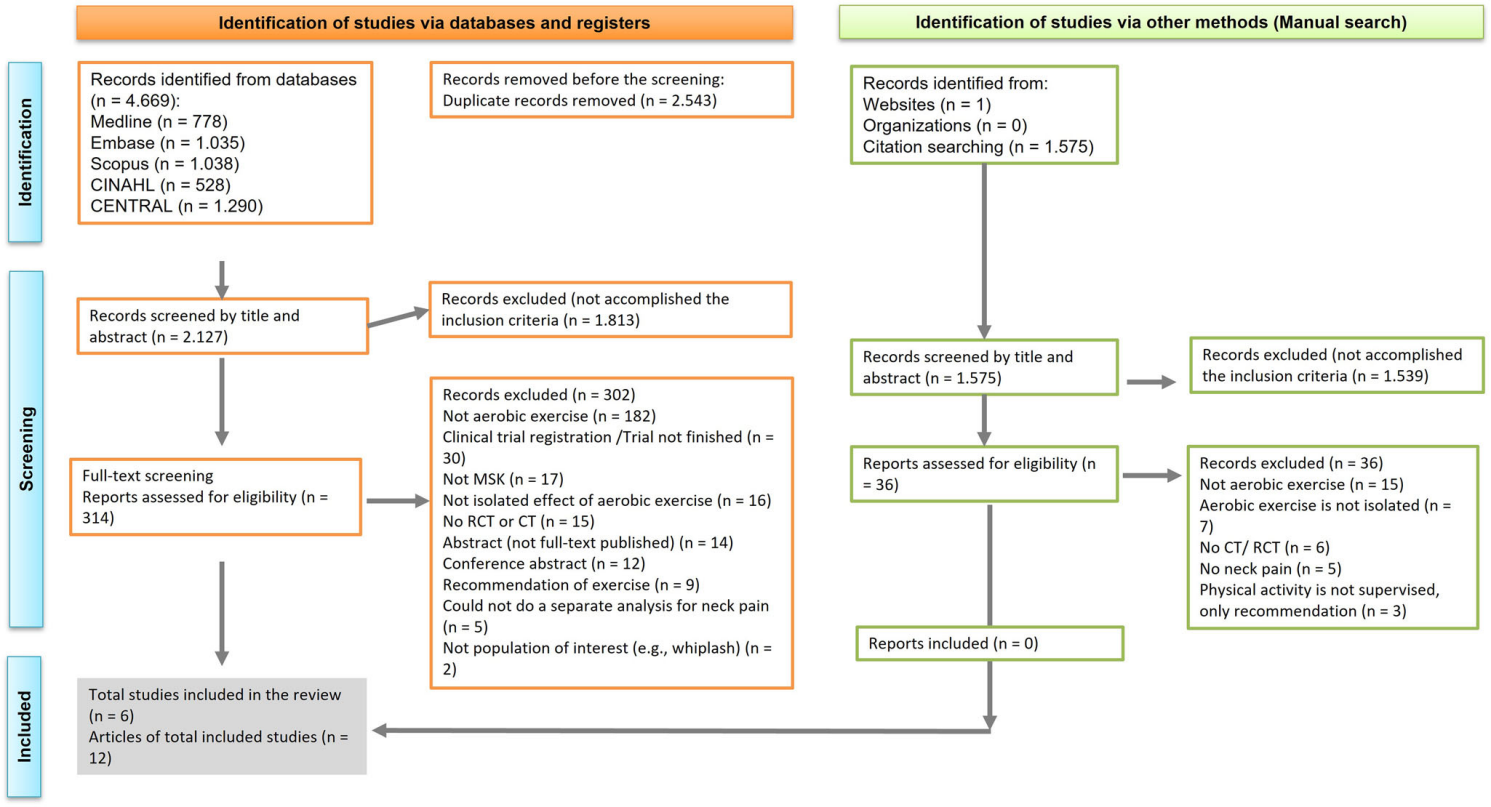
PRISMA flowchart.

After a thorough literature search in all databases, 4669 studies were found, and 2543 were excluded as duplicates. The remaining 2127 studies were screened based on their titles and abstracts, and 1813 were excluded. The remaining 314 studies were reviewed in full text, and 302 were excluded. Six studies reported on 12 articles (two studies were reported in more than one article, but data came from the same population and they were considered as one study) (Andersen et al., 2009; Andersen, Andersen, et al., 2008; Andersen, Jørgensen, et al., 2008; Daher et al., 2022, 2020, 2021; Eftekharsadat et al., 2018; Kocur et al., 2017; Korshøj et al., 2018; Mackey et al., 2011; Saeterbakken et al., 2017; Sogaard et al., 2012) were included in this systematic review. Figure 1 details the study selection process and reasons for exclusion (PRISMA flowchart). The list of excluded studies and reasons is provided in Appendix 3.

### 3.2 Description of included studies

Most studies were published in the last five years (*n* = 5, 83.3%) and in English (*n* = 6, 100%). Denmark had the most publications (*n* = 2, 33.3%). Half of the studies included both sexes (*n* = 3, 50%) and half just females (*n* = 3, 50%). Only four studies (66.7%) reported clinical trial registration. Five types of AE were used in the included studies, such as indoor cycling with ergometer (*n* = 4, 66.7%), outdoor walking (*n* = 3, 50%), outdoor cycling (*n* = 1, 16.7%), running treadmill (*n* = 1, 16.7%), and circuit training (*n* = 1, 16.7%). Tables 1 and 2 provide details of the included studies.

**TABLE 1:** General characteristics of included studies (*n* = 6).

STUDY CHARACTERISTIC	n (%)	POPULATION CHARACTERISTIC	n (%)
COUNTRY		RECRUITMENT PLACE	
Denmark	2 (33.3)	Workplace: Cleaning companies	1 (16.7)
Israel	1 (16.7)	Workplace: Industrial production or office	3 (50)
Norway	1 (16.7)	**Outpatient clinics**	2 (33.3)
Poland	1 (16.7)	**SEX**	
Iran	1 (16.7)	Mixed	3 (50)
LANGUAGE		Female	3 (50)
English	6 (100)	**CONDITION**	
YEAR OF PUBLICATION		Isolated Neck pain	1 (16.7)
Between 2008 and 2012	1 (16.7)	Trapezius myalgia	2 (33.3)
2017	2 (33.3)	Neck pain + other MSK pain	3 (50)
2018	2 (33.3)	**DIAGNOSIS TOOL**	
2020	1 (16.7)	Clinical diagnosis/ examination	4 (66.7)
STUDY DESIGN: RCT TYPE		Self‐reported pain	1 (16.7)
Parallel	5 (83.3)	Survey	1 (16.7)
Cluster	1 (16.7)	DURATION OF DIAGNOSIS*	
ETHICAL APPROVAL		Not reported	3 (50)
Yes	5 (83.3)	Reported to be chronic (>3 Months)	3 (50)
Not reported	1 (16.7)	Reported mean for all groups (= 1.3 years)	1 (16.7)
TRIAL REGISTERED		Reported mean for all groups (=0.6 years)	1 (16.7)
Yes	4 (66.7)	TYPES OF AEROBIC EXERCISE*	
Not reported	2 (33.3)	Cycling (ergometer)	4 (66.7)
FUNDING		Cycling (outdoor)	1 (16.7)
Government	2 (33.3)	Running (treadmill)	1 (16.7)
Academic	1 (16.7)	Walking (outdoor)	3 (50)
No funding	1 (16.7)	Circuit training	1 (16.7)
Not reported	2 (33.3)	**ALONE OR COMBINED**	
STUDY SETTING		Isolated aerobic exercise	4 (66.7)
Private Clinic	1 (16.7)	Combined (+ strength/stretching)	1 (16.7)
Outpatient Clinic	1 (16.7)	Combined (+ acupuncture/stretching)	1 (16.7)
Unclear	3 (50)	OTHER TYPES OF INTERVENTION*	
Not reported	1 (16.7)	Strength exercise	3 (50)
SAMPLING METHOD		Education	1 (16.7)
Convenience	6 (100)	Acupuncture	1 (16.7)
		Control	3 (50)
SAMPLE SIZE CALCULATION		**PAIN OUTCOMES**	
Yes	3	Self‐reported pain (VAS)	4 (66.7)
No	3	Nordic Questionnaire	1 (16.7)
		Pain sensitivity (Pain pressure threshold)	1 (16.7)

^*^Some of the studies are included in more than one category. Therefore, the numbers do not add up.

**TABLE 2:** Summary of randomized controlled trials included in this systematic review.

Study number	Study (year)	Participants	Intervention group	Comparison group(s)	Protocol of aerobic exercise	Outcome of interest	Results between groups (MD [95%CI])	Conclusion
1	Andersen, Andersen et al. (2008) Funding: this study was supported by the Danish Medical Research Council Grant 22‐03‐0264 and Danish Rheumatism Association Grant 233‐1149‐02.02.04 Setting: NA	***n* =** 42 **Age**: 44 (SD 8) years **Sex**: female **Diagnosis**: trapezius myalgia **Chronic pain**: yes	**Active treatment of interest (*n* = 18)** Specific strength training (SST) TTO: five dumbbell exercises for the shoulder/neck muscles Duration of intervention: 10 weeks Number of sessions: 30 Frequency: 3×/week Duration of the session: 20 min	**Active comparator (1) (*n* = 16)** General fitness training (AE) Duration of intervention: 10 weeks Number of sessions: 30 Frequency: 3×/weeks Duration of the session: 20 min **Reference group (2) (*n* = 14)** Advice to continue with normal activities (EDU) Duration of intervention: NA Number of sessions: NA Frequency: NA Duration of the session: NA	**Type of AE**: leg bicycling **Intensity**: moderate 50%–70% **Follow‐up**: no	**(1) Pain intensity** (VAS, 0–100 mm) (a) Prolonged effect: general pain (b) Acute effect: different time points before and after the training session **(2) Aerobic fitness** (Åstrand ${\dot{V}}_{O_{2}\max}$) **(3) Maximal voluntary isometric muscle strength (MVIS)** (handheld dynamometer)	**(1) Pain intensity (a)** AE vs. STR: MD: −18 [95%CI: −31.23, −4.77] mm AE vs. EDU: MD: 1 [95%CI: −17.81, 19.81] mm STR vs. EDU: MD: 19 [95%CI: 1.92, 36.07] mm **(2) and (3) Aerobic fitness and MVIS** No differences between groups	Strength exercise is better than AE and education therapy just to improve pain in patients with NP
	Andersen, Jørgensen et al. (2008) Funding: this study was supported by the Danish Medical Research Council Grant 22‐03‐0264 and Danish Rheumatism Association Grant 233‐1149‐02.02.04 Setting: NA	***n* =** 42 **Age**: 44 (SD 8) years **Sex**: female **Diagnosis**: trapezius myalgia **Chronic pain**: yes	**Active treatment of interest (*n* = 18)** Specific strength training (SST) TTO: five dumbbell exercises for the shoulder/neck muscles Duration of intervention: 10 weeks Number of sessions: 30 Frequency: 3×/week Duration of the session: 20 min	**Active comparator (1) (*n* = 16)** General fitness training (AE) Duration of intervention: 10 weeks Number of sessions: 30 Frequency: 3×/weeks Duration of the session: 20 min **Reference group (2) (*n* = 16)** Advice to continue with normal activities (EDU). Duration of intervention: NA Number of sessions: NA Frequency: NA Duration of the session: NA	**Type of AE**: leg bicycling **Intensity**: moderate 50–70% ${\dot{V}}_{O_{2}\max}$ **Follow‐up**: no	**(1) Pain intensity** (VAS, 0–100 mm) At rest, immediately before and immediately after the dynamometer test **(2) Shoulder abduction strength** (dynamometry; EMG) **(3) Reference contraction** (dynamometry; EMG) **(4) Muscle thickness** (ultrasound)	No separate values for AE group were provided for: **(1) Pain intensity** **(2) Shoulder abduction strength** **(3) Reference contraction** **(4) Muscle thickness**	No conclusion was possible since the authors did not provide any comparison groups results for the aerobic exercise group
	Sogaard et al. (2012) Funding: this study was supported by the Danish Medical Research Council Grant 22‐03‐0264 and Danish Rheumatism Association Grant 233‐1149‐02.02.04 Setting: unclear	***n* =** 42 **Age**: AE: 45.5 (SD 8) years SST: 44.6 (SD 8.5) years EDU: 42.5 (SD 11.1) years **Sex**: female **Diagnosis**: trapezius myalgia **Chronic pain**: yes	**Active treatment of interest (*n* = 16)** Specific strength training (SST) TTO: five dumbbell exercises for the shoulder/neck muscles Duration of intervention: 10 weeks Number of sessions: 30 Frequency: 3×/week Duration of the session: 20 min	**Active comparator (1) (*n* = 15)** General fitness training (AE) Duration of intervention: 10 weeks Number of sessions: 30 Frequency: 3×/weeks Duration of the session: 20 min **Reference group (2) (*n* = 8)** Advice to continue with normal activities (EDU) Duration of intervention: NA Number of sessions: NA Frequency: NA Duration of the session: NA	**Type of AE**: leg bicycling **Intensity**: moderate 50–70% ${\dot{V}}_{O_{2}\max}$ **Follow‐up**: no	**(1) Pain intensity** (VAS, 0–100 mm) (a) At rest, before pegboard test (b) Rate of pain development during pegboard test **(2) Change activation** (EMG, MMG) **(3) Tissue oxygenation** (NIRS) **(4) Blood pressure** (Finometer^TM^ blood pressure monitor)	**(1) Pain intensity (a)** AE vs. SST: MD: −7.6 [95%CI: −26.83, 11.63] mm; *P* = 0.44 AE vs. EDU: MD: −3.7 [95%CI: −25.94, 18.54] mm; *P* = 0.74 SST vs. EDU: MD: 3.9 [95%CI: −16.05, 23.85] mm; *P* = 0.71 **(2) Change activation** After the intervention, no changes were seen for any of the EMG or MMG values during the stress task. **(3) Tissue oxygenation** The STR presented better tissue oxygenation compared to the other groups after the treatment (*P* < 0.001). **(4) Blood pressure** No differences between groups.	No difference between groups was found in pain intensity after the treatment
	Andersen et al. (2009) Funding: this study was supported by the Danish Medical Research Council Grant 22‐03‐0264 and Danish Rheumatism Association Grant 233‐1149‐02.02.04 Setting: unclear	***n* =** 42 **Age**: 44 (SD 8) years **Sex**: female **Diagnosis**: trapezius myalgia **Chronic pain**: yes	**Active treatment of interest (*n* = 18)** Specific strength training (SST) TTO: five dumbbell exercises for the shoulder/neck muscles Duration of intervention: 10 weeks Number of sessions: 30 Frequency: 3×/week Duration of the session: 20 min	**Active comparator (1) (*n* = 16)** General fitness training (AE) Duration of intervention: 10 weeks Number of sessions: 30 Frequency: 3×/weeks Duration of the session: 20 min **Reference group (2) (*n* = 14)** Advice to continue with normal activities (EDU) Duration of intervention: NA Number of sessions: NA Frequency: NA Duration of the session: NA	**Type of AE**: leg bicycling **Intensity**: moderate 50%–70% ${\dot{V}}_{O_{2}\max}$ **Follow‐up**: no	**(1) Pain intensity** (VAS, 0–100 mm) (a) Prolonged effect: general pain **(2) Maximal muscle activation and torque** (dynamometry; EMG) **(3) Rapid muscle activation and torque** (dynamometry; EMG)	**(1) Pain intensity (a)** AE vs. SST: MD: −18.00 [95%CI: −31.23, −4.77]; *P* = 0.008 AE vs. EDU: MD: 1.00 [95%CI: −17.81, 19.81]; *P* = 0.92 STR vs. EDU: MD: 19 [95%CI: 1.92, 36.07]; *P* = 0.02 **(2) Maximal muscle activation and torque, rapid muscle activation and torque** No differences between groups	Strength exercise is better than AE and education therapy just to improve pain in patients with NP
	Mackey et al. (2011) Funding: this study was supported by the Danish Medical Research Council Grant 22‐03‐0264 and Danish Rheumatism Association Grant 233‐1149‐02.02.04 Setting: unclear	***n* =** 42 **Age**: 44 (SD 8) years **Sex**: female **Diagnosis**: trapezius myalgia **Chronic pain**: yes	**Active treatment of interest (*n* = 18)** Specific strength training (STR) TTO: five dumbbell exercises for the shoulder/neck muscles Duration of intervention: 10 weeks Number of sessions: 30 Frequency: 3×/week Duration of the session: 20 min	**Active comparator (1) (*n* = 16)** General fitness training (AE) Duration of intervention: 10 weeks Number of sessions: 30 Frequency: 3×/weeks Duration of the session: 20 min **Reference group (2) (*n* = 8)** Advice to continue with normal activities (EDU) Duration of intervention: NA Number of sessions: NA Frequency: NA Duration of the session: NA	**Type of AE**: leg bicycling **Intensity**: moderate 50–70% ${\dot{V}}_{O_{2}\max}$ **Follow‐up**: no	**(1) Satellite cells** **(2) Myonuclei** **(3) Macrophages** **(4) Cellular activity** **(5) Fibre remodelling**	No outcome measurement for interest.	No outcome measurement for interest
2	Daher et al. (2020) Funding: the National Insurance Institute of Israel, the Israel Physiotherapy Society and the University of Haifa Setting: outpatient clinic	***n* =** 139 **Age**: 54.6 (SD 10.5) years **Sex**: mixed **Diagnosis**: non‐specific NP + other MSK complaints **Chronicity**: yes	**Active treatment of interest (*n* = 69)** Strength training + aerobic exercise (STR + AE) TTO: the programme consisted of stretching and muscle performance exercises + AE Duration of intervention: 6 weeks Number of sessions: 24 Frequency: 2×/week supervised and 2× homebased = 4x/weeks Duration of the session: Week 1: 20 min; Week 2: 30 min; Weeks 3–6: 45 min	**Active comparator (*n* = 70)** Strength training alone (STR) TTO: the programme consisted of stretching and muscle performance exercises for neck muscles Duration of intervention: 6 weeks Number of sessions: 2×/week Frequency: 2×/week supervised Duration of the session: Week 1: 20 min; Week 2: 30 min; Weeks 3–6: 45 min	**Type of AE**: cycling **Intensity**: moderate 50–70% ${\dot{V}}_{O_{2}\max}$ **Follow‐up**: 3 and 6 months	**(1) Participant ratings of global improvement** (GRC) **(2) Pain intensity** (VAS, 0–100 mm) **(3) Neck disability** (NDI, 0–50 points) **(4) Patients’ beliefs relating to the influence of work and activity on their neck pain** (FABQ) **(5) Cervicogenic headache complaints** **(6) Medication consumption for neck pain** **(7) Clinical examination** (i.e., posture assessment)	**(1) GRC** At the 12‐ and 24‐week follow‐up, the GRC scores were significantly higher in the AE group than in the control group (*P* < 0.001). **(2, 3, 4, 5, 6) Pain intensity; NDI; FABQ; cervicogenic complaints and medication consumption** No differences between groups.	No difference between groups was found on pain, NDI, beliefs, cervicogenic complaints, and medication consumption between groups. But the AE group presented better results on the GRC at the 12 and 24 weeks’ follow‐up
	Daher et al. (2021) Funding: the National Insurance Institute of Israel, the Israel Physiotherapy Society and the University of Haifa Setting: outpatient clinic	***n* =** 139 **Age**: 54.6 (10.5) years. **Sex**: mixed **Diagnosis**: non‐specific NP + other MSK complaints **Chronicity**: yes	**Active treatment of interest (*n* = 69)** Strength training + aerobic exercise (STR + AE) TTO: the programme consisted of stretching and muscle performance exercises + AE Duration of intervention: 6 weeks Number of sessions: 24 Frequency: 2×/week supervised and 2× homebased = 4×/weeks Duration of the session: Week 1: 20 min; Week 2: 30 min; Weeks 3–6: 45 min	**Active comparator (*n* = 70)** Strength training alone (STR) TTO: the programme consisted of stretching and muscle performance exercises for neck muscles Duration of intervention: 6 weeks Number of sessions: 2×/week Frequency: 2×/week supervised Duration of the session: Week 1: 20 min; Week 2: 30 min; Weeks 3–6: 45 min	**Type of AE**: cycling **Intensity**: moderate 50–70% ${\dot{V}}_{O_{2}\max}$ **Follow‐up**: 3 and 6 months	**(1) Participant ratings of global improvement** (GRC) **(2) Neck disability** (NDI, 0–50 points) **(3) Work ability** (Hebrew WAI) **(4) Adverse events**	**(1) GRC** At the 12‐ and 24‐week follow‐ups, the GRC scores were significantly higher in the AE group than in the control group (*P* < 0.001). **(2) NDI** No differences between groups. **(3) Work ability** More significant improvements in the AE vs. the control group. **(4) Adverse events** No adverse effects.	No difference between groups was found on NDI, but the GRC and the work ability was better in the group that were treated with AE
	Daher et al. (2022) Funding: the National Insurance Institute of Israel, the Israel Physiotherapy Society and the University of Haifa Setting: outpatient clinic	***n* =** 62 **Age**: 54.9 (SD 10.6) years. **Sex**: mixed **Diagnosis**: non‐specific NP + other MSK complaints **Chronicity**: yes	**Active treatment of interest (*n* = 62)** Aerobic exercise+ strength training (AE + STR) TTO: the programme consisted of stretching and muscle performance exercises + AE Duration of intervention: 6 weeks Number of sessions: 24 Frequency: 2×/week supervised and 2× homebased = 4×/week Duration of the session: Week 1: 20 min; Week 2: 30 min; Weeks 3–6: 45 min	No comparator—secondary analysis focused on treatment of interest group	**Type of AE**: cycling **Intensity**: moderate 50–70% ${\dot{V}}_{O_{2}\max}$ **Follow‐up**: 3 and 6 months	**(1) Participant ratings of global improvement** (GRC) **(2) Neck flexor endurance** (time that the patient maintains pressing the examiner's hand) **(3) Pain** (VAS) **(4) Neck disability** (NDI, 0–50 points) **(5) Patients’ beliefs relating to the influence of work and activity on their neck pain** (FABQ)	They did not explore the between group comparison, just a secondary prediction evaluation.Based on the results three clinical predictor variables were identified, which may help clinicians in determining which patients with non‐specific NP are most likely to benefit from an AE+STR programme: The duration of NP (≤6 months) NFE test result ≥18 s Absence of referred pain	Patients experiencing NP (≤6 months) who have no referred pain and exhibit neck flexor endurance (≥18 seconds) may benefit from a simple self‐training programme of combined AE + STR
3	Eftekharsadat et al. (2018) Funding: no funding or grants Setting: physical medicine and rehabilitation out‐patient clinics	***n* =** 64 **Age**: 33.1 (SD 6.4) **Sex**: mixed (55 F; 9 M) **Diagnosis**: upper trapezius muscle, shoulder girdle, neck, and upper back myofascial pain **Chronicity**: yes	**Active treatment of interest (*n* = 32)** Aerobic exercise + acupuncture (AE + ACU) TTO: Western medical style acupuncture was applied on SI11, SI12, GB20, DU14, DU20, LI10, LI11, LI14, and trigger points in trapezius, levator scapulae, rhomboids, supra and infraspinatus, and paravertebral muscles + AE Duration of intervention: NR Number of sessions: 10 Frequency: 3×/week Duration of the session: 30 min	**Active comparator (*n* = 32)** Acupuncture alone (ACU) TTO: Western medical style acupuncture was applied on SI11, SI12, GB20, DU14, DU20, LI10, LI11, LI14, and trigger points in trapezius, levator scapulae, rhomboids, supra and infraspinatus, and paravertebral muscles + AE Duration of intervention: NR Number of sessions: 10 Frequency: 3×/week Duration of the session: 30 min (ACU) + 50 min (AE)	**Type of AE**: walking on treadmill and using stationarybicycle **Intensity**: high >70% to 75–80% of maximal heart rate. **Follow‐up**: 1 month	**(1) Pain intensity** (VAS, 0–10 mm) **(2) Neck disability** (NDI, 0–50 points) **(3) Pressure pain threshold** (Algometer) **(4) Quality of life** (SF‐36)	**(1) Pain intensity** AE+ACU vs. ACU: MD 7.20 [95%CI: −3.89, 18.29] **(2, 3, 4) Pain intensity; pressure pain threshold and quality of life** No differences between groups.	No difference between groups was found
4	Kocur et al. (2017) Funding: not reported Setting: unclear	***n* =** 39 **Age**: AE = 54.5 (SD 3.7) years; CON = 56.7 (SD 2.9) years **Sex**: female **Diagnosis**: pain in the cervical area **Chronicity**: not reported	**Active treatment of interest (*n* = 20)** Nordic walking (AE) TTO: the NW training was held outdoors, with at least two Nordic walking instructors controlling the marching technique and regulating the pace Duration of intervention: 12 weeks Number of sessions: 36 Frequency: 3×/week Duration of the session: 60 min	**Control (*n* = 19)** Participants were told not to change their movement routines and habits (CON)	**Type of AE**: Nordic walking **Intensity**: low <50%, moderate 50–70% **Follow‐up**: no	**(1) Perceived pain threshold for different muscles of the upper body** (Algometer) **(2) Flexibility of the upper body** (Back Scratch Test)	**(1) Perceived pain threshold for m. trapezius** AE vs. CON: MD 0.60 [95%CI: 0.06, 1.14], *P* = 0.03 **(2) Flexibility of the upper body** No differences between groups.	AE was better than control to improve perceived pain threshold for trapezius muscle
5	Korshøj et al. (2018) Funding: this study was supported by the Danish Medical Research Council Grant 22‐03‐0264 and Danish Rheumatism Association Grant 233‐1149‐02.02.04 Setting: unclear	***n* =** 116 **Age**: 45.3 (SD 8.6) **Sex**: mixed (88 F; 9 M) **Diagnosis**: neck pain + other MSK pain **Chronicity**: not reported	**Active treatment of interest (*n* = 57)** Aerobic exercise (AE) TTO: the following aerobic exercises were initially proposed: aerobics, biking on a stationary bike, treadmill running, and circuit training Duration of intervention: NR Number of sessions: 84 Frequency: 2×/week (but the supervision of AE declined gradually) Duration of the session: 30 min	**Reference group (*n* = 59)** Education (EDU) TTO: the lectures, only offered to the reference group, addressed healthy living, but not physical activity, and participants were invited to suggest topics. The following topics were initially proposed: physiologic response of stress, healthy diet, and smoking cessation Duration of intervention: NR Number of sessions: 3 Frequency: NR Duration of the session: 2 h	**Type of AE**: biking on a stationary bike, treadmill running, and circuit training. **Intensity**: moderate = ⩾60% ${\dot{V}}_{O_{2}\max}$ **Follow up**: 4 and 12 months	**(1) Musculoskeletal symptoms** (Nordic Questionnaire)	**(1) Musculoskeletal symptoms** 4 m follow‐up: AE vs. EDU: MD 0.18 [95%CI: −1.39, 1.75], *P* = 0.85 12 m Follow‐up: AE vs. EDU: MD 1.04 [95%CI: −1.23, 3.31], *P* = 0.37	No difference between groups was found
6	Saeterbakken et al. (2017b) Funding: not reported Setting: unclear	***n* =** 34 **Age**: AE = 41 (SD 15.3) years SST = 47.6 (SD 11.9) years CON = 50.3 (SD 14.8) years **Sex**: mixed **Diagnosis**: neck and shoulder pain **Chronicity**: not reported	**Active treatment of interest (*n* = 10)** Nordic walking (AE) TTO: the instructor corrected the technique according to the International Nordic Walking Federation recommendation. Duration of intervention: 10 weeks Number of sessions: 20 Frequency: 2×/week Duration of the session: 30 min	**Active comparator (*n* = 13)** Specific strength training (SST) TTO: five exercises for neck/shoulder muscles with elastic bands (ropes) Duration of intervention: 10 weeks Number of sessions: 20 Frequency: 2×/week Duration of the session: 30 min **Control (*n* = 11)** No intervention (CON)	**Type of AE**: Nordic walking **Intensity**: the intensity of the first 5 min was gradually increased to 12 RPE (light) and increased to 12–14 RPE (light to moderate intensity) for the remaining 25 min **Follow up**: 10 weeks	**(1) Pain intensity** (VAS, 0–100 mm) (a) Prolonged effect: general pain (b) Acute effect: different time points before and after training sessions **(2) Shoulder elevation strength** (force cell) **(3) Aerobic fitness** (6MWT)	**(1) Pain intensity** AE vs. STR: MD −4.22 [95%CI: −19,46, 11.02], *P* = 0.59 AE vs. CON: MD 6.24 [95%CI: −11.21, 23.69]; *P* = 0.48 STR vs. CON: MD 10.46 [95%CI: −7.02, 27.94]; *P* = 0.24	No difference between groups was found

Abbreviations: 6MWT, 6‐minute walk test; ACU, acupuncture; AE, aerobic exercise; CI, confidence interval; CON, control; EDU, education; EMG, electromyography; F, female; FABQ, fear avoidance beliefs questionnaire; GRC, global rating of change scale; M, male; m, month; min, minutes; MD, mean difference; MMG, mechanomyography; MSK, musculoskeletal; MVIS, maximal voluntary isometric muscle strength; NDI, neck disability index; NFE, neck flexor endurance; NIRS, near infrared spectroscopy; NP, neck pain; NR, not reported; SD, standard deviation; SST, specific strength training; TTO, treatment definition; VAS, visual analogue scales; w, weeks; WAI, work ability index. *Change pre‐post intervention. **P‐value <0.05 was considered as statistically significant.

### 3.3 Risk of bias assessment of included studies

**FIGURE 2:**
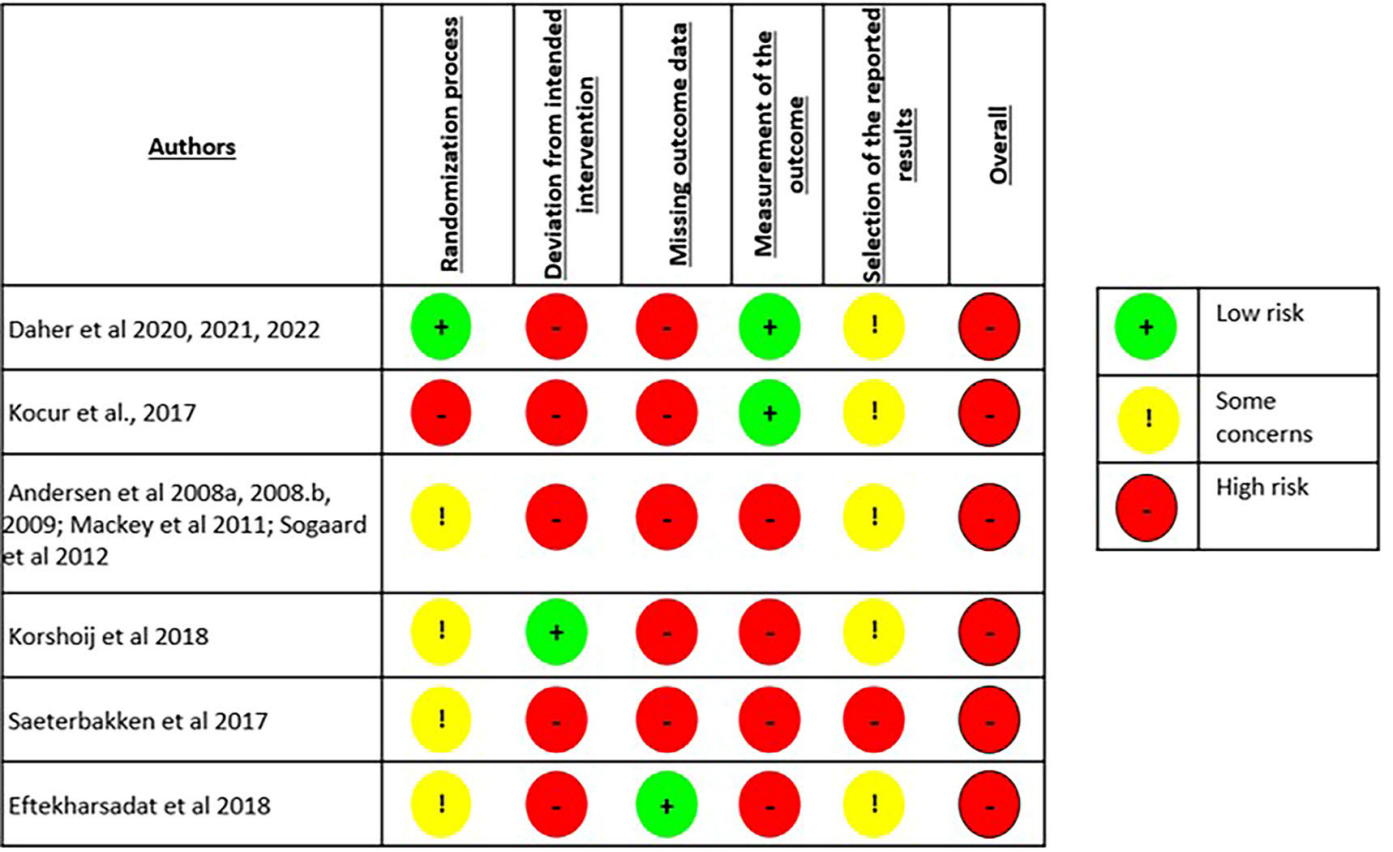
Risk of bias assessment of the studies included.

**FIGURE 3:**
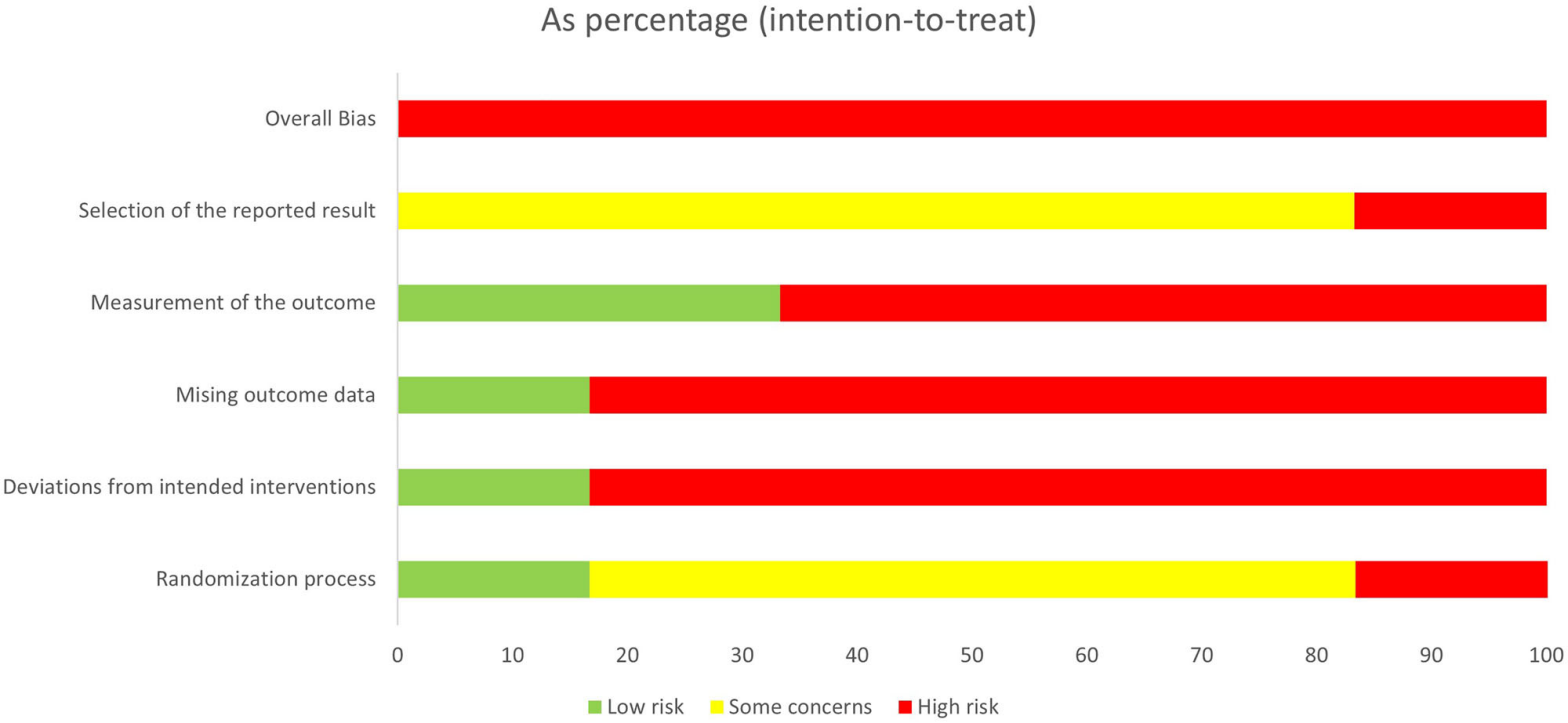
Summary of the risk of bias assessment of the studies included.

All included studies presented a high RoB (Figures 2 and 3). When looking at the compiled set of items, most studies could only meet slightly less than half of the items (between 37.5% and 82.5%) (more details in Supporting information, Appendix 4, Table A4). The main issues arose most frequently from lack of intention‐to‐treat analysis and blinding. Blinding of the investigator and therapist was not performed in any of the studies, and only two of the six studies met sub‐items such as a method of adequate blinding or blinding of the assessors (Daher et al., 2022, 2020, 2021; Kocur et al., 2017). Only one study (Daher et al., 2022, 2020, 2021) described the intervention as double‐blinded, with both investigators and participants blinded.

Another crucial point is that although dropout rates were described, only three (42.8%) studies reported explanations of them, and these individuals were often not included in the follow‐up analyses (Daher et al., 2022, 2020, 2021; Eftekharsadat et al., 2018; Korshøj et al., 2018). Although all studies were described by the authors as randomized, only half of them described the method in sufficient detail (Daher et al., 2022, 2020, 2021; Eftekharsadat et al., 2018; Korshøj et al., 2018, and just two (28.6%) studies applied a concealed method of allocation (Daher et al., 2022, 2020, 2021; Korshøj et al., 2018). In addition, both co‐interventions and adverse effects were often not mentioned. RoB was one of the main reasons for downgrading the quality of evidence to be low or very low based on the GRADE (Supporting information, Appendix 5, Table A5).

### 3.4 Aerobic exercise interventions in individuals with CNP

The total number of individuals from all included studies was 445, ranging from 34 to 139 individuals for each study, with a significantly higher proportion of women (about 80%). Two studies (six articles) (Andersen et al., 2009; Andersen, Andersen et al., 2008; Andersen, Jørgensen, et al., 2008; Eftekharsadat et al., 2018; Mackey et al., 2011; Sogaard et al., 2012) recruited individuals with trapezius myalgia, and three studies (five articles) (Daher et al., 2022, 2020, 2021; Korshøj et al., 2018; Saeterbakken et al., 2017) included multiple MSK complaints (lumbar spine, hip, and knee). However, the analysis was performed separately for NP. Only one study (Kocur et al., 2017) included individuals exclusively with NP (Table 2).

Different types and protocols of AE were used, such as Nordic walking (Kocur et al., 2017; Saeterbakken et al., 2017), cycling with legs only (Andersen et al., 2009; Andersen, Andersen et al., 2008; Andersen, Jørgensen, et al., 2008; Daher et al., 2022, 2020, 2021; Mackey et al., 2011; Sogaard et al., 2012), and a combination of stationary cycling and walking on a treadmill (Eftekharsadat et al., 2018). Particularly, one study offered different aerobic training modalities in which individuals could choose between cycling on a stationary bike, running on a treadmill, or functional training (e.g., a combination of running, balance exercises, and endurance) (Korshøj et al., 2018). These intervention groups were compared with active groups, control groups (no exercise), or both (control and active comparators). The number of intervention groups varied between two (Daher et al., 2022, 2020, 2021; Eftekharsadat et al., 2018; Kocur et al., 2017; Korshøj et al., 2018) and three (Andersen et al., 2009; Andersen, Andersen, et al., 2008; Andersen, Jørgensen, et al., 2008; Mackey et al., 2011; Saeterbakken et al., 2017; Sogaard et al., 2012). In four studies (Andersen et al., 2009; Andersen, Andersen, et al., 2008; Andersen, Jørgensen, et al., 2008; Kocur et al., 2017; Korshøj et al., 2018; Mackey et al., 2011; Saeterbakken et al., 2017; Sogaard et al., 2012) AE was performed in isolation and compared with strength exercises, education therapy or control groups. In two studies (Daher et al., 2022, 2020, 2021; Eftekharsadat et al., 2018) the objective was to determine the additional effects of AE when added to other interventions (strengthening or acupuncture) in individuals with NP. Therefore, it was possible to analyse the effects of AE.

A few variations were observed for measurement tools between studies. Four studies (Andersen et al., 2009; Andersen, Andersen, et al., 2008; Andersen, Jørgensen, et al., 2008; Daher et al., 2022, 2020, 2021; Eftekharsadat et al., 2018; Mackey et al., 2011; Saeterbakken et al., 2017; Sogaard et al., 2012) measured pain intensity in the neck with a visual analogue scale (VAS) and one study (Korshøj et al., 2018) measured it with a modified version of the Standardized Nordic Questionnaire. Secondary outcomes varied between the studies, exploring physical or emotional status measurements (Supporting information, Appendix 6). The duration of the intervention was not always precisely stated; most studies chose from six to 12 weeks of treatment.

We found little overlap (e.g., commonality) between interventions and outcomes across the six studies, indicating a high degree of heterogeneity across studies (Table 2).

### 3.5 Primary outcomes

#### 3.5.1 Pain intensity

##### 3.5.1.1 Aerobic exercise versus non‐treatment (end of the treatment and 10 weeks follow‐up)

**FIGURE 4:**
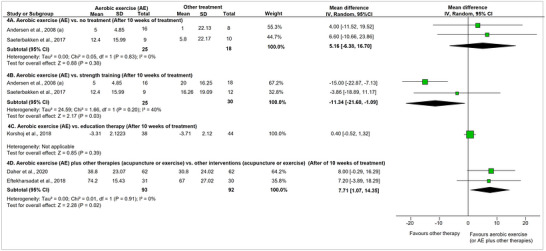
Meta‐analysis of pain intensity between aerobic exercise (AE) training and other therapies. All analyses are described as mean differences in mm with random effects. The pain was measured with a Visual Analog Scale (100 mm). The effect sizes for comparison 4A, 4B, and 4D were calculated using mean change before‐after the treatments. Higher numbers mean larger pain improvement between before and after treatment. The effect size for comparison 4C used only the mean at the end of the treatment.

**FIGURE 5:**
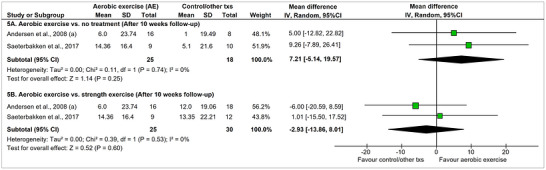
Meta‐analysis of pain intensity between aerobic exercise training and other therapies after 10 weeks’ follow‐up. All analyses are described as mean differences in mm with random effects. The pain was measured with a Visual Analog Scale (100 mm). The effect sizes were calculated using mean change before‐after the treatments (txs: treatments). Higher numbers mean larger pain improvement between before and after treatment.

Two studies (Andersen, Andersen, et al., 2008; Saeterbakken et al., 2017; Søgaard et al., 2012) compared the effectiveness of aerobic exercise (Nordic walking, moderate intensity, 30 min, 2×/week, or leg cycling, moderate intensity, 20 min, 3×/week) versus a *control group* (no treatment) on pain intensity (VAS). None of them found a statistically significant difference between groups after the intervention, and when both studies were combined in a meta‐analysis, no significant differences were verified between them at the end of the intervention (after 10 weeks of treatment) (MD [95%CI] 5.16 mm [−6.38, 16.70]) (Figure 4A) or at 10 weeks’ follow‐up (MD [95%CI]: 7.21 mm [−5.14, 19.57]) (Figure 5A). The overall certainty of the evidence for these comparisons was low according to the GRADE assessment (Supporting information, Appendix 5, Table A5.1 and A5.2).

##### 3.5.1.2 Aerobic exercise versus strength exercise (end of the treatment and 10 weeks’ follow‐up)

Two studies (reported in five articles) (Andersen et al., 2009; Andersen, Andersen, et al., 2008; Andersen, Jørgensen, et al., 2008; Saeterbakken et al., 2017; Sogaard et al., 2012) were included in this comparison. Saeterbakken et al. (2017b) compared *aerobic exercise* (Nordic walking, moderate intensity, 30 min, 2×/week, 10 weeks) with *strength exercise* (five exercises for neck/shoulder muscles with elastic bands, 30 min, 2×/week, 10 weeks) and found no statistically significant difference on pain intensity between them at the end of the treatment (Figure 4B) or at 10 weeks’ follow‐up (Figure 5B). In contrast, Andersen, Kjaer et al. (2008) reported that *strength exercises* (five dumbbell exercises specifically for the shoulder/neck muscles, 20 min, 3×/week, for 10 weeks) were significantly superior to *aerobic exercise* (leg bicycling, moderate intensity, 20 min, 3×/week, for 10 weeks) regarding its effect on pain intensity at the end of the treatment (Figure 4B), but this difference was no longer significant at 10 weeks’ follow‐up (Figure 5B). The pooled effect of these two studies on pain intensity outcome at the end of the treatment showed a positive improvement in pain intensity favouring the strength exercise (MD [95%CI]: −11.34 mm [−21.6, −1.09]) (Figure 4B), while no difference between the groups was verified at 10 weeks’ follow‐up (MD [95%CI]: −2.93 mm [−13.86, 8.01]) (Figure 5B). The overall certainty of the evidence for these comparisons was very low according to the GRADE assessment (Supporting information, Appendix 5, Table A5.3 and A5.4).

##### 3.5.1.3 Aerobic exercise vs. education therapy (end of the treatment)

One study (Korshøj et al., 2018) compared *aerobic exercise* (leg bicycling, moderate intensity, 30 min, 2×/week, 10 weeks) vs. *education therapy* (health‐promoting activities, 60 min, 1×/week, for 10 weeks). Based on this result, *aerobic exercise* did not present a significant superiority at the end of the treatment when compared to *education therapy* (MD [95%CI]: 0.40 mm [−0.52, 1.32]) (Figure 4C). The overall certainty of the evidence for these comparisons was low according to the GRADE assessment (Supporting information, Appendix 5, Table A5.5).

##### 3.5.1.4 Aerobic exercise plus other therapies versus other active interventions (end of the treatment)

Two studies compared the combined effect of *AE* plus *acupuncture* (Eftekharsadat et al., 2018) or *AE* plus *strength training* with *acupuncture* or *strength training* alone (Daher et al., 2020; Eftekharsadat et al., 2018). Both studies reported that the combined training was better than the isolated training alone in reducing pain intensity after treatment (pooled effect: MD [95%CI]: 7.71 mm [1.07, 14.35]) (Figure 4D). According to the GRADE assessment, the overall certainty of the evidence for these comparisons was low (Supporting information, Appendix 5, Table A5.6).

#### 3.5.2 Physical function: Disability measured with neck disability index

##### 3.5.2.1 Aerobic exercise plus strength exercise or acupuncture versus strength exercise alone or acupuncture alone (end of treatment and 4 weeks’ follow‐up)

**FIGURE 6:**
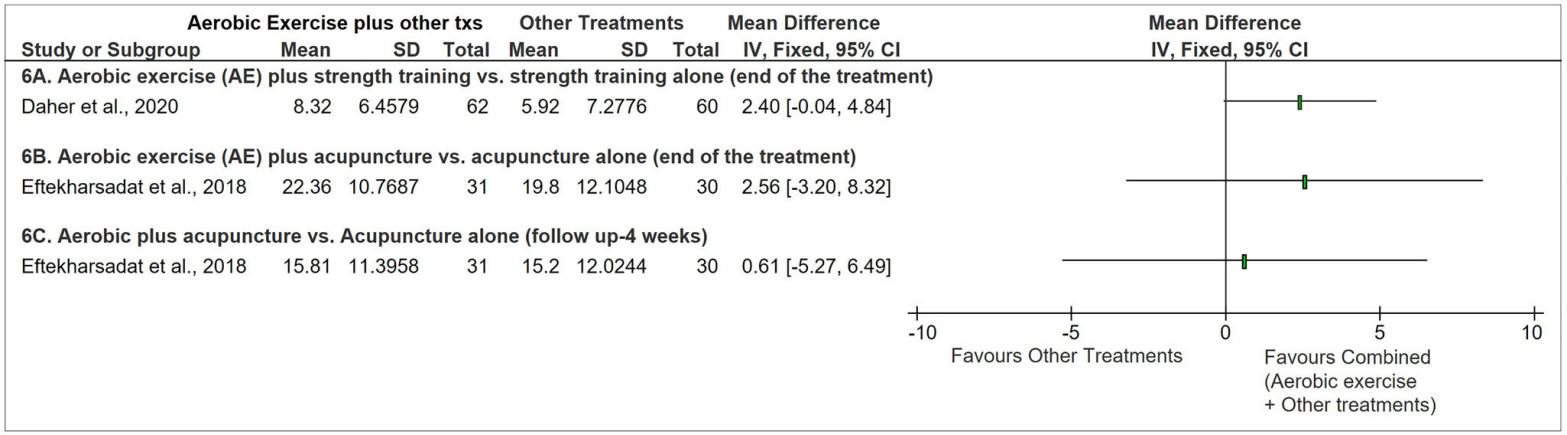
Treatment effects for neck disability comparing aerobic exercise training plus other treatments vs. other therapies alone at the end of the treatment and at 4 weeks’ follow‐up. All analyses are described as mean differences in points with fixed effects. The effect sizes were calculated using the mean change before and after the treatments. Higher numbers mean larger neck disability improvement between before and after treatment; txs: treatments

Two studies (in three articles) (Daher et al., 2020, 2021; Eftekharsadat et al., 2018) looked at the combined effect of *AE* (walking on a treadmill or leg bicycling, high intensity, 30 min, 3×/week, for four weeks OR cycling, moderate intensity, 30–45 min, 2×/week, for six weeks) plus *strength exercises* or *acupuncture* versus the isolated effect of these two therapies on neck disability. Daher et al. (2021) evaluated the effect of *combined therapy* (AE plus strength exercise) versus *strength exercise* (stretching and muscle performance exercises for neck muscles, 30–45 min, 2×/week, for six weeks) alone. Although statistically significant differences were not found between groups for NDI at the end of the treatment (MD [95%CI]: 2.40 points [−0.04, 4.84]) (Figure 6A), the combined therapy was better considering the confidence intervals (since both articles presented the same population and the same results, we just presented the results from the first publication in the forest plot). Eftekharsadat et al. (2018) evaluated the effect of *combined therapy* (Nordic walking, moderate intensity, 30 min, 2×/week, 10 weeks plus acupuncture) versus *acupuncture* alone (acupuncture on neck muscles 30 min, 3×/week, 10 weeks), and found non‐statistically significant differences between groups (MD [95%CI]: 2.56 points [−3.20, 8.32]) after treatment (Figure 6B). This result was maintained at 4 weeks’ follow‐up (Figure 6C).

### 3.6 Secondary outcomes

Secondary outcomes are described in Supporting information, Appendix 6.

A summary of the effects of AE on pain intensity, disability, and secondary outcomes is reported in Table 3.

**TABLE 3:** Summary of the results.

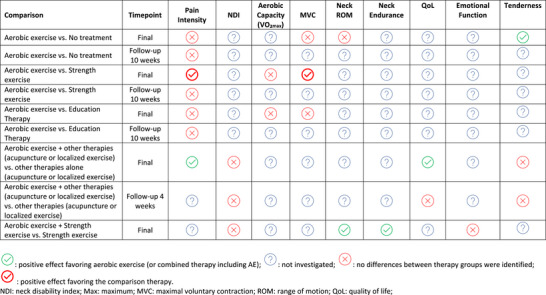

### 3.7 Overall quality/certainty of the evidence

Comparisons were made according to interventions and outcomes, but because of a lack of similarities among studies, estimates from different studies were not pooled for most of the outcomes due to different interventions and heterogeneity of the data. Due to the high heterogeneity of the outcomes, only pain intensity was evaluated using GRADE. The overall quality (certainty) of the evidence was rated very low for most of the comparisons (Supporting information, Appendix 5, Table A5). The evidence was generally downgraded due to RoB and imprecision of results. No high‐quality evidence was found in the included studies, suggesting that the effectiveness of AE in individuals with NP remains uncertain.

## 4 DISCUSSION

In summary, AE alone was no better than strengthening exercises or no treatment to reduce pain intensity in individuals with NP. Nonetheless, when combined with other therapeutic modalities, AE could potentially reduce pain intensity and improve neck disability. However, when looking at the effect of AE versus control or other intervention modalities, high heterogeneity across study results was observed, with different magnitudes and directions. Despite these results, primary studies reported AE to be beneficial for improving physical and emotional status, similar to strength training (see Supporting information).

### 4.1 Proposed physiological mechanisms that impact pain and disability

Inflammation and oxidative stress are closely linked to chronic pain through sensitization of the central nervous system. Inflammatory mediators (e.g., cytokines) are generally increased in patients with chronic pain and act directly in the nociceptors, lowering pain thresholds (Woolf & Ma, 2007). The positive effects of exercise on pain occur through several physiological processes, which are controlled by the central nervous, hormonal, and opioid and non‐opioid systems (De la Corte‐Rodriguez et al., 2024).

The opioid system consists of widely scattered neurons that produce analgesia through the release of endogenous opioids (β‐endorphin, met‐ and leu‐enkephalins and dynorphins), which act as neurotransmitters and neuromodulators (Holden et al., 2005). It has been reported that exercise, especially high‐intensity AE, increases the levels of β‐endorphin and met‐enkephalin in the hypothalamus, periaqueductal grey, and rostral ventromedial medulla, which promotes pain relief (De la Corte‐Rodriguez et al., 2024). Regular exercise also increases serotonin levels and opioids in the central pathways, improving the descending inhibitory pathways and reducing central facilitation, and improving pain and disability (De la Corte‐Rodriguez et al., 2024; Lima et al., 2017; Nicolini et al., 2021). Another hypothesis that supports exercise‐induced hypoalgesia integrates the opioid with the endocannabinoid system (De la Corte‐Rodriguez et al., 2024). It has been demonstrated that cannabinoid type 2 agonists (e.g., CB2) produce synergistic antinociceptive effects with opioids releasing more pain relief substances after exercise, especially after endurance or long‐term exercise (De la Corte‐Rodriguez et al., 2024; Finn et al., 2021).

The hormonal system (especially through progesterone) may play an important role in pain control through exercise (Jaleel et al., 2022). It has been reported that the level of progesterone concentration increases after exercise, which has an anti‐inflammatory effect by regulating prostaglandins and leukotrienes, modulating pain circuits, and promoting hypoalgesia after exercise (Coronel et al., 2011; De la Corte‐Rodriguez et al., 2024). Furthermore, reduced cortisol levels and release of endorphins are stimulated through exercise and interact with brain receptors to reduce pain perception and lower systemic inflammation (De la Corte‐Rodriguez et al., 2024; Lima et al., 2017; Nicolini et al., 2021).

The postulated mechanisms by which regular AE reduces the effects of inflammation in the long‐term includes an increase of anti‐inflammatory cytokines (e.g., interleukin (IL)‐10, IL‐4, and transforming growth factor‐β), which decreases pro‐inflammatory C‐reactive protein in several tissues such as skeletal muscle, adipose tissue, central nervous system, and immune cells (Casana et al., 2022; Gleeson, 2013). Exercise not only increase the blood flow to the neck muscles and surrounding tissues (Brooks, 1986; Coyle, 2000), but also increase the general blood circulation, facilitating clearance of lactic acid (Gladden, 2004), carbon dioxide (Guyton & Hall, 2006), and inflammatory cytokines (e.g., tumour necrosis factor‐α (TNF‐α), IL‐6 and IL‐1β). The cytokine profile of individuals who exercise regularly and for longer periods may potentially change, although this is still a matter of debate (Docherty et al., 2022). Pro‐inflammatory cytokines such as TNF‐α and IL‐6 are elevated in the presence of high levels of adipose tissue and visceral fat, which are potentially reduced following a regular AE programme, indirectly contributing to decreasing pain (Monsalve et al., 2023).

Based on the above‐mentioned mechanisms, regular AE emerges as a potential intervention to mitigate the effects of chronic pain. However, the exact exercise type and dosage (i.e., duration, intensity, and frequency) to maximize these effects are still unclear.

### 4.2 Effectiveness of aerobic exercise on pain intensity in a short‐term follow‐up

The intensity of pain in the pre–post comparisons measured with a VAS showed mixed results. In a within‐group comparison, AE (cycling) provided positive results in reducing NP, when applied in isolation or in combination with education therapy. This result corroborates the hypothesis that AEs performed with the lower extremities (e.g., cycling with the legs) could also benefit non‐exercising muscle groups, leading to hypoalgesia in remote areas (Vægter et al., 2018). This effect could be particularly useful for those individuals who are afraid to move (i.e., individuals with kinesiophobia). In contrast, strength training showed a better effect than AE alone for NP intensity (Andersen et al., 2009; Andersen, Andersen, et al., 2008; Andersen, Jørgensen, et al., 2008; Saeterbakken et al., 2017; Sogaard et al., 2012). Most of the studies used moderate‐intensity training, which could partially explain the discrete effects of AE; only one study presented high‐intensity training as a basis. It is still uncertain whether higher intensities of AE could be similar to or better than other modalities to reduce pain, as shown in other areas (e.g., migraine) (Lemmens et al., 2019).

### 4.3 Effectiveness of aerobic exercise on pain intensity in a long‐term follow‐up

The benefits of AE in a long‐term follow‐up are controversial. Some studies found that the positive benefits of AE were not sustained during the follow‐up period, neither when the AE was compared to a control treatment nor compared to strength training (Andersen et al., 2009; Andersen, Andersen, et al., 2008; Andersen, Jørgensen, et al., 2008; Sogaard et al., 2012). However, two studies reported positive results in a long‐term follow‐up (10 and 12 weeks) (Korshøj et al., 2018; Saeterbakken et al., 2017). It is important to highlight that in the study from Saeterbakken et al. (2017) both groups were encouraged to continue exercising at home. Furthermore, Korshøj et al. (2018) also confirmed the positive effects of AE on pain intensity after a long period follow‐up (4‐ and 12‐month follow‐ups) using the Nordic Questionnaire at 4‐ and 12‐month follow‐ups. Thus, it is not entirely clear whether the long‐term effects are maintained due to physiological mechanisms or because the individuals continued to perform AE independently after the end of the supervised treatment.

### 4.4 Effectiveness of aerobic exercise on physical and emotional status

As previously mentioned, the literature has demonstrated that AE has positive (short‐ and long‐term) effects on psychological outcomes (Saeterbakken et al., 2017) (Appendix 5). The Fear Avoidance Belief Questionnaire, the Quality of Life 36‐Item Short Form Survey, and the 15‐item Global Rating of Change (GRC) showed highly positive results for the addition of AE, but just in the within‐group comparison; when compared with other therapies, no differences were identified. Remarkably, the GRC scores improved over time, along with VAS scores, showing a clinically relevant improvement for the within‐group comparisons. Also, the combined therapy (AE plus acupuncture or localized exercise) demonstrated a greater effect than AE alone on QoL. The improvement in psychological factors supports that AE reduces fatigue and improves health‐related quality of life (Dinler et al., 2009).

### 4.5 Effectiveness of aerobic exercise on tenderness

Just results from individual studies could be explored, and no superiority was found after AE for neck muscle tenderness when compared to other types of treatment (e.g., acupuncture and routine activities) (Eftekharsadat et al., 2018; Kocur et al., 2017). One reason for these results may be that both types of exercises create a healthy biophysical muscle tone because they provide adapted sympathetic and local metabolic regulation of blood flow, which could potentially lead to the use of all available capillaries, expand microcirculation, and increase aerobic capacity, which in turn can influence muscle tenderness.

### 4.6 Comparison with previous reviews

Just one previous systematic review looking specifically at the effect of AE on clinical outcomes for individuals with NP has been published (Paraskevopoulos et al., 2023). The authors found similar results as presented in this systematic review, that AE may be effective in the management of non‐specific NP; however, they did not explore the results quantitatively and did not include all the studies included in the present review, which limits comparisons. In another systematic review considering all forms of treatment, AE was compared with a reference group (keep normal activities) after ten weeks of intervention and ten weeks of follow‐up (Gross et al., 2015). No statistical nor clinically significant differences between the groups were found. These results and the certainty of the evidence for this previous review were very low, which was consistent with the findings of this systematic review.

A recent network meta‐analysis (NMA) of 40 RCTs compared the effectiveness of different exercise interventions for chronic non‐specific NP, such as prescribed physical activity, range of motion, balance, strengthening, stretching combined and multimodal exercises (de Zoete, Armfield, et al., 2020). This NMA found small effects with wide confidence intervals indicating inconsistency and uncertainty in the interpretation of findings, which agrees with our results. This NMA also did not use AE as a node (e.g., treatment of interest) and only included prescribed exercise activity. This result highlighted the need to closely analyse supervised AEs in comparison with other treatment strategies to provide evidence for their use in clinical practice to manage individuals with NP.

### 4.7 Methodological bias and quality of evidence

The most frequently methodological missing items in the selected studies included the lack of blinding and the absence of the intention to treat (ITT) analysis. These have been acknowledged as significant sources of bias (e.g., selection, detection, performance, etc.) and are thought to affect the internal validity of a trial and the accuracy of treatment effect estimates (TEE) (Armijo‐Olivo et al., 2022; Moustgaard et al., 2020; Saltaji et al., 2018).

Missing data are also problematic in research. The ITT analysis has been proposed as a gold standard strategy to analyse RCT results (Ruiz‐Canela et al., 2000). However, in this review, only two studies conducted an ITT analysis (Daher et al., 2022, 2020, 2021; Eftekharsadat et al., 2018). Recent evidence shows that trials that did not use the ITT or were assessed as having inappropriate control of incomplete outcome data tended to underestimate the TEE when compared with trials with adequate ITT and control of incomplete outcome data (Armijo‐Olivo et al., 2022).

The lack of the above‐mentioned methodological items and inconsistencies in results between studies downgraded the quality of the evidence to very low. No high‐quality evidence was found in the included studies, indicating a lot of uncertainty about the effectiveness of AE in individuals with NP compared to other therapies.

### 4.8 Strengths and limitations of this review

Despite this review focusing on all NP (acute, subacute, and CNP), only two studies presented information about the diagnosis duration, and both included chronic pain. Hence, a subgroup analysis based on the chronicity of pain was not possible (Daher et al., 2022, 2020, 2021; Eftekharsadat et al., 2018). Most of the individuals in this review were women, which means uncertainty of the effects of AE for men. Although the AE groups used a similar definition, only a few types of AE exercises (e.g., walking or cycling) and intensity (e.g., moderate intensity (50–70% heart rate (HR))) were applied. Thus, the effects of AEs in the form of sports (e.g., swimming, dancing, or others) could not be assessed. Due to the high heterogeneity of intervention groups and outcomes, just a few results could be pooled in a meta‐analysis, which greatly reduced the ability to determine the magnitude of the effect of AE when compared to other interventions. In addition, the certainty of the evidence using GRADE was only evaluated for our main outcome (pain intensity). Due to the few studies included, exploration of publication bias was not feasible; however, our searches were comprehensive and performed by a professional librarian, which reduced the likelihood of such a bias in our review.

### 4.9 Implications for future research

Because NP is highly prevalent and contributes to public health expenditure, the impact of AE on this condition requires further research. Better quality RCTs that are well‐designed, with larger sample sizes and sufficient long intervention and follow‐up periods, are needed to adequately investigate the long‐term effects of AE. The participants need an accurate musculoskeletal diagnosis that considers the different types of NP and, most importantly, differentiates between acute, subacute, and chronic stages.

Another point is that most studies applied AE using moderate intensity. Evidence reports that intensity is a crucial factor for exercise‐induced hypoalgesia (Hoffmann et al., 2012). Applying different intensities at different stages of the intervention period is a topic of future investigation in this area. Additionally, further studies with better exercise protocol guidelines (e.g., type of aerobic exercise, frequency, duration) are needed.

### 4.10 Implications for clinical practice

The results of this systematic review show limited evidence and further research is needed to make accurate recommendations. Nevertheless, AE was slightly better than control. This can be a good option for individuals who do not like strength training or are afraid to move their neck. Moreover, no additional equipment is needed for many types of AE. It is, therefore, a simple and cost‐effective treatment method with an additional positive impact on quality of life and well‐being (Irby et al., 2016; Kroll et al., 2018). In addition, the combination of AE and strengthening exercises may significantly improve pain.

### 4.11 Conclusion

This systematic review aimed to determine the effectiveness of AE in individuals with neck pain compared to control or other treatments. Many advantages of the application of AE were found, even though the findings were of low‐quality evidence and very low certainty. AE was found to be equally effective as other active treatment modalities and could be used to reduce pain in individuals with neck pain. In addition, it can also improve disability, physical function (functionality), and emotional status (within treatment). The combination of AE with other treatments seems promising in reducing pain intensity. In addition, AE is simple to perform, versatile, less costly, and can be applied in groups. It can also have potential positive effects on the general health status, cardiovascular system, sleep quality, mental health, and general well‐being. Nonetheless, the results of this review should be considered with caution, as further research is needed for accurate results.

## AUTHOR CONTRIBUTIONS

Ana Izabela Sobral de Oliveira‐Souza: Conception or design of the work; acquisition, analysis, and interpretation of data for the work; drafting of the work or revising it critically for important intellectual content. Marie Kempe: Acquisition, analysis, and interpretation of data for the work; drafting of the work or revising it critically for important intellectual content. Sofia Grimmelsmann: Acquisition, analysis, and interpretation of data for the work; drafting of the work or revising it critically for important intellectual content. Luiz Felipe Tavares: Acquisition, analysis, and interpretation of data for the work; drafting of the work or revising it critically for important intellectual content. Angela Viegas Andrade: Acquisition, analysis, and interpretation of data for the work; drafting of the work or revising it critically for important intellectual content. Ester Moreira De Castro‐Carletti: Acquisition, analysis, and interpretation of data for the work; drafting of the work or revising it critically for important intellectual content. Liz Dennett: Conception or design of the work, data acquisition, critically revision of the last version of the manuscript for important intellectual property. Harry Von Piekartz: Conception or design of the work; drafting of the work or revising it critically for important intellectual content. Jorge Fuentes Contreras: Conception or design of the work; drafting of the work or revising it critically for important intellectual content. Susan Armijo‐Olivo: Conception or design of the work; acquisition, analysis, and interpretation of data for the work; drafting of the work or revising it critically for important intellectual content. All authors declare that they have approved the final version of the article and agreed to be accountable for all aspects of the work in ensuring that questions related to the accuracy or integrity of any part of the work are appropriately investigated and resolved, and that and all persons designated as authors qualify for authorship, and all those who qualify for authorship are listed. The authors declare that the results of the study are presented clearly, honestly, and without fabrication, falsification, or inappropriate data manipulation. Research governance: Svenja Knüppe, Hochschule Osnabrueck, University of Applied Sciences; Email: s.knueppe@hs‐osnabrueck.de.

## CONFLICT OF INTEREST

None declared.

## FUNDING INFORMATION

None.

## Supporting information



Appendix 1 – Search strategy.
Appendix 2 – Diagnosis description.
Appendix 3 – List of excluded studies.
Appendix 4 – Summary of Compiled Set of Items.
Appendix 5 – GRADE approach results.
Appendix 6 – Secondary outcomes results.

## Data Availability

All the data used by this review is available upon request.
