# Supplementary material for: The effectiveness of aerobic exercise on pain and disability in individuals with neck pain: A systematic review and meta‐analysis
Source: Exp Physiol. 2024 Nov 19;111(6):2918–44. doi: 10.1113/EP091884 (PMC13238618; doi:10.1113/EP091884)
Supplement: Supplementary file 1 — Appendix 1 – Search strategy. Appendix 2 – Diagnosis description. Appendix 3 – List of excluded studies. Appendix 4 – Summary of Compiled Set of Items. Appendix 5 – GRADE approach results. Appendix 6 – Secondary outcomes results. [file EPH-111-2918-s001.docx]

**Appendix 1 – Search strategy**

**Ovid MEDLINE(R) ALL <1946 to April 13, 2023> (OVID Interface)**

Date searched: April 13, 2023

Results: 778

1 exp craniomandibular disorders/ or exp temporomandibular joint disorders/ 19029

2 neck injuries/ or whiplash injuries/ 8642

3 (Intervertebral Disc Degeneration/ or Intervertebral Disc Displacement/) and (exp neck/ or (neck or cervical or cervicothoracic or cervicogenic or craniocervical).mp.) 4206

4 facial pain/ or neck pain/ 15330

5 (exp Temporomandibular Joint/ or exp Masticatory Muscles/ or ((Masticatory or Masseter or pterygoid or Temporal) adj2 Muscl*).mp.) and (pain* or ache* or discomfort or sore*).mp. 5293

6 (exp Myofascial Pain Syndromes/ or (myofascial adj2 pain).mp.) and (face or facial or orofacial or craniofacial or jaw or head or neck or mandib* or craniomandibular).mp. 3714

7 ((Pain* or ache* or discomfort* or injur* or sore* or tender* or agony or agonies or excruciat* or tear or tears or injur* or sprain* or strain* or contracture* or dislocation* or cramp* or impingement or instabilit*) adj8 ((head not head injur*) or cranial or facial or orofacial or face or jaw or mandibular or craniomandibular or tmj or temporomandibular joint or neck or cervical or craniocervical or cervicogenic or cervicothoracic or Cx)).mp. 112528

8 (chronic pain or acute pain or subacute pain or intractable pain or musculoskeletal pain or noncancer pain or non-cancer pain or non-malignant pain or nonmalignant pain or nonneoplastic pain or non-neoplastic pain or myofascial trigger point*).mp. and (exp neck/ or exp head/) 382

9 (neckache* or whiplash or (neck adj4 complaint*) or Orofacial migraine or burning mouth syndrome or cervicodynia or cervicalgia or ((temporomandibular or craniomandibular) adj4 (disorder* or disease* or syndrome* or pain*)) or tmj or tmd or costen* or ((neck or cervical or cervicothoracic or cervicogenic or craniocervical) and (radiculopathy or zygapophyseal joint syndrome or facet joint syndrome or Intervertebral Disc Degeneration or Intervertebral Disc Displacement or disc hernia* or herniated disc*))).mp. 43277

10 (neck disability index or Copenhagen Neck functional disability scale).mp. 2965

11 or/1-10 143525

12 (osteoporo* or RA or arthritis or ankylosing spondylitis or SCI or spinal cord* or spin* injur* or parapleg* or quadripleg* or lupus or (cancer* not non-cancer) or (malignan* not non-malignan*) or oncolog* or neoplasm* or tumour* or tumor* or burn or burns or abuse* or donor or copd or chronic obstructive pulmonary disease or stroke).ti. 2567628

13 exp *Spinal Cord Injuries/ or exp *Osteoporosis/ or exp *Neoplasms/ or *arthritis, experimental/ or *arthritis, infectious/ or *arthritis, juvenile/ or *arthritis, psoriatic/ or exp *arthritis, rheumatoid/ or exp *rheumatic fever/ or *sacroiliitis/ or exp *spondylarthritis/ 3635331

14 exp *Lupus Erythematosus, Discoid/ or exp *Lupus Vasculitis, Central Nervous System/ or exp *Lupus Erythematosus, Cutaneous/ or exp *Lupus Nephritis/ or exp *Lupus Erythematosus, Systemic/ 58307

15 exp *Lung Diseases, Obstructive/ 195911

16 11 not (or/12-15) 124191

17 physical conditioning, human/ or circuit-based exercise/ or endurance training/ or high-intensity interval training/ or running/ or jogging/ or swimming/ or walking/ or stair climbing/ or physical endurance/ or physical exertion/ or physical fitness/ or cardiorespiratory fitness/ 176916

18 Dance Therapy/ 445

19 exercise therapy/ or (exercise* adj4 therap*).mp. 57589

20 (physical* activ* or physical* exert* or fitness or aerobic* or workout* or interval training or (endurance adj2 (train* or physical))).mp. 414298

21 (exercis* adj4 (exert* or endurance or physical or vigor* or intense or high-intensity or medium-intensity or moderate-intensity or variable-intensity or ((vary* or chang*) adj2 intensit*))).mp. 52300

22 ((treadmill* not treadmill test) or (cardio* adj3 (class* or exercis* or train* or machine*)) or ergometer* or ergometre*).mp. 63553

23 (walking or ((walk or walks) adj3 (fast or brisk* or quickly or regular)) or swim* or running or jogging or cycling or bicycl* or bike or biking or spinning or spin class* or spin bike* or kickbox* or boxing or x-country ski* or ((stair* adj3 climb*) not stair climb test)).mp. 359157

24 (rowing or skating or hiking or danc* or calisthenic* or zumba or yoga or pilates).mp. 21865

25 (pedomet* or acceleromet* or Step-count* or count*-step* or Fitness-tracker*).mp. 28473

26 (exercise adj3 (intervention* or program*)).mp. 31545

27 17 or 18 or 19 or 20 or 21 or 22 or 23 or 24 or 25 or 26 832125

28 exp Clinical trial/ or randomized.tw. or placebo.tw. or randomly.tw. or trial.tw. or groups.tw. 3869803

29 16 and 27 and 28 1565

30 limit 29 to animals 49

31 29 not 30 1516

32 31 not (rat or rats or dog or dogs or rabbit* or mouse or mice or animal model).ti. 1512

33 limit 32 to "all child (0 to 18 years)" 268

34 limit 33 to "all adult (19 plus years)" 200

35 32 not (33 not 34) 1444

36 ((child* or infant* or neonate* or toddler* or pediatric* or paediatric* or teen* or adolescen*) not adult*).ti. 1393925

37 (footstrike* or foot strike* or foot landing or footwear or foot load* or load* respons* or loading rates or jump performance or knee flexion or spine loading or contact forces or recovery kinetics or functional-movement or playing surface or biomechanic* or muscle activation or physical function test*).ti. 32715

38 ((cross-sectional or cohort or qualitative or case series or case study or case report) not (trial or rct)).mp. 2231854

39 (study protocol or (protocol adj4 (trial or randomized-controlled-trial or review))).ti. 28515

40 35 not (36 or 37 or 38 or 39) 1243

41 (exercise* adj8 (control or group)).ab. 39776

42 (Exercise* or activity or active or physical* exert* or fit or fitness or walking or retrowalking or ((walk or walks) adj3 (fast or brisk* or quickly or regular)) or swim* or jogging or bicycl* or bike or biking or spinning or spin class* or spin bike* or kickbox* or boxing or x-country ski* or treadmill* or (cardio* adj3 (class* or train* or machine*)) or ergometer* or ergometre* or pedomet* or acceleromet* or step-count* or count*-step* or fitness-tracker* or dance or dancing or running or cycling or climbing or aerobic* or endurance or workout* or aquacise or sports or training or Zumba or rowing or skating or hiking or yoga or Pilates or hiit).ti,kf. 1304292

43 40 and (41 or 42) 778

**Embase <1974 to 2023 April 13> (OVID Interface)**

Date searched: April 14, 2023

Results: 1035

1 temporomandibular joint disorder/ 16041

2 exp neck injury/ or "head and neck injury"/ or exp ear injury/ or exp maxillofacial injury/ or cervical disk hernia/ 98269

3 facial pain/ or exp neck pain/ 38340

4 (intervertebral disc degeneration/ or intervertebral disc hernia/ or discogenic pain/) and (exp neck/ or (neck or cervical or cervicothoracic or cervicogenic or craniocervical).mp.) 5600

5 (exp Temporomandibular Joint/ or exp Masticatory Muscles/ or ((Masticatory or Masseter or pterygoid or Temporal) adj2 Muscl*).mp.) and (pain* or ache* or discomfort or sore*).mp. 7899

6 (myofascial pain/ or (myofascial adj2 pain).mp.) and (face or facial or orofacial or craniofacial or jaw or head or neck or mandib* or craniomandibular).mp. 3824

7 ((Pain* or ache* or discomfort* or injur* or sore* or tender* or agony or agonies or excruciat* or tear or tears or injur* or sprain* or strain* or contracture* or dislocation* or cramp* or impingement or instabilit*) adj8 ((head not head injur*) or cranial or facial or orofacial or face or jaw or mandibular or craniomandibular or tmj or temporomandibular joint or neck or cervical or craniocervical or cervicogenic or cervicothoracic or Cx)).mp. 170853

8 (chronic pain or acute pain or subacute pain or intractable pain or musculoskeletal pain or noncancer pain or non-cancer pain or non-malignant pain or nonmalignant pain or nonneoplastic pain or non-neoplastic pain or myofascial trigger point*).mp. and (exp neck/ or exp head/) 2436

9 (neckache* or whiplash or (neck adj4 complaint*) or Orofacial migraine or burning mouth syndrome or cervicodynia or cervicalgia or ((temporomandibular or craniomandibular) adj4 (disorder* or disease* or syndrome* or pain*)) or tmj or tmd or costen* or ((neck or cervical or cervicothoracic or cervicogenic or craniocervical) and (radiculopathy or zygapophyseal joint syndrome or facet joint syndrome or Intervertebral Disc Degeneration or Intervertebral Disc Displacement or disc hernia* or herniated disc*))).mp. 46328

10 (neck disability index or Copenhagen Neck functional disability scale).mp. 4825

11 or/1-10 278619

12 exp spinal cord injury/ 90045

13 exp *osteoporosis/ 70903

14 exp *neoplasm/ 4072992

15 exp *experimental arthritis/ 4505

16 exp *rheumatoid arthritis/ 145364

17 exp *psoriatic arthritis/ 15905

18 exp *rheumatic fever/ 4345

19 *sacroiliitis/ 1910

20 *ankylosing spondylitis/ or *spondylarthritis/ 24047

21 *lupus vulgaris/ 1138

22 *chronic obstructive lung disease/ 82348

23 (osteoporo* or RA or arthritis or ankylosing spondylitis or SCI or spinal cord* or spin* injur* or parapleg* or quadripleg* or lupus or (cancer* not non-cancer) or (malignan* not non-malignan*) or oncolog* or neoplasm* or tumour* or tumor* or burn or burns or abuse* or donor or copd or chronic obstructive pulmonary disease or stroke).ti. 3440577

24 or/12-23 5650468

25 physical activity/ or climbing/ or cycling/ or jogging/ or jumping/ or running/ or swimming/ 297292

26 exp climbing/ 8279

27 walking/ 83504

28 aerobic exercise/ or aquatic exercise/ or circuit training/ or endurance training/ or exercise intensity/ or high intensity interval training/ or pilates/ 45725

29 circuit training/ 393

30 cardiorespiratory fitness/ 8727

31 dance therapy/ 717

32 kinesiotherapy/ or dynamic exercise/ 39897

33 ((exercise* adj4 therap*) or physical* activ* or physical* exert* or fitness or aerobic* or workout* or interval training or (endurance adj2 (train* or physical)) or (treadmill* not treadmill test) or (cardio* adj3 (class* or exercis* or train* or machine*)) or ergometer* or ergometre* or walking or ((walk or walks) adj3 (fast or brisk* or quickly or regular)) or swim* or running or jogging or cycling or bicycl* or bike or biking or spinning or spin class* or spin bike* or kickbox* or boxing or x-country ski* or yoga or zumba or pilates or ((stair* adj3 climb*) not stair climb test) or rowing or skating or hiking or danc* or pedomet* or acceleromet* or Step-count* or count*-step* or Fitness-tracker*).mp. 1053198

34 (exercis* adj4 (exert* or endurance or physical or vigor* or intense or high-intensity or medium-intensity or moderate-intensity or variable-intensity or ((vary* or chang*) adj2 intensit*))).mp. 72048

35 (exercise adj3 (intervention* or program*)).mp. 44990

36 or/25-35 1116025

37 exp clinical trial/ or randomized.tw. or placebo.tw. or randomly.tw. or trial.tw. or groups.tw. 5794556

38 (11 not 24) and 36 and 37 2515

39 limit 38 to (animals and animal studies) 96

40 38 not 39 2419

41 40 not (rat or rats or dog or dogs or rabbit* or mouse or mice or animal model).ti. 2403

42 limit 41 to child <unspecified age> 172

43 limit 42 to (adult <18 to 64 years> or aged <65+ years>) 80

44 41 not (42 not 43) 2311

45 44 not ((child* or infant* or neonate* or toddler* or pediatric* or paediatric* or teen* or adolescen*) not adult*).ti. 2264

46 (footstrike* or foot strike* or foot landing or footwear or foot load* or load* respons* or loading rates or jump performance or knee flexion or spine loading or contact forces or recovery kinetics or functional-movement or playing surface or biomechanic* or muscle activation or physical function test*).ti. 38791

47 ((cross-sectional or cohort or qualitative or case series or case study or case report) not (trial or rct)).mp. 5460309

48 (study protocol or (protocol adj4 (trial or randomized-controlled-trial or review))).ti. 30346

49 45 not (46 or 47 or 48) 1973

50 (exercise* adj8 (control or group)).ab. 56217

51 (Exercise* or activity or active or physical* exert* or fit or fitness or walking or retrowalking or ((walk or walks) adj3 (fast or brisk* or quickly or regular)) or swim* or jogging or bicycl* or bike or biking or spinning or spin class* or spin bike* or kickbox* or boxing or x-country ski* or treadmill* or (cardio* adj3 (class* or train* or machine*)) or ergometer* or ergometre* or pedomet* or acceleromet* or step-count* or count*-step* or fitness-tracker* or dance or dancing or running or cycling or climbing or aerobic* or endurance or workout* or aquacise or sports or training or Zumba or rowing or skating or hiking or yoga or Pilates or hiit).ti,kf. 1605118

52 49 and (51 or 50) 1035

**CINAHL Plus with Full Text (EBSCOhost Interface)**

Date searched: April 14, 2023

Results: 528

S1 (MH "Craniomandibular Disorders+") OR (MH "Neck Injuries+") OR (MH "Facial Pain") OR (MH "Neck Pain") OR ((Pain* or ache* or discomfort* or injur* or sore* or tender* or agony or agonies or excruciat* or tear or tears or injur* or sprain* or strain* or contracture* or dislocation* or cramp* or impingement or instabilit*) N8 ((head not head-injur*) or cranial or facial or orofacial or face or jaw or mandibular or craniomandibular or tmj or temporomandibular-joint or neck or cervical or craniocervical or cervicogenic or cervicothoracic or Cx)) or (((myofascial N2 pain) or chronic-pain or acute-pain or subacute-pain or intractable-pain or musculoskeletal-pain or noncancer-pain or non-cancer-pain or non-malignant-pain or nonmalignant-pain or nonneoplastic-pain or non-neoplastic-pain or myofascial-trigger-point*) and (face or facial or orofacial or craniofacial or jaw or head or neck or mandib* or craniomandibular)) or neckache* or whiplash or (neck N4 complaint*) or orofacial-migraine or burning-mouth syndrome or cervicodynia or cervicalgia or ((temporomandibular or craniomandibular) N4 (disorder* or disease* or syndrome* or pain*)) or tmj or tmd or costen* or ((neck or cervical or cervicothoracic or cervicogenic or craniocervical) and (radiculopathy or zygapophyseal-joint-syndrome or facet-joint-syndrome or intervertebral-disc-degeneration or intervertebral-disc-displacement or disc-hernia* or herniated-disc*)) or (((Masticatory or Masseter or pterygoid or Temporal) N2 Muscl*) and (pain* or ache* or discomfort or sore*)) or neck-disability-index or Copenhagen-Neck-functional-disability-scale

S2 (MM "Arthritis") OR (MM "Arthritis, Infectious+") OR (MM "Arthritis, Psoriatic") OR (MM "Arthritis, Rheumatoid+") OR (MM "Rheumatic Fever") OR (MM "Spondylarthritis+") OR (MM "Neoplasms+") OR (MM "Osteoporosis+") OR (MM "Lupus Nephritis") OR (MM "Lupus Erythematosus, Systemic+") OR (MM "Lupus Erythematosus, Cutaneous") OR (MM "Pulmonary Disease, Chronic Obstructive+") OR TI(osteoporo* or RA or arthritis or ankylosing-spondylitis or SCI or spinal-cord* or spin*-injur* or parapleg* or quadripleg* or lupus or (cancer* not non-cancer) or (malignan* not non-malignan*) or oncolog* or neoplasm* or tumour* or tumor* or burn or burns or abuse* or donor or copd or chronic-obstructive-pulmonary-disease or stroke)

S3 S1 NOT S2

S4 (MH "Aerobic Exercises+") OR (MH "Cycling") OR (MH "Dance Therapy") OR (MH "Running+") OR (MH "Cross Country Skiing") OR (MH "Cardiorespiratory Fitness") OR (MH "Physical Fitness") OR (MH "Therapeutic Exercise") OR (MH "Conditioning, Cardiopulmonary") OR (MH "High-Intensity Interval Training") OR (MH "Pilates") OR ( (exercise* N4 therap*) OR (exercise N3 (intervention* or program*)) OR physical*-activ* or physical*-exert* or fitness or aerobic* or workout* or interval-training or (endurance N2 (train* or physical)) OR (exercis* N4 (exert* or endurance or physical or vigor* or intense or high-intensity or medium-intensity or moderate-intensity or variable-intensity or ((vary* or chang*) N2 intensit*))) OR (treadmill* not treadmill-test) or (cardio* N3 (class* or exercis* or train* or machine*)) or ergometer* or ergometre* or walking or ((walk or walks) N3 (fast or brisk* or quickly or regular)) or swim* or running or jogging or cycling or bicycl* or bike or biking or spinning or spin-class* or spin-bike* or kickbox* or boxing or x-country-ski* or ((stair* N3 climb*) not stair-climb-test) or rowing or skating or hiking or danc* or calisthenic* or zumba or yoga or pilates or pedomet* or acceleromet* or Step-count* or count*-step* or Fitness-tracker* )

S5 (MH "Clinical trial+") or randomized or placebo or randomly or "trial" or "groups"

S6 S3 AND S4 AND S5

S7 S6 NOT TI(rat or rats or dog or dogs or rabbit* or mouse or mice or animal model)

S8 S7 NOT ((MH "Child+") NOT (MH "Adult+"))

S9 S8 NOT (TI((child* or infant* or neonate* or toddler* or pediatric* or paediatric* or teen* or adolescen*) not adult*) OR TI(study-protocol) OR TI(protocol N4 (trial or randomi*-controlled-trial or review)) OR TI(footstrike* or foot-strike* or foot-landing or footwear or foot-load* or load*-respons* or loading-rates or jump-performance or knee-flexion or spine-loading or contact-forces or recovery-kinetics or functional-movement or playing-surface or biomechanic* or muscle-activation or physical-function-test*) OR ((cross-sectional or cohort or qualitative or case-series or "case-study" or "case report") not (trial or rct)))

S10 (exercise* N8 (control or group)) OR TI(Exercise* or activity or active or physical*-exert* or fit or fitness or walking or retrowalking or ((walk or walks) N3 (fast or brisk* or quickly or regular)) or swim* or jogging or bicycl* or bike or biking or spinning or spin-class* or spin-bike* or kickbox* or boxing or x-country-ski* or treadmill* or (cardio* N3 (class* or train* or machine*)) or ergometer* or ergometre* or pedomet* or acceleromet* or step-count* or count*-step* or fitness-tracker* or dance or dancing or running or cycling or climbing or aerobic* or endurance or workout* or aquacise or sports or training or Zumba or rowing or skating or hiking or yoga or Pilates or hiit)

S11 S9 AND S10

**Cochrane Library (Trials database only) (Wiley Interface)**

Date searched: April 14, 2023

Results: 1290

#1 [mh "craniomandibular disorders"] OR [mh "temporomandibular joint disorders"] or [mh ^"neck injuries"] OR [mh ^"whiplash injuries"] or [mh ^"facial pain"] OR [mh ^"neck pain"]

#2 (((Pain* or ache* or discomfort* or injur* or sore* or tender* or agony or agonies or excruciat* or tear or tears or injur* or sprain* or strain* or contracture* or dislocation* or cramp* or impingement or instabilit*) NEAR/8 ((head not head-injury) or cranial or facial or orofacial or face or jaw or mandibular or craniomandibular or tmj or temporomandibular-joint or neck or cervical or craniocervical or cervicogenic or cervicothoracic or Cx)) or (((myofascial NEAR/2 pain) or chronic-pain or acute-pain or subacute-pain or intractable-pain or musculoskeletal-pain or noncancer-pain or non-cancer-pain or non-malignant-pain or nonmalignant-pain or nonneoplastic-pain or non-neoplastic-pain or myofascial-trigger-point) and (face or facial or orofacial or craniofacial or jaw or head or neck or mandib* or craniomandibular)) or neckache* or whiplash or (neck NEAR/4 complaint*) or orofacial-migraine or burning-mouth-syndrome or cervicodynia or cervicalgia or ((temporomandibular or craniomandibular) NEAR/4 (disorder* or disease* or syndrome* or pain*)) or tmj or tmd or costen* or ((neck or cervical or cervicothoracic or cervicogenic or craniocervical) and (radiculopathy or zygapophyseal-joint-syndrome or facet-joint-syndrome or intervertebral-disc-degeneration or intervertebral-disc-displacement or (disc NEXT hernia*) or (herniated NEXT disc*))) or (((Masticatory or Masseter or pterygoid or Temporal) NEAR/2 Muscl*) and (pain* or ache* or discomfort or sore*)) or neck-disability-index or Copenhagen-Neck-functional-disability-scale):ti,ab,kw

#3 (osteoporo* or RA or arthritis or ankylosing-spondylitis or SCI or spinal-cord or (spin* NEXT injur*) or parapleg* or quadripleg* or lupus or (cancer* not non-cancer) or (malignan* not non-malignant) or oncolog* or neoplasm* or tumour* or tumor* or burn or burns or abuse* or donor or copd or chronic-obstructive-pulmonary-disease or stroke):ti

#4 [mh "Spinal Cord Injuries"[mj]] or [mh "Osteoporosis"[mj]] or [mh "Neoplasms"[mj]] or [mh ^"arthritis, experimental"[mj]] or [mh ^"arthritis, infectious"[mj]] or [mh ^"arthritis, juvenile"[mj]] or [mh ^"arthritis, psoriatic"[mj]] or [mh "arthritis, rheumatoid"[mj]] or [mh "rheumatic fever"[mj]] or [mh ^"sacroiliitis"[mj]] or [mh "spondylarthritis"[mj]] OR [mh "Lupus Erythematosus, Discoid"[mj]] or [mh "Lupus Vasculitis, Central Nervous System"[mj]] or [mh "Lupus Erythematosus, Cutaneous"[mj]] or [mh "Lupus Nephritis"[mj]] or [mh "Lupus Erythematosus, Systemic"[mj]] OR [mh "Lung Diseases, Obstructive"[mj]]

#5 (#1 OR #2) NOT (#3 OR #4)

#6 [mh ^"physical conditioning, human"] or [mh ^"circuit-based exercise"] or [mh ^"endurance training"] or [mh ^"high-intensity interval training"] or [mh ^"running"] or [mh ^"jogging"] or [mh ^"swimming"] or [mh ^"walking"] or [mh ^"stair climbing"] or [mh ^"physical endurance"] or [mh ^"physical exertion"] or [mh ^"physical fitness"] or [mh ^"cardiorespiratory fitness"] or [mh ^"Dance Therapy"] or [mh ^"exercise therapy"]

#7 (exercise* NEAR/4 therap*):ti,ab,kw OR (exercise NEAR/3 (intervention* or program*)):ti,ab,kw OR ((physical* NEXT activ*) or (physical* NEXT exert*) or fitness or aerobic* or workout* or interval-training or (endurance NEAR/2 (train* or physical))):ti,ab,kw OR (exercis* NEAR/4 (exert* or endurance or physical or vigor* or "intense" or high-intensity or medium-intensity or moderate-intensity or variable-intensity or ((vary* or chang*) NEAR/2 intensit*))):ti,ab,kw OR ((treadmill* not treadmill-test) or (cardio* NEAR/3 (class* or exercis* or train* or machine*)) or ergometer* or ergometre*):ti,ab,kw OR (walking or ((walk or walks) NEAR/3 (fast or brisk* or quickly or regular)) or swim* or running or jogging or cycling or bicycl* or bike or biking or spinning or spin-class or spin-bike or kickbox* or boxing or (x-country NEXT ski*) or ((stair* NEAR/3 climb*) not stair-climb-test)):ti,ab,kw OR (rowing or skating or hiking or danc* or calisthenic* or zumba or yoga or pilates):ti,ab,kw OR (pedomet* or acceleromet* or (Step* NEXT count*) or Fitness-tracker):ti,ab,kw

#8 #5 AND (#6 OR #7)

#9 ((child* or infant* or neonate* or toddler* or pediatric* or paediatric* or teen* or adolescen*) not adult*):ti OR (footstrike* or foot-strike or foot-landing or footwear or foot-load or (load* NEXT respons*) or loading-rates or jump-performance or knee-flexion or spine-loading or contact-forces or recovery-kinetics or functional-movement or playing-surface or biomechanic* or muscle-activation or physical-function-test*):ti OR (study-protocol or (protocol NEAR/3 (trial or randomized-controlled-trial or review))):ti

#10 (exercise* NEAR/8 (control or group)):ab OR (Exercise* or activity or active or (physical* NEXT exert*) or fit or fitness or walking or retrowalking or ((walk or walks) NEAR/3 (fast or brisk* or quickly or regular)) or swim* or jogging or bicycl* or bike or biking or spinning or spin-class or spin-bike or kickbox* or boxing or (x-country NEXT ski*) or treadmill* or (cardio* NEAR/3 (class* or train* or machine*)) or ergometer* or ergometre* or pedomet* or acceleromet* or (step* NEXT count*) or fitness-tracker or dance or dancing or running or cycling or climbing or aerobic* or endurance or workout* or aquacise or sports or training or Zumba or rowing or skating or hiking or yoga or Pilates):ti

#11 (#8 NOT #9) AND #10

**SCOPUS**

Date searched: Apr 14, 2023

Results: 1038

( ( TITLE-ABS-KEY ( ( ( pain* OR ache* OR discomfort* OR injur* OR sore* OR tender* OR agony OR agonies OR excruciat* OR tear OR tears OR injur* OR sprain* OR strain* OR contracture* OR dislocation* OR cramp* OR impingement OR instabilit* ) W/8 ( ( head AND NOT head-injury ) OR cranial OR facial OR orofacial OR face OR jaw OR mandibular OR craniomandibular OR tmj OR temporomandibular-joint OR neck OR cervical OR craniocervical OR cervicogenic OR cervicothoracic OR cx ) ) OR ( ( ( myofascial W/2 pain ) OR chronic-pain OR acute-pain OR subacute-pain OR intractable-pain OR musculoskeletal-pain OR noncancer-pain OR non-cancer-pain OR non-malignant-pain OR nonmalignant-pain OR nonneoplastic-pain OR non-neoplastic-pain OR myofascial-trigger-point ) AND ( face OR facial OR orofacial OR craniofacial OR jaw OR head OR neck OR mandib* OR craniomandibular ) ) OR neckache* OR whiplash OR ( neck W/4 complaint* ) OR orofacial-migraine OR burning-mouth-syndrome OR cervicodynia OR cervicalgia OR ( ( temporomandibular OR craniomandibular ) W/4 ( disorder* OR disease* OR syndrome* OR pain* ) ) OR tmj OR tmd OR costen* OR ( ( neck OR cervical OR cervicothoracic OR cervicogenic OR craniocervical ) AND ( radiculopathy OR zygapophyseal-joint-syndrome OR facet-joint-syndrome OR intervertebral-disc-degeneration OR intervertebral-disc-displacement OR disc-hernia* OR herniated-disc* ) ) OR ( ( ( masticatory OR masseter OR pterygoid OR temporal ) W/2 muscl* ) AND ( pain* OR ache* OR discomfort OR sore* ) ) OR neck-disability-index OR copenhagen-neck-functional-disability-scale ) AND NOT ( KEY ( arthritis OR "Rheumatic Fever" OR spondylarthritis OR neoplasm* OR osteoporosis OR lupus OR ( pulmonary-disease AND chronic-obstructive ) ) OR TITLE ( osteoporo* OR ra OR arthritis OR ankylosing-spondylitis OR sci OR spinal-cord* OR spin*-injur* OR parapleg* OR quadripleg* OR lupus OR ( cancer* AND NOT non-cancer ) OR ( malignan* AND NOT non-malignan* ) OR oncolog* OR neoplasm* OR tumour* OR tumor* OR burn OR burns OR abuse* OR donor OR copd OR chronic-obstructive-pulmonary-disease OR stroke ) ) ) AND ( KEY ( cross-country-skiing OR cardiorespiratory-fitness OR therapeutic-exercise OR ( conditioning W/1 cardiopulmonary ) OR high-intensity-interval-training ) OR TITLE-ABS-KEY ( ( exercise* W/4 therap* ) OR ( exercise W/3 ( intervention* OR program* ) ) OR physical*-activ* OR physical*-exert* OR fitness OR aerobic* OR workout* OR interval-training OR ( endurance W/2 ( train* OR physical ) ) OR ( exercis* W/4 ( exert* OR endurance OR physical OR vigor* OR intense OR high-intensity OR medium-intensity OR moderate-intensity OR variable-intensity OR ( ( vary* OR chang* ) W/2 intensit* ) ) ) OR ( treadmill* AND NOT treadmill-test ) OR ( cardio* W/3 ( class* OR exercis* OR train* OR machine* ) ) OR ergometer* OR ergometre* OR walking OR ( ( walk OR walks ) W/3 ( fast OR brisk* OR quickly OR regular ) ) OR swim* OR running OR jogging OR cycling OR bicycl* OR bike OR biking OR spinning OR spin-class* OR spin-bike* OR kickbox* OR boxing OR x-country-ski* OR ( ( stair* W/3 climb* ) AND NOT stair-climb-test ) OR rowing OR skating OR hiking OR danc* OR calisthenic* OR zumba OR yoga OR pilates OR pedomet* OR acceleromet* OR step-count* OR count*-step* OR fitness-tracker* ) ) AND ( TITLE-ABS-KEY ( {Clinical-trial} OR {controlled-trial} OR randomi* OR randomly OR ( random W/4 ( allocat* OR distribut* OR assign* ) ) OR {placebo} OR {trial} OR {groups} OR {subgroups} ) OR TITLE ( rct ) ) AND ( ABS ( exercise* W/8 ( control OR group ) ) OR TITLE ( exercise* OR activity OR active OR physical*-exert* OR fit OR fitness OR walking OR retrowalking OR ( ( walk OR walks ) W/3 ( fast OR brisk* OR quickly OR regular ) ) OR swim* OR jogging OR bicycl* OR bike OR biking OR spinning OR spin-class* OR spin-bike* OR kickbox* OR boxing OR x-country-ski* OR treadmill* OR ( cardio* W/3 ( class* OR train* OR machine* ) ) OR ergometer* OR ergometre* OR pedomet* OR acceleromet* OR step-count* OR count*-step* OR fitness-tracker* OR dance OR dancing OR running OR cycling OR climbing OR aerobic* OR endurance OR workout* OR aquacise OR sports OR training OR zumba OR rowing OR skating OR hiking OR yoga OR pilates OR hiit ) ) ) AND NOT ( KEY ( ( child OR adolescen* ) AND NOT adult ) OR TITLE ( ( child* OR infant* OR neonate* OR toddler* OR pediatric* OR paediatric* OR teen* OR adolescen* ) AND NOT adult* ) OR TITLE ( rat OR rats OR dog OR dogs OR rabbit* OR mouse OR mice OR animal-model ) OR TITLE ( study-protocol ) OR TITLE ( protocol W/4 ( trial OR randomi*-controlled-trial OR review ) ) OR TITLE ( footstrike* OR foot-strike* OR foot-landing OR footwear OR foot-load* OR load*-respons* OR loading-rates OR jump-performance OR knee-flexion OR spine-loading OR contact-forces OR recovery-kinetics OR functional-movement OR playing-surface OR biomechanic* OR muscle-activation OR physical-function-test* ) OR TITLE-ABS-KEY ( ( cross-sectional OR cohort OR qualitative OR case-series OR {case-study} OR {case report} ) AND NOT ( {trial} OR rct ) ) )

**Appendix 2 – Diagnosis description**

**Condition being studied**

The Neck Pain Task Force classification according to the severity of pain describes four grades of neck pain (NP) (Table A2). According to the inclusion criteria, this review considered the first three grades of the classification.

***Table A2. The Neck Pain Task Force: Classification of Neck Pain***

| Grade I | **Neck pain with no signs or symptoms of major structural pathology and no or minor interference with activities of daily living.**  Complaints of NP may be associated with stiffness or tenderness (i.e., Trapezius myalgia), but no significant neurologic complaints. No symptoms or signs suggest major structural pathologies, such as vertebral fracture, dislocation, injury to the spinal cord or nerves, infection, neoplasm, or systemic disease including inflammatory arthropathies. |
| --- | --- |
| Grade II | **Neck pain with no signs or symptoms of major structural pathology but major interference with activities of daily living.**  Complaints of NP are associated with interference in daily activities, but no signs or symptoms to seriously suggest major structural pathology or significant nerve root compression. Interference with daily activities can be ascertained by self-report questionnaires. |
| Grade III | **Neck pain with no signs or symptoms of major structural pathology but with neurologic signs of nerve compression.**  Complaints of NP are associated with significant neurologic signs such as decreased deep tendon reflexes, weakness, and/or sensory deficits. These suggest malfunction of spinal nerves or the spinal cord. The more presence of pain or numbness in the upper limb without definitive neurologic findings and consistent imaging studies does not warrant a grade III NP designation. |
| Grade IV | **Neck pain with signs of major structural pathology**  Complaints of NP and/or its associated disorders where the examining clinician detects signs or symptoms suggestive of major structural pathology. Major structural pathologies include, but are not limited to, fractures, spinal cord injuries, infections, neoplasms, or systemic diseases. |

NP: Neck pain

**Appendix 3 – List of excluded**

**Table A3** Presentation of excluded studies with reasons for exclusion.

| **Title** | **Authors** | | **Published Year** | **Journal** | **Reason to exclude** |
| --- | --- | --- | --- | --- | --- |
| Critically appraised paper: Stress inoculation training plus exercise improved disability more than exercise alone for patients with acute whiplash-associated disorder. | Ãiestad, Britt Elin | | 2020 | Journal of Physiotherapy (Elsevier) | Not RCT |
| Effect of ultrasound combined with conventional therapy on neck pain, function, and disability in patients with cervical spondylosis: A randomized placebo-controlled trial | Abdel-Aziem A.A.; Draz A.H.; Battecha K.H.; Mosaad D.M. | | 2014 | Journal of Musculoskeletal Pain | Not aerobic exercise |
| Assessment of interleukin-6 in young swimmers suffering from myofascial pain syndrome using lidocaine phonophoresis: A randomized controlled trial | Abo-El-roos M.A.-M.; El-Negmy E.H.; El-Shemy S.; Sallam A.A.-H. | | 2020 | International Journal of Research in Pharmaceutical Sciences | Not musculoskeletal pain |
| Effects of different physiotherapy applications on pain and mobility of connective tissue in patients with myofascial pain syndrome. | Acar, Basak; Yilmaz, Oznur Tunca | | 2012 | Journal of back and musculoskeletal rehabilitation | Not neck pain |
| The effects of equipment Pilates and yoga as adjuncts to home-based exercises for chronic non-specific neck pain | ACTRN12614000841673, | | 2014 |  | Not aerobic exercise |
| Electroanalgesia in the therapeutic approach of the inspecific chronic neck pain in Primary Care. Randomised controlled trial | ACTRN12616000964415, | | 2016 |  | Clinical trial registration / Trial no finished |
| WalkBack - Effectiveness and cost-effectiveness of a progressive individualised walking and education program for the prevention of a recurrence of low back pain | ACTRN12619001134112, | | 2019 |  | Clinical trial registration / Trial no finished |
| The impact of therapeutic exercise on pain, quality of life and musical performance in instrumentalist musicians. Randomised Clinical trial | ACTRN12621001398897, | | 2021 |  | Not aerobic exercise |
| Effect of Exercise on Individuals with Chronic Neck Pain and Central Sensitisation | ACTRN12622000642785, | | 2022 |  | Clinical trial registration / Trial no finished |
| Comparison of the Effects of Cervical Thrust Manipulation and Exercise in Mechanical Neck Pain: A Randomized Controlled Trial. | Akguller, Tugba; Coskun, Resat; Analay Akbaba, Yildiz | | 2023 | Physiotherapy theory and practice | Not aerobic exercise |
| Efficacy of neck stabilization and Pilates exercises on pain, sleep disturbance and kinesiophobia in patients with non-specific chronic neck pain: A randomized controlled trial | Akodu, A.K.; Nwanne, C.A.; Fapojuwo, O.A. | | 2021 | Journal of Bodywork and Movement Therapies | Not isolate aerobic exercise |
| Investigation of the effectiveness of muscle inhibition and space correction techniques of kinesiotaping method in female patients with myofascial pain syndrome related to upper trapezius active trigger points | Akpinar M.F.; Ketenci A.; Sindel D. | | 2015 | Annals of the Rheumatic Diseases | Not aerobic exercise |
| Investigation of Effectiveness of Two Different Kinesiotaping Techniques in Myofascial Pain Syndrome: An Open-Label Randomized Clinical Trial. | Akpinar, Fatma Merih; Sindel, Dilsad; Ketenci, Aysegul | | 2021 | Pain physician | Not aerobic exercise |
| Evaluation of a program to reduce back pain in nursing personnel. | Alexandre, N M; de Moraes, M A; Correa Filho, H R; Jorge, S A | | 2001 | Revista de saude publica | Not neck pain |
| Effect of yoga on chronic non-specific neck pain: an unconditional growth model | Allende, S; Anandan, A; Lauche, R; Cramer, H | | 2017 |  | Not RCT |
| Effect of yoga on chronic non-specific neck pain: An unconditional growth model. | Allende, Santiago; Anandan, Anita; Lauche, Romy; Cramer, Holger | | 2018 | Complementary therapies in medicine | Not RCT |
| A randomized controlled intervention trial to relieve and prevent neck/shoulder pain. | Andersen, Lars L; Jorgensen, Marie B; Blangsted, Anne Katrine; Pedersen, Mogens T; Hansen, Ernst A; Sjogaard, Gisela | | 2008 | Medicine and science in sports and exercise | Not aerobic exercise |
| Effect of physical exercise interventions on musculoskeletal pain in all body regions among office workers: a one-year randomized controlled trial. | Andersen, Lars L; Christensen, Karl Bang; Holtermann, Andreas; Poulsen, Otto M; Sjogaard, Gisela; Pedersen, Mogens T; Hansen, Ernst A | | 2010 | Manual therapy | Not isolate aerobic exercise |
| Influence of psychosocial work environment on adherence to workplace exercise. | Andersen, Lars L | | 2011 | Journal of occupational and environmental medicine | Not RCT |
| Efficacy of 'Tailored Physical Activity' on reducing sickness absence among health care workers: A 3-months randomised controlled trial. | Andersen, Lotte Nygaard; Juul-Kristensen, Birgit; Roessler, Kirsten Kaya; Herborg, Lene Gram; Sorensen, Thomas Lund; Sogaard, Karen | | 2015 | Manual therapy | Not aerobic exercise |
| Efficacy of Tailored Physical Activity or Chronic Pain Self-Management Programme on return to work for sick-listed citizens: A 3-month randomised controlled trial. | Andersen, Lotte Nygaard; Juul-Kristensen, Birgit; Sorensen, Thomas Lund; Herborg, Lene Gram; Roessler, Kirsten Kaya; Sogaard, Karen | | 2015 | Scandinavian journal of public health | Not isolate aerobic exercise |
| LONGER TERM FOLLOW-UP OF THE EFFECTS OF TAILORED PHYSICAL ACTIVITY OR CHRONIC PAIN SELF-MANAGEMENT PROGRAMME ON RETURN-TO-WORK: A RANDOMIZED CONTROLLED TRIAL. | Andersen, Lotte Nygaard; Juul-Kristensen, Birgit; SÃ¸rensen, Thomas Lund; Herborg, Lene Gram; Roessler, Kirsten Kaya; SÃ¸gaard, Karen | | 2016 | Journal of Rehabilitation Medicine (Stiftelsen Rehabiliteringsinformation) | Not neck pain |
| Barriers and Facilitators to Implementing Bundled Acupuncture and Yoga Therapy to Treat Chronic Pain in Community Healthcare Settings: A Feasibility Pilot. | Anderson, Belinda J; Meissner, Paul; Mah, Donna M; Nielsen, Arya; Moonaz, Steffany; McKee, M Diane; Kligler, Benjamin; Milanes, Mirta; Guerra, Hernidia; Teets, Raymond | | 2021 | Journal of alternative and complementary medicine (New York, N.Y.) | Not aerobic exercise |
| Yoga effective for back pain | Anonymous. | | 2006 | Journal of Family Practice | not RCT |
| Erratum: Cost-effectiveness of neck-specific exercise with or without a behavioral approach versus physical activity prescription in the treatment of chronic whiplash-associated disorders: Analyses of a randomized clinical trial: Erratum. | Anonymous | | 2017 | Medicine | Not RCT |
| Effect of aerobic and strength training on pain tolerance, pain appraisal and mood of unfit males as a function of pain location. | Anshel, M H; Russell, K G | | 1994 | Journal of sports sciences | Not musculoskeletal pain |
| Sub occipital myofascial release technique for the treatment of cervicogenic headache | Arab A.M.; Ramezani E. | | 2018 | Journal of Bodywork and Movement Therapies | Not musculoskeletal pain |
| Individually tailored treatment targeting activity, motor behavior, and cognition reduces pain-related disability: a randomized controlled trial in patients with musculoskeletal pain. | Asenlof, Pernilla; Denison, Eva; Lindberg, Per | | 2005 | Journal Pain | Not isolate aerobic exercise |
| The effect of two exercise regimes; motor control versus endurance/strength training for patients with whiplash-associated disorders: a randomized controlled pilot study. | Ask, Tove; Strand, Liv I; Skouen, Jan Sture | | 2009 | Clinical rehabilitation | Whiplash disorders |
| Comparison of effectiveness of Transcutaneous Electrical Nerve Stimulation and Kinesio Taping added to exercises in patients with myofascial pain syndrome. | Azatcam, Gokmen; Atalay, Nilgun Simsir; Akkaya, Nuray; Sahin, Fusun; Aksoy, Sibel; Zincir, Ozge; Topuz, Oya | | 2017 | Journal of back and musculoskeletal rehabilitation | Not aerobic exercise |
| The effect of relaxation exercises for the masticator muscles on temporomandibular joint dysfunction (TMD) | Bae, Y.; Park, Y. | | 2013 | Journal of Physical Therapy Science | Not aerobic exercise |
| Self-Kinematic Training for Flight-Associated Neck Pain: a Randomized Controlled Trial | Bahat, H.S.; German, D.; Palomo, G.; Gold, H.; Nir, Y.F. | | 2020 | Aerospace Medicine and Human Performance | Not aerobic exercise |
| 24-weeks supervised and home-based training program improves motor function in FSHD patients | Bankole L.C.; Millet G.Y.; Temesi J.; Wuyam B.; Bachasson D.; Kadi F.; Antoine J.C.; Feasson L. | | 2014 | Annals of Physical and Rehabilitation Medicine | Not musculoskeletal pain |
| Effect of physical therapy on pain, mental status, and quality of life in patients with cervical myofascial pain syndrome. | Basak Acar; Ã–znur Tunca Yilmaz | | 2012 | Turkish Journal of Physiotherapy Rehabilitation | Conference abstract |
| Manual Therapy, Therapeutic Patient Education, and Therapeutic Exercise, an Effective Multimodal Treatment of Nonspecific Chronic Neck Pain: A Randomized Controlled Trial. | Beltran-Alacreu, Hector; Lopez-de-Uralde-Villanueva, Ibai; Fernandez-Carnero, Josue; La Touche, Roy | | 2015 | American journal of physical medicine & rehabilitation | Not aerobic exercise |
| Does a Web-Based Exercise Programming System Improve Home Exercise Adherence for People With Musculoskeletal Conditions?: A Randomized Controlled Trial. | Bennell, Kim L; Marshall, Charlotte J; Dobson, Fiona; Kasza, Jessica; Lonsdale, Chris; Hinman, Rana S | | 2019 | American journal of physical medicine & rehabilitation | Not aerobic exercise |
| The effectiveness of a work style intervention and a lifestyle physical activity intervention on the recovery from neck and upper limb symptoms in computer workers. | Bernaards, Claire M; Ariens, Geertje A M; Knol, Dirk L; Hildebrandt, Vincent H | | 2007 | Pain | Recommendation |
| The cost-effectiveness of a lifestyle physical activity intervention in addition to a work style intervention on recovery from neck and upper limb symptoms and pain reduction in computer workers. | Bernaards, Claire M; Bosmans, Judith E; Hildebrandt, Vincent H; van Tulder, Maurits W; Heymans, Martijn W | | 2011 | Occupational and environmental medicine | Recommendation |
| Manual therapy versus therapeutic exercise in non-specific chronic neck pain: a randomized controlled trial. | Bernal-Utrera, Carlos; Gonzalez-Gerez, Juan Jose; Anarte-Lazo, Ernesto; Rodriguez-Blanco, Cleofas | | 2020 | Trials | Not aerobic exercise |
| Effect of an exercise programme for the prevention of back and neck pain in poultry slaughterhouse workers | Bertozzi L.; Villafane J.H.; Capra F.; Reci M.; Pillastrini P. | | 2015 | Occupational therapy international | Not aerobic exercise |
| Office-cycling while working: an innovative concept to prevent and reduce musculoskeletal pain in office workers-a controlled feasibility study | Bjorklund, M; Tronarp, R; Granas, M; Dahlgren, G; McDonough, S; Nyberg, A; Hager, C | | 2015 | Physiotherapy | Conference abstract |
| One-year randomized controlled trial with different physical-activity programs to reduce musculoskeletal symptoms in the neck and shoulders among office workers. | Blangsted, Anne Katrine; Sogaard, Karen; Hansen, Ernst A; Hannerz, Harald; Sjogaard, Gisela | | 2008 | Scandinavian journal of work, environment & health | Not isolate aerobic exercise |
| Work-site health promotion of frequent computer users: comparing selected interventions. | Blasche, Gerhard; Pfeffer, Manuela; Thaler, Helga; Gollner, Erwin | | 2013 | Work (Reading, Mass.) | Not aerobic exercise |
| Is deep cervical neck flexors' training more effective than general neck exercises or advice in patients with chronic neck pain? a prospective randomized controlled trial | Bobos P.; Papanikolaou T.; Koutsojannis C.; MacDermid J.; Billis E. | | 2016 | Manual Therapy | Not aerobic exercise |
| Effects of strength and endurance training of superficial and deep neck muscles on muscle activities and pain levels of females with chronic neck pain. | Borisut, Sudarat; Vongsirinavarat, Mantana; Vachalathiti, Roongtiwa; Sakulsriprasert, Prasert | | 2013 | Journal of physical therapy science | Not aerobic exercise |
| Pain education combined with neck- and aerobic training is more effective at relieving chronic neck pain than pain education alone--A preliminary randomized controlled trial. | Brage, K; Ris, I; Falla, D; Sogaard, K; Juul-Kristensen, B | | 2015 | Manual therapy | Not isolate aerobic exercise |
| Effects of yoga, strength training and advice on back pain: a randomized controlled trial. | Bramberg, Elisabeth Bjork; Bergstrom, Gunnar; Jensen, Irene; Hagberg, Jan; Kwak, Lydia | | 2017 | BMC musculoskeletal disorders | Not aerobic exercise |
| Isotonic exercises and relaxing techniques in individuals with temporomandibular dysfunction. | Brandao, Renata De Assis Fonseca Santos; Mendes, Carlos Mauricio Cardeal; Brandao Filho, Rivail Almeida; De Sena, Eduardo Ponde | | 2022 | Cranio : the journal of craniomandibular practice | Not aerobic exercise |
| Dynamic neck muscle training or relaxation does not improve chronic neck pain | Bregeon F. | | 2003 | Australian Journal of Physiotherapy | not RCT |
| OA14.04. A randomized controlled trial of spinal manipulation, medication or home exercise for acute and subacute neck pain. | Bronfort, G.; Evans, R.; Anderson, A.; Svendsen, K.; Bracha, Y.; Grimm, R. | | 2012 | BMC Complementary & Alternative Medicine | Not aerobic exercise |
| A randomized controlled trial of spinal manipulation, medication or home exercise for acute and subacute neck pain | Bronfort, G; Evans, R; Anderson, A; Svendsen, K; Bracha, Y; Grimm, R | | 2012 | Ann Intern Med. | Not aerobic exercise |
| The effectiveness of a supervised physical training model tailored to the individual needs of patients with whiplash-associated disorders--a randomized controlled trial. | Bunketorp, Lina; Lindh, Malin; Carlsson, Jane; Stener-Victorin, Elisabet | | 2006 | Clinical rehabilitation | Not musculoskeletal pain |
| The Effect of Mulligan Mobilization Technique in Older Adults with Neck Pain: A Randomized Controlled, Double-Blind Study. | Buyukturan, Oznur; Buyukturan, Buket; Sas, Senem; Kararti, Caner; Ceylan, Ismail | | 2018 | Pain research & management | Not aerobic exercise |
| Effects of an active intervention based on myofascial release and neurodynamics in patients with chronic neck pain: a randomized controlled trial | Cabrera-Martos I.; Rodriguez-Torres J.; Lopez-Lopez L.; Prados-Roman E.; Granados-Santiago M.; Valenza M.C. | | 2020 | Physiotherapy theory and practice | Not aerobic exercise |
| Effectiveness of mobilisation of the upper cervical region and craniocervical flexor training on orofacial pain, mandibular function and headache in women with TMD. A randomised, controlled trial. | Calixtre, LetÃ­cia B.; Oliveira, Ana Beatriz; de Sena Rosa, Lianna Ramalho; Armijoâ€Olivo, Susan; Visscher, Corine M.; Alburquerqueâ€SendÃ­n, Francisco | | 2019 | Journal of Oral Rehabilitation | Not aerobic exercise |
| Is physical exercise with a support group of elderly effective for chronic musculoskeletal pain? | Carioca A.L.; Arca G.L.; Milanesio M.; Martinez J.E.; Novo N.F. | | 2012 | Journal of Musculoskeletal Pain | Not musculoskeletal pain |
| Effectiveness of pilates method for the treatment of chronic mechanical neck pain | Cazotti L.A.; Jones A.; Silva D.R.; Ribeiro L.H.C.; Natour J. | | 2013 | Arthritis and Rheumatism | Not isolate aerobic exercise |
| Effectiveness of the Pilates method in the treatment of chronic mechanical-postural neck pain | Cazotti L.A.; Jones A.; Ribeiro L.H.C.; Silva D.R.; Natour J. | | 2015 | Annals of the Rheumatic Diseases | Conference abstract |
| Effect of exercise on chronic neck pain and central sensitization: A protocol for a randomized crossover trial. | Chen, Kexun Kenneth; Hutchinson, Mark Rowland; Rolan, Paul; de Zoete, Rutger Marinus Johannes | | 2023 | Experimental physiology | Clinical trial registration / Trial no finished |
| Effectiveness of pilates-based thoraic exercises for patients with neck pain | Cheng F.-H.; Huang J.-R.; Chen W.-Y.; Wang T.-J. | | 2011 | Physiotherapy (United Kingdom) | Not isolate aerobic exercise |
| Effectiveness of Tuina Therapy Combined With Yijinjing Exercise in the Treatment of Nonspecific Chronic Neck Pain: A Randomized Clinical Trial. | Cheng, Zi-Ji; Zhang, Shuai-Pan; Gu, Yuan-Jia; Chen, Zi-Ying; Xie, Fang-Fang; Guan, Chong; Fang, Min; Yao, Fei | | 2022 | JAMA network open | Not aerobic exercise |
| International cooperative study of Taichichuan on rehabilitation of chronic pain in the old patients | ChiCTR2000038682, | | 2020 |  | Conference abstract |
| Effects of 6-week Elastic-band Exercise Program on activation of the deep cervical flexor muscles in Patients with Chronic Neck Pain | ChiCTR-IIR-16008668, | | 2016 |  | Not aerobic exercise |
| The effects of stabilizing exercises on pain and disability of patients with lumbar disc herniation: a randomized controlled trial | ChiCTR-TRC-12002839, | | 2012 |  | Clinical trial registration / Trial no finished |
| Thoracic spine manipulation and exercise versus exercise alone in the management of mechanical neck pain: preliminary analysis of a randomized clinical trial...2010 Combined Sections Meeting (CSM), San Diego, California, February 17-20, 2010 | Cleland J; Childs JD; Glynn PE; Mintken P; Carpenter K; Whitman J | | 2010 | Journal of Orthopaedic & Sports Physical Therapy | Not full text available |
| Randomized-controlled trial comparing yoga and home-based exercise for chronic neck pain. | Cramer, Holger; Lauche, Romy; Hohmann, Claudia; Ludtke, Rainer; Haller, Heidemarie; Michalsen, Andreas; Langhorst, Jost; Dobos, Gustav | | 2013 | The Clinical journal of pain | Not isolate aerobic exercise |
| Yoga for chronic neck pain: a 12-month follow-up. | Cramer, Holger; Lauche, Romy; Hohmann, Claudia; Langhorst, Jost; Dobos, Gustav | | 2013 | Pain medicine (Malden, Mass.) | Not isolate aerobic exercise |
| A clinical trial to study the effects of two exercise programs in patients with neck pain | CTRI/2011/07/001925, | | 2011 |  | Not aerobic exercise |
| A clinical trial to study the effects of two differant exercises,Conventional and Pilates exercise in subjects with Forward head posture having age group 40-60 | CTRI/2020/02/023454, | | 2020 |  | Clinical trial registration / Trial no finished |
| Comparative effect of Pilates and Motor control exercises on non specific neck pain | CTRI/2020/03/024364, | | 2020 |  | Not isolate aerobic exercise |
| EFFECT OF YOGA AND NECK EXERCISES ON DISABILITY, PAIN AND STRENGTH IN CHRONIC NECK PAIN | CTRI/2020/06/026153, | | 2020 |  | Clinical trial registration / Trial no finished |
| Comparative Effect Of Pilates And BrueggerÃ¢??s Exercises Along With Low Level Laser Therapy For Individuals With Forward Head Posture Associated Temporomandibular Joint Dysfunction | CTRI/2021/09/036762, | | 2021 |  | Clinical trial registration / Trial no finished |
| Effect of backward walking on Neck Pain | CTRI/2022/05/042846, | | 2022 |  | Clinical trial registration / Trial no finished |
| Intelligent Physical Exercise Training in a Workplace Setting Improves Muscle Strength and Musculoskeletal Pain: A Randomized Controlled Trial. | Dalager, Tina; Justesen, Just Bendix; Sjogaard, Gisela | | 2017 | BioMed research international | Not aerobic exercise |
| Effectiveness of the Pilates Method in the Treatment of Chronic Mechanical Neck Pain: A Randomized Controlled Trial. | de Araujo Cazotti, Luciana; Jones, Anamaria; Roger-Silva, Diego; Ribeiro, Luiza Helena Coutinho; Natour, Jamil | | 2018 | Archives of physical medicine and rehabilitation | Not isolate aerobic exercise |
| Effect of Bean pillow as an adjunct to exercises for neck pain in cervical spondylosis: A pilot study. | Desai, Krunal V; Rao, Sheela A. | | 2012 | Indian J Physiother Occup Ther | Not aerobic exercise |
| Physiotherapeutic approach in seamstresses with neck pain: A single-blind, randomized clinical trial. | De Toni, Morgana Martins; Duarte, Rafael Silveira; das Neves, Lais Mara Siqueira; Diefenthaeler, Fernando; Fonseca, Marisa de Cassia Registro; Barbosa, Rafael Inacio; Kuriki, Heloyse Uliam; Marcolino, Alexandre Marcio | | 2022 | Journal of bodywork and movement therapies | Not aerobic exercise |
| The effectiveness of aerobic versus strengthening exercise therapy in individuals with chronic whiplash-associated disorder: a randomised single case experimental design study | de Zoete, RMJ; Nikles, J; Coombes, JS; Onghena, P; Sterling, M | | 2022 | Disability and Rehabilitation | Whiplash disorders |
| The immediate effects of modified Yoga positions on musculoskeletal pain relief. | do Rosario, Jose Luis Pimentel; Orcesi, Larissa Schwarzwalder; Kobayashi, Fernanda Naomi; Aun, Alexandre Nicolau; Diolindo Assumpcao, Iane Tavares; Blasioli, Gisele Janaina; Hanada, Erica Sato | | 2013 | Journal of bodywork and movement therapies | Not aerobic exercise |
| Effectiveness of Yoga in patients with chronic neck pain. A randomized controlled trial | DRKS00000454, | | 2011 |  | Not full text available |
| Adhesion Therapy versus exercise therapy in patients with chronic non-specific neck pain, A randomized controlled trial | DRKS00022856, | | 2022 |  | Clinical trial registration / Trial no finished |
| Comparative effectiveness of Pilates and yoga group exercise interventions for chronic mechanical neck pain: quasi-randomised parallel controlled study. | Dunleavy, K; Kava, K; Goldberg, A; Malek, M H; Talley, S A; Tutag-Lehr, V; Hildreth, J | | 2016 | Physiotherapy | Not full text available |
| Aerobic exercises and neck school program for the treatment of patients with chronic neck pain: A randomized single-blind controlled clinical trial | Durmus D.; Kuru O. | | 2013 | Turkiye Fiziksel Tip ve Rehabilitasyon Dergisi | Not isolate aerobic exercise |
| A randomized single-blind controlled clinical trial of phonophoresis for the treatment of chronic neck pain | Durmus D.; Alayli G.; Bilgici A.; Kuru O. | | 2013 | Annals of the Rheumatic Diseases | Not aerobic exercise |
| A randomized placebo-controlled clinical trial of phonophoresis for the treatment of chronic neck pain. | Durmus, Dilek; Alayli, Gamze; Tufekci, Tugce; Kuru, Omer | | 2014 | Rheumatology international | Not aerobic exercise |
| Combination of Exercise and Acupuncture Versus Acupuncture Alone for Treatment of Myofascial Pain Syndrome: A Randomized Clinical Trial. | Eftekharsadat, Bina; Porjafar, Elmira; Eslamian, Fariba; Shakouri, Seyed Kazem; Fadavi, Hamid Reza; Raeissadat, Seyed Ahmad; Babaei-Ghazani, Arash | | 2018 | Journal of acupuncture and meridian studies | Not neck pain |
| Effect of low-impact aerobic exercise combined with music therapy on patients with fibromyalgia. A pilot study. | Espi-Lopez, Gemma V; Ingles, Marta; Ruescas-Nicolau, Maria-Arantzazu; Moreno-Segura, Noemi | | 2016 | Complementary therapies in medicine | Not musculoskeletal pain |
| Supervised exercise with and without spinal manipulation performs similarly and better than home exercise for chronic neck pain: a randomized controlled trial. | Evans, Roni; Bronfort, Gert; Schulz, Craig; Maiers, Michele; Bracha, Yiscah; Svendsen, Kenneth; Grimm, Richard; Garvey, Timothy; Transfeldt, Ensor | | 2012 | Spine | Not aerobic exercise |
| A randomized trial of walking versus physical methods for chronic pain management. | Ferrell, B A; Josephson, K R; Pollan, A M; Loy, S; Ferrell, B R | | 1997 | Aging (Milan, Italy) | Not aerobic exercise |
| Exercise only, exercise with mechanical traction, or exercise with over-door traction for patients with cervical radiculopathy, with or without consideration of status on a previously described subgrouping rule: a randomized clinical trial. | Fritz, Julie M; Thackeray, Anne; Brennan, Gerard P; Childs, John D | | 2014 | The Journal of orthopaedic and sports physical therapy | Not musculoskeletal pain |
| Exercise Only, Exercise With Mechanical Traction, or Exercise With Over-Door Traction for Patients With Cervical Radiculopathy, With or Without Consideration of Status on a Previously Described Subgrouping Rule: a Randomized Clinical Trial | FRITZ, JULIEM; THACKERAY, ANNE; BRENNAN, GERARDP; CHILDS, JOHND | | 2014 | J Orthop Sports Phys Ther | Not musculoskeletal pain |
| Estimating the number needed to treat from continuous outcomes in randomised controlled trials: methodological challenges and worked example using data from the UK Back Pain Exercise and Manipulation (BEAM) trial. | Froud, Robert; Eldridge, Sandra; Lall, Ranjit; Underwood, Martin | | 2009 | BMC medical research methodology | Not aerobic exercise |
| Pain Neuroscience Education and Physical Therapeutic Exercise for Patients with Chronic Spinal Pain in Spanish Physiotherapy Primary Care: A Pragmatic Randomized Controlled Trial. | Galan-Martin, Miguel Angel; Montero-Cuadrado, Federico; Lluch-Girbes, Enrique; Coca-Lopez, Maria Carmen; Mayo-Iscar, Agustin; Cuesta-Vargas, Antonio | | 2020 | Journal of clinical medicine | Not musculoskeletal pain |
| Effectiveness of mobilization therapy and exercises in mechanical neck pain. | Ganesh, G Shankar; Mohanty, Patitapaban; Pattnaik, Monalisa; Mishra, Chittaranjan | | 2015 | Physiotherapy theory and practice | Not aerobic exercise |
| The clinical and EMG assessment of the effects of stabilization exercise on nonspecific chronic neck pain: A randomized controlled trial. | Ghaderi, Fariba; Jafarabadi, Mohammad Asghari; Javanshir, Khodabakhsh | | 2017 | Journal of back and musculoskeletal rehabilitation | Not aerobic exercise |
| Adding Temporomandibular joint treatments to routine physiotherapy for patients with non-specific chronic neck pain: A randomized clinical study. | Ghodrati, Maryam; Mosallanezhad, Zahra; Shati, Mohsen; Noroozi, Mehdi; Moghadam, Afsun Nodehi; Rostami, Mohamad; Nourbakhsh, Mohammad Reza | | 2020 | Journal of bodywork and movement therapies | Not aerobic exercise |
| Pain, disability and adherence to home exercises in patients with chronic neck pain: long term effects of phone surveillance. A randomized controlled study. | Gialanella, Bernardo; Comini, Laura; Olivares, Adriana; Gelmini, Elena; Ubertini, Elena; Grioni, Giuseppe | | 2020 | European journal of physical and rehabilitation medicine | Recommendation |
| Temporomandibular joint repositioning and exercise performance: a double-blind study. | Goldstein, L B; McArdle, W D; Last, F C; Spina, R; Lichtman, S; Meyer, J E; Berger, A I | | 1985 | Cranio : the journal of craniomandibular practice | Not aerobic exercise |
| Long-term evaluation of conservative treatment for myofascial pain-dysfunction syndrome. | Greene, C S; Laskin, D M | | 1974 | Journal of the American Dental Association (1939) | Not RCT |
| Effectiveness of specific neck stabilization exercises or a general neck exercise program for chronic neck disorders: a randomized controlled trial. | Griffiths, Cathrin; Dziedzic, Krysia; Waterfield, Jackie; Sim, Julius | | 2009 | The Journal of rheumatology | Not aerobic exercise |
| Yoga for chronic low back and neck pain in military personnel | Groessl E.; Casteel D.; McKinnon S.; McCarthy A.; Schmalzl L.; Chang D.; Park C. | | 2020 | Global Advances in Health and Medicine | Not isolate aerobic exercise |
| Feasibility of yoga for chronic low back and neck pain in military personnel | Groessl E.; McCarthy A.; Casteel D.; McKinnon S. | | 2021 | Global Advances in Health and Medicine | Conference abstract |
| Comparing Types of Yoga for Chronic Low Back and Neck Pain in Military Personnel: A Feasibility Randomized Controlled Trial. | Groessl, Erik J; Casteel, Danielle; McKinnon, Symone; McCarthy, Adhana; Schmalzl, Laura; Chang, Douglas G; Fowler, Ian M; Park, Crystal L | | 2022 | Global advances in health and medicine | Not aerobic exercise |
| Comparison of three different exercise trainings in patients with chronic neck pain: a randomized controlled study. | Gumuscu, Besta Hazal; Kisa, Eylul Pinar; Kara Kaya, Begum; Muammer, Rasmi | | 2023 | The Korean journal of pain | Not aerobic exercise |
| Short-and long-term effects of exercise on neck muscle function in cervical radiculopathy: A randomized clinical trial | Halvorsen M.; Falla D.; Gizzi L.; Harms-Ringdahl K.; Peolsson A.; Dedering A. | | 2016 | Manual Therapy | Not musculoskeletal pain |
| Influence of neck exercises, combined with either the Chace technique of dance therapy or aerobic training, on pain perception, mood state and cervical range of motion of adults with chronic mechanical neck pain | Har, E | | 2000 | Cochrane Central Register of Controlled Trials | Not RCT |
| Long-term effectiveness of bone-setting, light exercise therapy, and physiotherapy for prolonged back pain: a randomized controlled trial. | Hemmila, Heikki M; Keinanen-Kiukaanniemi, Sirkka M; Levoska, Sinikka; Puska, Pekka | | 2002 | J Manipulative Physiol Ther | Not isolate aerobic exercise |
| No significant differences between intervention programmes on neck, shoulder and low back pain: A prospective randomized study among home-care personnel | Horneij E.; Hemborg B.; Jensen I.; Ekdahl C. | | 2001 | Journal of Rehabilitation Medicine | Not neck pain |
| Manual therapy, physical therapy, or continued care by a general practitioner for patients with neck pain. A randomized, controlled trial. | Hoving, Jan Lucas; Koes, Bart W; de Vet, Henrica C W; van der Windt, Danielle A W M; Assendelft, Willem J J; van Mameren, Henk; Deville, Walter L J M; Pool, Jan J M; Scholten, Rob J P M; Bouter, Lex M | | 2002 | Annals of internal medicine | Not aerobic exercise |
| A 12-words-for-life-nurturing exercise program as an alternative therapy for cervical spondylosis: A randomized controlled trial | Hu Z.; Tang Z.; Wang S.; Ye X.; Wang Y.; Shi Q.; Yang Q.; Cheng S.; Huang M.; Dong Y.; Gu H. | | 2014 | Evidence-based Complementary and Alternative Medicine | Not musculoskeletal pain |
| The comparison of exercise therapy and exercise therapy with posture education effect on neck pain among Tehran general dentists | IRCT2014102919750N1, | | 2015 |  | Not musculoskeletal pain |
| Study of adding aerobic training to acupuncture in treatment of neck myofascial pain syndrome | IRCT201509264641N11, | | 2015 |  | Clinical trial registration / Trial no finished |
| Comparison of the effectiveness of two exercise programs for the rehabilitation of problems due to cervical syndrome. | ÎšÎ±ÏÎ¼Î¯ÏÎ·Ï‚, Î‘ÏÎ¹ÏƒÏ„Î¿Î¼Î­Î½Î·Ï‚; Î“Î¹Î¿Ï†Ï„ÏƒÎ¯Î´Î¿Ï…, Î‘ÏƒÎ·Î¼Î­Î½Î¹Î± | | 2012 | Physiotherapy Issues / Themata Fisikotherapeias | Not aerobic exercise |
| Effects of a home-exercise therapy programme on cervical and lumbar range of motion in nurses | ISRCTN19278735, | | 2015 |  | Not aerobic exercise |
| Resistance training vs general physical exercise in multidisciplinary rehabilitation of chronic neck pain: A randomized controlled trial. | Iversen, Vegard Moe; Vasseljen, Ottar; Mork, Paul Jarle; Fimland, Marius Steiro | | 2018 | Journal of rehabilitation medicine | Not aerobic exercise |
| Physical exercise at the workplace reduces perceived physical exertion during healthcare work: cluster randomized controlled trial. | Jakobsen, Markus Due; Sundstrup, Emil; Brandt, Mikkel; Jay, Kenneth; Aagaard, Per; Andersen, Lars L | | 2015 | Scandinavian journal of public health | Not musculoskeletal pain |
| Factors affecting pain relief in response to physical exercise interventions among healthcare workers. | Jakobsen, M D; Sundstrup, E; Brandt, M; Andersen, L L | | 2017 | Scandinavian journal of medicine & science in sports | Not musculoskeletal pain |
| A cluster-randomized trial of workplace ergonomics and neck-specific exercise versus ergonomics and health promotion for office workers to manage neck pain - a secondary outcome analysis. | Johnston, Venerina; Chen, Xiaoqi; Welch, Alyssa; Sjogaard, Gisela; Comans, Tracy A; McStea, Megan; Straker, Leon; Melloh, Markus; Pereira, Michelle; O'Leary, Shaun | | 2021 | BMC musculoskeletal disorders | Not aerobic exercise |
| Effect of yogic exercises on symptoms of musculoskeletal disorders of upper limbs among computer users: a randomised controlled trial. | Joshi, Vidya S; Bellad, Anjana S | | 2011 | Indian journal of medical sciences | Not isolate aerobic exercise |
| A randomized controlled trial of exercise and manipulative therapy for cervicogenic headache. | Jull, Gwendolen; Trott, Patricia; Potter, Helen; Zito, Guy; Niere, Ken; Shirley, Debra; Emberson, Jonathan; Marschner, Ian; Richardson, Carolyn | | 2002 | Spine | Not aerobic exercise |
| Manipulative therapy and a low load exercise regimen each reduced the frequency and intensity of cervicogenic headache | Jull G.; Trott P.; Potter H. | | 2003 | Evidence-Based Medicine | Not aerobic exercise |
| The effects of different training programs on the trapezius muscle of women with work-related neck and shoulder myalgia | Kadi F.; Ahlgren C.; Waling K.; Sundelin G.; Thornell L.-E. | | 2000 | Acta Neuropathologica | Recommendation |
| Effect of neck stabilization and dynamic exercises on pain, disability and fear avoidance beliefs in patients with non-specific neck pain | Kaka B.; Ogwumike O.O. | | 2015 | Physiotherapy (United Kingdom) | Not aerobic exercise |
| Efficacy of neck stabilisation and dynamic exercises on pain intensity, depression and anxiety among patients with non-specific neck pain | Kaka B.; Ogwumike O.O.; Adeniyi F.A. | | 2016 | Physiotherapy | Not aerobic exercise |
| Effectiveness of neck stabilisation and dynamic exercises on pain intensity, depression and anxiety among patients with non-specific neck pain: a randomised controlled trial. | Kaka, Bashir; Ogwumike, Omoyemi O; Adeniyi, Ade F; Maharaj, Sonill S; Ogunlade, Samuel O; Bello, Bashir | | 2018 | Scandinavian journal of pain | Not aerobic exercise |
| Effectiveness of TENS and home exercises as an adjunct to drug therapy in the management of myogenous masticatory pain: A comparative study | Kandagal Veerabhadrappa, S.; Anbananthan, T.D.; Ying, C.X.; Ramamurthy, P.H.; Yadav, S.; Bin Zamzuri, A.T. | | 2021 | Journal of Oral Medicine and Oral Surgery | Not aerobic exercise |
| Assessment of active exercises in cervical pain syndrome | Karic-Skrijelj, M; Majic, I; Vavra-Hadziahmetovic, N; Skopljak, A; Pasagic, A | | 2008 | Med Arh | Not full text available |
| Effect of respiratory exercise program on non-specific neck pain with upper rib breathing pattern | KCT0006440, | | 2021 |  | Clinical trial registration / Trial no finished |
| Long-term effect of direction-movement control training on female patients with chronic neck pain. | Khosrokiani, Zohreh; Letafatkar, Amir; Sokhanguei, Yahya | | 2018 | Journal of bodywork and movement therapies | Not aerobic exercise |
| Tension neck and evaluation of a physical training course among office workers in a bank corporation. | Klemetti, M; Santavirta, N; Sarvimaki, A; Bjorvell, H | | 1997 | Journal of advanced nursing | Recommendation |
| Tension neck and evaluation of a physical training course among o ce workers in a bank corporation. | Klemetti, Margita; Santavirta, Nina; Sarvimaki, Anneli; Bjorvell, Hjordis | | 1997 | Journal of Advanced Nursing (Wiley-Blackwell) | Not full text available |
| The effect of natural-therapy yoga on the people in chronic neck-shoulder-pain: A Randomized controlled trial | Koo J.W.; Lee K.H. | | 2014 | Pain Practice | Not aerobic exercise |
| Treatment of myogenic temporomandibular disorder: a prospective randomized clinical trial, comparing a mechanical stretching device (TheraBite R) with standard physical therapy exercise. | Kraaijenga, Sophie; van der Molen, Lisette; van Tinteren, Harm; Hilgers, Frans; Smeele, Ludi | | 2014 | Cranio : the journal of craniomandibular practice | Not aerobic exercise |
| Treatment of myogenic temporomandibular disorder: A prospective randomized clinical trial, comparing a mechanical stretching device (TheraBite) with standard physical therapy exercise | Kraaijenga S.; van Der Molen L.; van Tinteren H.; Hilgers F.; Smeele L. | | 2014 | Cranio - Journal of Craniomandibular Practice | Not full text available |
| The effects of aerobic exercise for persons with migraine and co-existing tension-type headache and neck pain. A randomized, controlled, clinical trial. | Kroll, Lotte Skytte; Hammarlund, Catharina Sjodahl; Linde, Mattias; Gard, Gunvor; Jensen, Rigmor Hojland | | 2018 | Cephalalgia : an international journal of headache | Not aerobic exercise |
| Has aerobic exercise effect on pain perception in persons with migraine and coexisting tension-type headache and neck pain? A randomized, controlled, clinical trial. | Kroll, L S; Sjodahl Hammarlund, C; Gard, G; Jensen, R H; Bendtsen, L | | 2018 | European journal of pain (London, England) | Not full text available |
| Factors associated with symptom reduction following different exercise interventions in chronic whiplash associated disorders. A randomized clinical trial | Landen Ludvigsson M.; Peterson G.; Dedering A.; Falla D.; Peolsson A. | | 2015 | Physiotherapy (United Kingdom) | Not aerobic exercise |
| Cost-effectiveness of neck-specific exercise in the treatment of chronic whiplash associated disorders | Landen Ludvigsson M.; Peolsson A.; Peterson G.; Dedering A.; Johansson G.; Bernfort L. | | 2016 | Manual Therapy | Not aerobic exercise |
| Comparison of 2 Manual Therapy and Exercise Protocols for Cervical Radiculopathy: a Randomized Clinical Trial Evaluating Short-Term Effects | LANGEVIN, PIERRE; DESMEULES, FRANÃ‡OIS; LAMOTHE, MÃ‰LANIE; ROBITAILLE, SIMON; ROY, JEAN-SÃ‰BASTIEN | | 2015 | J Orthop Sports Phys Ther | Not full text available |
| Qigong and exercise therapy in patients with long-term neck pain: a prospective randomized trial. | Lansinger, Birgitta; Larsson, Elisabeth; Persson, Liselott C; Carlsson, Jane Y | | 2007 | Spine | Not aerobic exercise |
| Qigong and experience therapy in patients with long-term neck pain: a prospective randomized trial. | Lansinger V; Larsson E; Persson LC; Carlsson JY | | 2007 | Spine (03622436) | Not aerobic exercise |
| Qigong and exercise therapy in patients with long-term neck pain: A prospective randomized trial | Lansinger B. | | 2011 | Physiotherapy (United Kingdom) | Not aerobic exercise |
| The Effects of Tai Chi and Neck Exercises in the Treatment of Chronic Nonspecific Neck Pain: A Randomized Controlled Trial. | Lauche, Romy; Stumpe, Christoph; Fehr, Johannes; Cramer, Holger; Cheng, Ying Wu; Wayne, Peter M; Rampp, Thomas; Langhorst, Jost; Dobos, Gustav | | 2016 | The journal of pain : official journal of the American Pain Society | Not aerobic exercise |
| Spinal manipulation, medication or home exercise for acute and subacute neck pain | Lawrence, D | | 2012 | Ann Intern Med | Conference abstract |
| Clinical effectiveness of a Pilates treatment for forward head posture. | Lee, Sun-Myung; Lee, Chang-Hyung; O'Sullivan, David; Jung, Joo-Ha; Park, Jung-Jun | | 2016 | Journal of physical therapy science | Not aerobic exercise |
| Changes and Associations between Cervical Range of Motion, Pain, Temporomandibular Joint Range of Motion and Quality of Life in Individuals with Migraine Applying Physiotherapy: A Pilot Study. | Lendraitiene, Egle; Smilgiene, Laura; Petruseviciene, Daiva; Savickas, Raimondas | | 2021 | Medicina (Kaunas, Lithuania) | Not musculoskeletal pain |
| The effect of yoga exercise on the rehabilitation of cervical spondylosis in modern young people | Liu L. | | 2020 | Indian Journal of Pharmaceutical Sciences | Not aerobic exercise |
| Effects of a Family Caregiver Care Programme in Musculoskeletal Pain and Disability in the Shoulder-Neck Region-A Randomised Clinical Trial | Llamas-Ramos R.; Barrero-Santiago L.; Llamas-Ramos I.; Montero-Cuadrado F. | | 2023 | International Journal of Environmental Research and Public Health | Not aerobic exercise |
| Neck treatment compared to aerobic exercise in migraine: A preference-based clinical trial | Luedtke K.; Starke W.; Korn K.V.; Szikszay T.M.; Schwarz A.; May A. | | 2020 | Cephalalgia Reports | Not full text available |
| Physical exercise for chronic neck pain | Lundberg G.D. | | 2008 | MedGenMed Medscape General Medicine | Recommendation |
| Effects of an integrated neuromuscular inhibition technique program on neck muscle strength and endurance in individuals with chronic mechanical neck pain. | Lytras, Dimitrios; Sykaras, Evaggelos; Christoulas, Kosmas; Myrogiannis, Ioannis; Kellis, Eleftherios | | 2019 | Journal of bodywork and movement therapies | Not aerobic exercise |
| Comparing biofeedback with active exercise and passive treatment for the management of work-related neck and shoulder pain: a randomized controlled trial. | Ma, Chao; Szeto, Grace P; Yan, Tiebin; Wu, Shaoling; Lin, Caina; Li, Lijuan | | 2011 | Archives of physical medicine and rehabilitation | Not aerobic exercise |
| Clinical effect of Ã¢â‚¬Å“Tai Chi spinal exerciseÃ¢â‚¬ï¿½ on spinal motor function in patients with axial spondyloarthritis | Ma, C; Qu, K; Wen, B; Zhang, Q; Gu, W; Liu, X; Shao, P; Shi, Y; Wang, B | | 2020 | Medicine | Not aerobic exercise |
| Individually tailored exercise and telephone-delivered cognitive behaviour therapy in the management of chronic widespread pain (CWP): results from a primary care based randomised controlled trial | Macfarlane, GJ; Gkazinou, C; Beasley, M; Jones, EA; Prescott, GJ; Hannaford, P; Keeley, P | | 2010 |  | Conference abstract |
| Effect of Pilates Mat Exercise on Myoelectric activity of cervical Muscles in Patient with Chronic Mechanical Neck Pain: Randomized Clinical Trial | Mahmoud, M.S.; El-Kablawy, M.A.; Abd El-Azeim, A.S. | | 2022 | Egyptian Journal of Chemistry | Not aerobic exercise |
| Spinal manipulative therapy, supervised rehabilitative exercise and home exercise for seniors with neck pain | Maiers M.; Bronfort G.; Evans R.; Hartvigsen J.; Svendsen K.; Bracha Y.; Schulz C.; Schulz K.; Grimm R. | | 2012 | BMC Complementary and Alternative Medicine | Not aerobic exercise |
| Effect of Pain Neuroscience Education Combined With Cognition-Targeted Motor Control Training on Chronic Spinal Pain: A Randomized Clinical Trial. | Malfliet, Anneleen; Kregel, Jeroen; Coppieters, Iris; De Pauw, Robby; Meeus, Mira; Roussel, Nathalie; Cagnie, Barbara; Danneels, Lieven; Nijs, Jo | | 2018 | JAMA neurology | Not aerobic exercise |
| Effect of an exercise program on musculoskeletal symptoms in primary school teachers | Manrique-Collantes R.V. | | 2020 | Anales de la Facultad de Medicina | Not aerobic exercise |
| A randomised control trial of preventive spinal manipulation with and without a home exercise program for patients with chronic neck pain | Martel, J; Dugas, C; Dubois, J; Descarreaux, M | | 2011 | BMC Musculoskelet Disord. | Recommendation |
| Pain neuroscience education plus exercise compared with exercise in university students with chronic idiopathic neck pain. | Matias, Beatriz A; Vieira, Isabel; Pereira, Artur; Duarte, MÃ¡rio; Silva, Anabela G | | 2019 | International Journal of Therapy & Rehabilitation | Not aerobic exercise |
| Increasing physical activity in older people with pain. Preliminary results of the ipopp pilot trial | McBeth J.; Nicholls E.; Healey E.; Foster N.E.; Hay E.M.; Pincus T.; Dent S.; Chew-Graham C.A.; Hartshorne L.; Jinks C. | | 2017 | Annals of the Rheumatic Diseases | Not musculoskeletal pain |
| A randomised controlled trial comparing graded exercise treatment and usual physiotherapy for patients with non-specific neck pain (the GET UP neck pain trial). | McLean, Sionnadh M; Klaber Moffett, Jennifer A; Sharp, Donald M; Gardiner, Eric | | 2013 | Manual therapy | Not aerobic exercise |
| Dysfunctional endogenous pain inhibition during exercise in patients with musculoskeletal pain: To exercise or not to exercise? | Meeus M.; Kosek E.; Van Oosterwijck J.; Nijs J. | | 2011 | European Journal of Pain Supplements | Not full text available |
| Comprehensive physiotherapy exercise programme or advice for chronic whiplash (promise): A pragmatic randomised controlled trial | Michaleff Z.; Maher C.; Lin C.-W.C.; Rebbeck T.; Jull G.; Latimer J.; Connelly L.; Sterling M. | | 2015 | Physiotherapy (United Kingdom) | Not aerobic exercise |
| Yoga for chronic neck pain: a pilot randomized controlled clinical trial. | Michalsen, Andreas; Traitteur, Hermann; Ludtke, Rainer; Brunnhuber, Stefan; Meier, Larissa; Jeitler, Michael; Bussing, Arndt; Kessler, Christian | | 2012 | The journal of pain : official journal of the American Pain Society | Not aerobic exercise |
| Spinal manipulative therapy and exercise for seniors with chronic neck pain | Michele, Maiers; Gert, Brontfort; Roni, Evans; Jan, Hartvigsen; Kenneth, Svendsen; Yiscah, Bracha | | 2013 | Spine J | Not aerobic exercise |
| Cervicothoracic Manual Therapy Plus Exercise Therapy Versus Exercise Therapy Alone in the Management of Individuals With Shoulder Pain: A Multicenter Randomized Controlled Trial. | Mintken, Paul E; McDevitt, Amy W; Cleland, Joshua A; Boyles, Robert E; Beardslee, Amber R; Burns, Scott A; Haberl, Matthew D; Hinrichs, Lauren A; Michener, Lori A | | 2016 | The Journal of orthopaedic and sports physical therapy | Not aerobic exercise |
| Effects of therapeutic exercise in TMDs with pain | Moleirinho-Alves P.; Benzinho T.; Paco M. | | 2019 | Annals of Medicine | Not aerobic exercise |
| Effects of two programs with aerobic exercise in headache attributed to temporomandibular disorder | Moleirinho-Alves P.; Almeida A.; Cebola P.; Oliveira R.; Pezarat-Correia P. | | 2021 | Cephalalgia | Conference abstract |
| Effects of two programs with aerobic exercise in headache attributed to temporomandibular disorder | Moleirinho-Alves P.; Almeida A.; Cebola P.; Oliveira R.; Pezarat-Correia P. | | 2021 | Journal of Headache and Pain | Conference abstract |
| Effectiveness of an educational and physical programme in reducing headache, neck and shoulder pain: A workplace controlled trial | Mongini F.; Ciccone G.; Rota E.; Ferrero L.; Ugolini A.; Evangelista A.; Ceccarelli M.; Galassi C. | | 2008 | Cephalalgia | Recommendation |
| An educational and physical program to reduce headache, neck/shoulder pain in a working community: a cluster-randomized controlled trial. | Mongini, Franco; Evangelista, Andrea; Milani, Chantal; Ferrero, Luca; Ciccone, Giovannino; Ugolini, Alessandro; Piedimonte, Alessandro; Sigaudo, Monica; Carlino, Elisa; Banzatti, Emanuela; Galassi, Claudia | | 2012 | PloS one | Not aerobic exercise |
| Chronic neck pain and treatment of cognitive and behavioural factors: results of a randomised controlled clinical trial. | Monticone, Marco; Baiardi, Paola; Vanti, Carla; Ferrari, Silvano; Nava, Tiziana; Montironi, Catia; Rocca, Barbara; Foti, Calogero; Teli, Marco | | 2012 | European spine journal : official publication of the European Spine Society, the European Spinal Deformity Society, and the European Section of the Cervical Spine Research Society | Not aerobic exercise |
| Group-based multimodal exercises integrated with cognitive-behavioural therapy improve disability, pain and quality of life of subjects with chronic neck pain: a randomized controlled trial with one-year follow-up. | Monticone, Marco; Ambrosini, Emilia; Rocca, Barbara; Cazzaniga, Daniele; Liquori, Valentina; Pedrocchi, Alessandra; Vernon, Howard | | 2017 | Clinical rehabilitation | Not full text available |
| A randomized clinical trial assessing the efficacy of adding 6 x 6 exercises to self-care for the treatment of masticatory myofascial pain. | Mulet, Mariona; Decker, Karen L; Look, John O; Lenton, Patricia A; Schiffman, Eric L | | 2007 | Journal of orofacial pain | Not aerobic exercise |
| Isometric training to treat chronic neck pain...Ylinen J, Takala EP, NykÃ¤nen M et al. Active neck muscle training in the treatment of chronic neck pain in women: a randomized controlled trial. JAMA. 2003;289:2509-2516 | Nadler SF; Ylinen J; Nadler, Scott F | | 2003 | JAMA: Journal of the American Medical Association | Not aerobic exercise |
| A study to observe the effects of physiotherapy with and without manual therapy in the management postural neck pain: A randomized control trial. | Nasir, Muhammad Fareed; Jawed, Rubab; Baig, Nabeel Naeem; Younus, Maryam; Arshad, Ayesha; Tahir, Aisha | | 2021 | JPMA. The Journal of the Pakistan Medical Association | Not aerobic exercise |
| The Effect of Nonstrenuous Aerobic Exercise in Patients with Chronic Masticatory Myalgia. | Nasri-Heir, Cibele; Patil, Amey G; Korczeniewska, Olga A; Zusman, Tal; Khan, Junad; Heir, Gary; Benoliel, Rafael; Eliav, Eli | | 2019 | Journal of oral & facial pain and headache | Not RCT |
| Manual Therapy Versus Exercise on Knee Osteoarthritis | NCT00988468, | | 2009 |  | Not aerobic exercise |
| Work Place Adjusted Intelligent Physical Exercise Reducing Musculoskeletal Pain in Shoulder and Neck (VIMS) - Industrial Workers | NCT01071980, | | 2010 |  | Not aerobic exercise |
| Effect of Kettlebell Training on Musculoskeletal and Cardiovascular Health | NCT01076127, | | 2010 |  | Not aerobic exercise |
| Exercise Training With Physically Active Lifestyle to Reduce Headache and Quality of Life | NCT01664585, | | 2012 |  | Not aerobic exercise |
| The Effects of Two Different Home-exercise Programmes on Women Suffering Long Term Neck/Shoulder Muscle Pain | NCT01876680, | | 2011 |  | Not aerobic exercise |
| Implementation of Physical Exercise at the Workplace (IRMA08) - Healthcare Workers | NCT01921764, | | 2013 |  | Clinical trial registration / Trial no finished |
| Effect of Group Pilates and Yoga Exercise Classes for Chronic Cervical Pain | NCT01999283, | | 2013 |  | Not aerobic exercise |
| Effect of the Method Pilates in Women With Temporomandibular Disorders | NCT02292355, | | 2014 |  | Not full text available |
| Pilates to Treat Neck Pain | NCT02433821, | | 2013 |  | Not aerobic exercise |
| Clinical Prediction Rules for Identifying Patients With Chronic Neck Pain Who Will Benefit From General Aerobic Exercise | NCT02451267, | | 2015 |  | Not full text available |
| Effectiveness of the Pilates Method Versus Aerobic Exercises in Elderly With Low Back Pain | NCT02729779, | | 2016 |  | Clinical trial registration / Trial no finished |
| Telemedicine to Manage Chronic Neck Pain at Home | NCT02736851, | | 2016 |  | Not aerobic exercise |
| Long-Term Effectiveness of Walking Training in Patients With Knee Osteoarthritis | NCT02767570, | | 2016 |  | Clinical trial registration / Trial no finished |
| Neck-specific Exercise in Chronic Whiplash | NCT03022812, | | 2017 |  | Not aerobic exercise |
| Efficacy of Deep Cervical Flexor Muscles Training on Neck Pain, Functional Disability and Muscle Endurance in School Teachers | NCT03537300, | | 2018 |  | Not aerobic exercise |
| Predictive Model of Recovery in Patients With Chronic Nonspecific Neck Pain Undergoing Manual Therapy and Exercise | NCT03562338, | | 2018 |  | Not aerobic exercise |
| Efficiency of Modified Pilates Exercises in Patients With Chronic Neck Pain | NCT03782584, | | 2018 |  | Not aerobic exercise |
| The Effectiveness Yoga@Work Among Office Workers With Chronic Nonspecific Neck Pain (CNNP) | NCT04113460, | | 2019 |  | Not aerobic exercise |
| An Evaluation of Yoga Therapy for Cervical Spondylosis | NCT04113473, | | 2019 |  | Not aerobic exercise |
| Efficacyof Pilates Exercises in Lowback Pain | NCT04135131, | | 2019 |  | Clinical trial registration / Trial no finished |
| Efficacy Of Different Types Of Physiotherapy Approaches In Temporomandibular Disorders | NCT04294602, | | 2020 |  | Clinical trial registration / Trial no finished |
| EFFECTS OF ADDING TWO DIFFERENT TYPES OF MANUAL TECHNIQUES TO A THERAPEUTIC EXERCISE PROGRAM FOR THE MANAGEMENT OF CHRONIC NECK PAIN: a RANDOMIZED CONTROLLED TRIAL OF COMPARATIVE EFFECTIVENESS | NCT04327739, | | 2020 |  | Not aerobic exercise |
| Effects of Manual Therapy on the Upper Cervical Spine Combined With Exercise vs Isolated Exercise in Patients With Cervicogenic Headache | NCT04401501, | | 2020 |  | Not aerobic exercise |
| Effects of Postural Global Reeducation Versus Therapeutic Exercise in Chronic Non-specific Neck Pain | NCT04402463, | | 2020 |  | Not aerobic exercise |
| Effectiveness of Mulligan Mobilization Technique And Cervical Stabilization Training in Patients With Chronic Neck Pain | NCT04643028, | | 2020 |  | Not aerobic exercise |
| Effects of Manual Therapy and Exercise Training of Diaphragm in Patients With Chronic Neck Pain | NCT04664842, | | 2020 |  | Not aerobic exercise |
| The NEXERCISE-trial: reshaping Exercise Programs for Patients With Non-specific Neck Pain | NCT04749823, | | 2021 |  | Clinical trial registration / Trial no finished |
| EFFECTS OF THERAPATIC EXERCISES AND STABILIZATION EXERCISES AFTER MANUAL THERAPY IN PATIENTS WITH NON-SPECIFIC CHRONIC NECK PAIN | NCT04809337, | | 2021 |  | Not aerobic exercise |
| Effectiveness of Therapeutic Exercise Versus Manual Therapy in Patients With Chronic Neck Pain | NCT05255055, | | 2022 |  | Clinical trial registration / Trial no finished |
| Effect of a Home-based Versus Supervised Exercise Program in Patients With Migraine: a Randomized Clinical Trial | NCT05292599, | | 2022 |  | Clinical trial registration / Trial no finished |
| Effectiveness of Two Exercise Programs on the Neck. A Randomized Controlled Trial | NCT05331482, | | 2022 |  | Clinical trial registration / Trial no finished |
| The Effects of Arm Ergometer Endurance Training and High-intensity Interval Training in Chronic Neck Pain | NCT05485220, | | 2022 |  | Clinical trial registration / Trial no finished |
| Effects of Aerobic Exercise on Pain in Patients With Myogenic Temporomandibular Disorders | NCT05540366, | | 2022 |  | Clinical trial registration / Trial no finished |
| Efficacy of pressure-biofeedback guided deep cervical flexor training on neck pain and muscle performance in visual display terminal operators | Nezamuddin M.; Anwer S.; Khan S.A.; Equebal A. | | 2013 | Journal of Musculoskeletal Research | Not aerobic exercise |
| Effectiveness of exercise therapy in patients with myofascial pain dysfunction syndrome. | Nicolakis, P; Erdogmus, B; Kopf, A; Nicolakis, M; Piehslinger, E; Fialka-Moser, V | | 2002 | Journal of oral rehabilitation | Not aerobic exercise |
| Dose-response relationship of specific training to reduce chronic neck pain and disability. | Nikander, Riku; Malkia, Esko; Parkkari, Jari; Heinonen, Ari; Starck, Heli; Ylinen, Jari | | 2006 | Medicine and science in sports and exercise | Not aerobic exercise |
| Comparison of two physical exercise programs for the early intervention of pain in the neck, shoulders and lower back in female hospital staff. | Oldervoll, L M; Ro, M; Zwart, J A; Svebak, S | | 2001 | Journal of rehabilitation medicine | Not aerobic exercise |
| Training mode-dependent changes in motor performance in neck pain. | O'Leary, Shaun; Jull, Gwendolen; Kim, Mehwa; Uthaikhup, Sureeporn; Vicenzino, Bill | | 2012 | Archives of physical medicine and rehabilitation | Not aerobic exercise |
| Effects of aerobic exercise on pain sensitivity, heart rate recovery, and health-related quality of life in patients with chronic musculoskeletal pain. | Ote Karaca, Seyda; Demirsoy, Nesrin; Gunendi, Zafer | | 2017 | International journal of rehabilitation research. Internationale Zeitschrift fur Rehabilitationsforschung. Revue internationale de recherches de readaptation | Not aerobic exercise |
| The effect of neck-specific exercise with or without a behavioral approach on psychological factors in chronic whiplash-associated disorders: A randomized controlled trial with a 2-year follow-up. | Overmeer, Thomas; Peterson, Gunnel; Landen Ludvigsson, Maria; Peolsson, Anneli | | 2016 | Medicine | Not aerobic exercise |
| The effectiveness of telerehabilitation-based structured exercise therapy for chronic nonspecific neck pain: A randomized controlled trial. | Ozel, Merve; Kaya Ciddi, Pinar | | 2022 | Journal of telemedicine and telecare | Not aerobic exercise |
| Comparative Efficacy of Neck Stabilization and Pilates Exercises on Pain, Sleep disorder and Kinesiophobia in Patients with Non-Specific Chronic Neck Pain | PACTR201807573146508, | | 2018 |  | Not aerobic exercise |
| Effects of Pilates and Isometric Neck Strengthening Exercises on Pain, Neck Disability, Psychological Status and Sleep Disturbance in Patients with Non-specific Chronic Neck Pain | PACTR202106715244474, | | 2020 |  | Clinical trial registration / Trial no finished |
| The effect of muscle strength and endurance training on muscoloskeletal complaints in the shoulder/neck region of cashier checkout workers | Pedersen, M.T.; Mikkelsen, S.; Fallentin, N. | | 2000 | Proceedings of the XIVth Triennial Congress of the International Ergonomics Association and 44th Annual Meeting of the Human Factors and Ergonomics Association, 'Ergonomics for the New Millennium' | Not aerobic exercise |
| Effect of specific resistance training on musculoskeletal pain symptoms: dose-response relationship. | Pedersen, Mogens T; Andersen, Lars L; Jorgensen, Marie B; Sogaard, Karen; Sjogaard, Gisela | | 2013 | Journal of strength and conditioning research | Not aerobic exercise |
| Investigation of effects different physiotherapy methods on pain and quality of life in patients with temporomandibular joint dysfunction | Pehlivan Tekin G.; Yakut Y.; Agirnas Kartal E. | | 2018 | Fizyoterapi Rehabilitasyon | Not aerobic exercise |
| Back pain and Adapted Physical Activity: Results from the experience in ASL 4 chiavarese of Liguria | Piastra G.; Bravo M.F.; Lucarini S.; Cavagnaro P. | | 2012 | Giornale di Gerontologia | Not aerobic exercise |
| Comparison of the effectiveness of a behavioural graded activity program and manual therapy in patients with sub-acute neck pain: Design of a randomized clinical trial | Pool J.J.M.; Ostelo R.W.J.G.; Koke A.J.; Bouter L.M.; de Vet H.C.W. | | 2006 | Manual Therapy | Not aerobic exercise |
| Is a behavioral graded activity program more effective than manual therapy in patients with subacute neck pain? Results of a randomized clinical trial. | Pool, Jan J M; Ostelo, Raymond W J G; Knol, Dirk L; Vlaeyen, Johan W S; Bouter, Lex M; de Vet, Henrica C W | | 2010 | Spine | Not aerobic exercise |
| A randomized trial comparing manual physical therapy to therapeutic exercises, to a combination of therapies, for the treatment of cervical radiculopathy. | Ragonese J | | 2009 | Orthopaedic Physical Therapy Practice | Not aerobic exercise |
| The Effects of Thoracic Thrust Manipulation and Neck Flexibility Exercises for the Management of the Patients with Mechanical Neck Pain. | Raja, R.; Kotteeswaran, K.; Anandh, V. | | 2015 | Indian Journal of Physiotherapy & Occupational Therapy | Not aerobic exercise |
| To analyse the effectiveness of yoga, pilates and tai chi exercise for chronic mechanical neck pain -a randomized controlled trial | Rajalaxmi V.; Jasim A.; Sudhakar S.; Mohan Kumar G. | | 2018 | Biomedicine (India) | Not aerobic exercise |
| Efficacy of Endurance vs Isometric neck exercise in chronic non - specific neck pain: A RCT | Rajalaxmi, V.; Paul, J.; Manoj Abraham, M.; Sasirekha, M. | | 2019 | Indian Journal of Forensic Medicine and Pathology | Not aerobic exercise |
| Intensive dynamic training for females with chronic neck/shoulder pain. A randomized controlled trial. | Randlov, A; Ostergaard, M; Manniche, C; Kryger, P; Jordan, A; Heegaard, S; Holm, B | | 1998 | Clinical rehabilitation | Not aerobic exercise |
| A tailored workplace exercise program for women at risk for neck and upper limb musculoskeletal disorders: a randomized controlled trial. | Rasotto, Chiara; Bergamin, Marco; Sieverdes, John C; Gobbo, Stefano; Alberton, Cristine L; Neunhaeuserer, Daniel; Maso, Stefano; Zaccaria, Marco; Ermolao, Andrea | | 2015 | Journal of occupational and environmental medicine | Not aerobic exercise |
| A Tailored Workplace Exercise Program for Women at Risk for Neck and Upper Limb Musculoskeletal Disorders. | Rasotto, Chiara; Bergamin, Marco; Sieverdes, John C.; Gobbo, Stefano; Alberton, Cristine L.; Neunhaeuserer, Daniel; Maso, Stefano; Zaccaria, Marco; Ermolao, Andrea | | 2015 | Journal of Occupational & Environmental Medicine | Not aerobic exercise |
| Effectiveness of distance exercises associated with self management booklet in patients with neck pain | RBR-10h7khvk, | | 2022 |  | Clinical trial registration / Trial no finished |
| Influence of Physical Exercise on pain modulation and on psychological, social and behavioral aspects in patients with Chronic Pain of masticatory muscles | RBR-25jpgt, | | 2020 |  | Clinical trial registration / Trial no finished |
| Exercise and pain education in individuals with chronic pain | RBR-38krf2, | | 2019 |  | Clinical trial registration / Trial no finished |
| Impact of a Program of Subaquatic Therapeutic Exercises in Elderly Women with Knee Osteoarthritis | RBR-8f57kr, | | 2013 |  | Clinical trial registration / Trial no finished |
| Effects of two different exercise protocols for treating women with jaw pain | RBR-98b36rc, | | 2022 |  | Clinical trial registration / Trial no finished |
| Qigong versus exercise versus no therapy for patients with chronic neck pain - a randomized controlled trial | Rendant, D; Pach, D; Ludtke, R; Reisshauer, A; Willich, S; Witt, CM | | 2010 |  | Not aerobic exercise |
| Qigong versus exercise versus no therapy for patients with chronic neck pain: a randomized controlled trial. | Rendant, Daniel; Pach, Daniel; Ludtke, Rainer; Reisshauer, Anett; Mietzner, Anna; Willich, Stefan N; Witt, Claudia M | | 2011 | Spine | Not aerobic exercise |
| Therapeutic Exercise Training to Reduce Chronic Headache in Working Women: Design of a Randomized Controlled Trial. | Rinne, Marjo; Garam, Sanna; Hakkinen, Arja; Ylinen, Jari; Kukkonen-Harjula, Katriina; Nikander, Riku | | 2016 | Physical therapy | Not aerobic exercise |
| Does a combination of physical training, specific exercises and pain education improve health-related quality of life in patients with chronic neck pain? A randomised control trial with a 4-month follow up | Ris, I; Sogaard, K; Gram, B; Agerbo, K; Boyle, E; Juul-Kristensen, B | | 2016 |  | Not aerobic exercise |
| Specific neck training induces sustained corticomotor hyperexcitability as assessed by motor evoked potentials. | Rittig-Rasmussen, Bjarne; Kasch, Helge; Fuglsang-Frederiksen, Anders; Jensen, Troels S; Svensson, Peter | | 2013 | Spine | Not aerobic exercise |
| On "Manual therapy, exercise, and traction for patients with cervical radiculopathy: A randomized clinical trial" | Robertson E.K. | | 2009 | Physical Therapy | Not aerobic exercise |
| Effect of strength training in addition to general exercise in the rehabilitation of patients with non-specific neck pain. A randomized clinical trial. | Rolving, N; Christiansen, D H; Andersen, L L; Skotte, J; Ylinen, J; Jensen, O K; Nielsen, C V; Jensen, C | | 2014 |  | Not aerobic exercise |
| Reducing pain and disability for patients with chronic neck pain : results of a double-blind randomised controlled trial comparing strength to endurance training | Ryan, JM | | 2002 |  | Not aerobic exercise |
| Traditional physical therapy exercises combined with sensorimotor training: The effects on clinical outcomes for chronic neck pain in a double-blind, randomized controlled trial. | Saadat, Maryam; Salehi, Reza; Negahban, Hossein; Shaterzadeh, Mohammad Jafar; Mehravar, Mohammad; Hessam, Masumeh | | 2019 | Journal of bodywork and movement therapies | Not aerobic exercise |
| Comparison of effects of manual physical therapy and exercise therapy for patients with Temporomandibular disorders | Sarfraz, S.; Anwar, N.; Tauqeer, S.; Asif, T.; Ul Ain, N.; Shakeel, H. | | 2023 | Journal of the Pakistan Medical Association | Not RCT |
| Yoga improves occupational performance, depression, and daily activities for people with chronic pain. | Schmid, Arlene A; Van Puymbroeck, Marieke; Fruhauf, Christine A; Bair, Matthew J; Portz, Jennifer Dickman | | 2019 | Work (Reading, Mass.) | Not aerobic exercise |
| Becoming active again? Further thoughts on goal pursuit in chronic pain | Schrooten M.G.S.; Vlaeyen J.W.S. | | 2010 | Pain | Not RCT |
| Examining the Impact and Feasibility of a Self-Efficacy Based Walking Intervention for Persons with Chronic Non-Specific Neck Pain. | Scibilia, Marisa B. | | 2017 | University of Delaware | Not aerobic exercise |
| Long-term follow-up of tailored behavioural treatment and exercise based physical therapy in persistent musculoskeletal pain: A randomized controlled trial in primary care. | Senlof, Pernilla; Denison, Eva; Lindberg, Per | | 2009 | European journal of pain (London, England) | Not aerobic exercise |
| Effect of yoga therapy on patients with chronic musculoskeletal pain: a prospective randomised wait list-controlled trial | Sharma N.; John P.J.; Meghwal N.; Owen A.; Mishra V. | | 2019 | Clinical medicine (London, England) | Conference abstract |
| Can a specific exercise program combined with brief counseling by a physical therapist offer benefits over usual care? | Shaughnessy A | | 1999 | Evidence-Based Practice | Not RCT |
| An Analysis of Memory Retrieval and Performance of Physiotherapy Exercises in Younger and Older Patients. | Shete, Dhiraj R. | | 2012 | Indian Journal of Physiotherapy & Occupational Therapy-An International Journal | Not aerobic exercise |
| Exercise training and work task induced metabolic and stress-related mrna and protein responses in myalgic muscles | Sjogaard G.; Zebis M.K.; Kiilerich K.; Saltin B.; Pilegaard H. | | 2013 | BioMed Research International | Not aerobic exercise |
| Effects of a workplace physical exercise intervention on the intensity of headache and neck and shoulder symptoms and upper extremity muscular strength of office workers: a cluster randomized controlled cross-over trial. | Sjogren, Tuulikki; Nissinen, Kari J; Jarvenpaa, Salme K; Ojanen, Markku T; Vanharanta, Heikki; Malkia, Esko A | | 2005 | Pain | Not aerobic exercise |
| The effects of exercise reminder software program on office workers' perceived pain level, work performance and quality of life | Soares, MM; Jacobs, K; Irmak, A; Bum$\dot{{\rm i}}$n, G; Irmak, R | | 2012 | Work | Not aerobic exercise |
| Active exercise program in patient with nonspecific chronic neck pain: a randomised controlled trial | Song, CH; Jang HJ Lee, KJ; Lee, YW | | 2012 |  | Not aerobic exercise |
| The Effect of Postural Correction and Exercise on Neck Pains in Cell Phone Users | Soyer O.; Akarirmak Z.U. | | 2020 | Turk Osteoporoz Dergisi | Not aerobic exercise |
| Altered relationship between anandamide and glutamate in circulation after 30 min of arm cycling: A comparison of chronic pain subject with healthy controls | Stensson N.; Grimby-Ekman A. | | 2019 | Molecular Pain | Not aerobic exercise |
| Randomized controlled trial of exercise for chronic whiplash-associated disorders. | Stewart, Mark J; Maher, Chris G; Refshauge, Kathryn M; Herbert, Rob D; Bogduk, Nikolai; Nicholas, Michael | | 2007 | Pain | Not aerobic exercise |
| Patient and clinician treatment preferences do not moderate the effect of exercise treatment in chronic whiplash-associated disorders. | Stewart, Mark J; Maher, Chris G; Refshauge, Kathryn M; Herbert, Rob D; Nicholas, Michael K | | 2008 | European journal of pain (London, England) | recommendation |
| Effects of intermittent low intensity exercise on the morphological characteristics of deep neck muscles in female patients with chronic neck pain | Sun W. | | 2018 | Indian Journal of Pharmaceutical Sciences | Not aerobic exercise |
| Promoting physical activity in different patient groups. | Sundelin G | | 2009 | Advances in Physiotherapy | Not RCT |
| The Effect of Self-management Exercises on Neck Pain and Head and Neck Angles among Iranian University Employees: An Interventional Study | Tabanfar, S.; Variani, A.S.; Sobhani, S.; Varmazyar, S. | | 2022 | Journal of Occupational Health and Epidemiology | Not aerobic exercise |
| The effects of an eight-week selected therapeutic exercises course and self-treatment by pamphlet programs on the rate of chronic neck pain and disability among computer users | Taheri H.; Mahdavinejad R.; Minasian V.; Karimi A. | | 2012 | Journal of Isfahan Medical School | Not aerobic exercise |
| Immunological modulation by yoga therapy in migraine patients | Talakad S.; Kisan R.; Sujan M.U.; Rao R.; Nalini A.; Trichur R. | | 2013 | Cephalalgia | Not aerobic exercise |
| Efficacy of different combinations of physiotherapy techniques compared to exercise and patient education in temporomandibular disorders: A randomized controlled study. | Tanhan, Abdurrahman; Ozer, Aysel Yildiz; Polat, Mine Gulden | | 2021 | Cranio : the journal of craniomandibular practice | Not aerobic exercise |
| The effect of walking intervention for preventing neck pain among office workers | TCTR20160928001, | | 2016 |  | Not aerobic exercise |
| Evidence that manual therapy is superior to exercise therapy from a small, but carefully conducted trial. | ter Riet G | | 2003 | Focus on Alternative & Complementary Therapies | Conference abstract |
| Effectiveness of yoga in the management of jaw muscle pain | Thimma Ravindranath, P; Peck, C; Murray, G; Klineberg, I; Bhutada, MK | | 2012 | Not reported | Not aerobic exercise |
| Balance, dizziness and proprioception in chronic whiplash with dizziness: An RCT comparing three exercise programs | Treleaven J.; Peterson G.; Landen Ludvigsson M.; Kammerlind A.-S.; Peolsson A. | | 2015 | Physiotherapy (United Kingdom) | Not aerobic exercise |
| Comparing the effectiveness of integrating ergonomics and motor control to conventional treatment for pain and functional recovery of work-related neck-shoulder pain: A randomized trial. | Tsang, Sharon M H; So, Billy C L; Lau, Rufina W L; Dai, Jie; Szeto, Grace P Y | | 2019 | European journal of pain (London, England) | Not aerobic exercise |
| Physical exercise and health education for neck and shoulder complaints among sedentary workers | Tsauo, JY; Lee, HY; Hsu, JH; Chen, CY; Chen, CJ | | 2004 | J Rehabil Med | Not aerobic exercise |
| Evaluation of the efficacy of spa therapy on pain and quality of life in patients with chronic mechanical neck pain | Turel A.; Solak O.; Dundar U.; Toktas H.; Demirdal U.S.; Subasi V.; Kavuncu V. | | 2015 | Archives of Rheumatology | Not aerobic exercise |
| Effects of yoga on balance and gait properties in women with musculoskeletal problems: a pilot study. | Ulger, Ozlem; Yagli, Naciye Vardar | | 2011 | Complementary therapies in clinical practice | Not aerobic exercise |
| Effects of Pilates and yoga in patients with chronic neck pain: A sonographic study. | Ulug, Naime; Yilmaz, Oznur Tunca; Kara, Murat; Ozcakar, Levent | | 2018 | Journal of rehabilitation medicine | Not aerobic exercise |
| Effects of chronic exercise on pain sensitivity and subjective pain symptom in people with chronic pain | UMIN000038850, | | 2019 |  | Clinical trial registration / Trial no finished |
| Effects of software programs stimulating regular breaks and exercises on work-related neck and upper-limb disorders. | van den Heuvel, Swenne G; de Looze, Michiel P; Hildebrandt, Vincent H; The, Kiem H | | 2003 | Scandinavian journal of work, environment & health | Not aerobic exercise |
| Cost-effectiveness of manual therapy versus physical therapy in patients with sub-acute and chronic neck pain: A randomized controlled trial | Van Dongen J.M.; Groeneweg R.; Rubinstein S.M.; Bosmans J.E.; Oostendorp R.A.; Ostelo R.W.; Van Tulder M.W. | | 2015 | Value in Health | Not aerobic exercise |
| Short term treatment versus long term management of neck and back disability in older adults utilizing spinal manipulative therapy and supervised exercise: a parallel-group randomized clinical trial evaluating relative effectiveness and harms. | Vihstadt, Corrie; Maiers, Michele; Westrom, Kristine; Bronfort, Gert; Evans, Roni; Hartvigsen, Jan; Schulz, Craig | | 2014 | Chiropractic & manual therapies | Not aerobic exercise |
| Effectiveness of behavioural graded activity compared with physiotherapy treatment in chronic neck pain: Design of a randomised clinical trial [ISRCTN88733332] | Vonk F.; Verhagen A.P.; Geilen M.; Vos C.J.; Koes B.W. | | 2004 | BMC Musculoskeletal Disorders | Not aerobic exercise |
| Effectiveness of a behaviour graded activity program versus conventional exercise for chronic neck pain patients. | Vonk, Frieke; Verhagen, Arianne P; Twisk, Jos W; Koke, Albere J A; Luiten, Marlies W C T; Koes, Bart W | | 2009 | European journal of pain (London, England) | Not aerobic exercise |
| Qigong and exercise therapy for elderly patients with chronic neck pain (QIBANE): a randomized controlled study. | von Trott, Philipp; Wiedemann, Anna Maria; Ludtke, Rainer; Reishauer, Anett; Willich, Stefan N; Witt, Claudia M | | 2009 | The journal of pain : official journal of the American Pain Society | Not aerobic exercise |
| Perceived pain before and after three exercise programs--a controlled clinical trial of women with work-related trapezius myalgia. | Waling, K; Sundelin, G; Ahlgren, C; Jarvholm, B | | 2000 | Pain | Not aerobic exercise |
| Correlation between aerobic exercise and improvement of physical sub-health based on multiple linear regression | Wang, G. | | 2020 | Proceedings - 2020 12th International Conference on Measuring Technology and Mechatronics Automation, ICMTMA 2020 | Conference abstract |
| Treatment outcome of supervised exercise, home exercise and bite splint therapy, respectively, in patients with symptomatic disc displacement with reduction: A randomised clinical trial. | Wanman, Anders; Marklund, Susanna | | 2020 | Journal of oral rehabilitation | Not aerobic exercise |
| The efficacy of ultrasound-facilitated electrical stimulation as an adjunct to exercise in treating chronic neck and shoulder pain | Waschl S.; Morrissey M.C.; Rugelj D. | | 2014 | Journal of Musculoskeletal Pain | Not aerobic exercise |
| Clinical observation on Yi Jin Jing (Sinew-transforming Qigong Exercises) plus tuina on the neck for stiff neck | Wu, Y.-J.; Zhu, G.-F.; Xu, J. | | 2020 | Journal of Acupuncture and Tuina Science | Not aerobic exercise |
| Effect of Backward Walking in Subjects with Mechanical Neck Pain. | Yagnik, Anjali; Chintamani, Radhika | | 2020 | Indian Journal of Physiotherapy & Occupational Therapy | Not aerobic exercise |
| Effects on Turkish ice cream employees' musculoskeletal pain of a physical activity and ergonomics improvement program in the workplace. | Yalcin, Ilknur; Ergun, Ayse | | 2022 | International journal of occupational safety and ergonomics : JOSE | Not aerobic exercise |
| Comparison of two different delivery methods of home-based exercise on neck pain. | Yasarer, Ozden; Yilmaz, Hurriyet Gursel; Dogan, Halis | | 2023 | Somatosensory & motor research | Not aerobic exercise |
| Both endurance training and strength training reduced disability and pain in chronic nonspecific neck pain in women. | Yelland M | | 2003 | ACP Journal Club | Not aerobic exercise |
| Both endurance training and strenght training reduced disability and pain in chronic non-specific neck pain in women | Ylinen J.; Takala E.P.; Nykanen M. | | 2003 | Evidence-Based Medicine | Not aerobic exercise |
| Active neck muscle training for treatment of women with chronic neck pain: A randomized controlled study | Ylinen, J.; Takala, E.P.; NykÃ¤nen, M.; HÃ¤kkinen, A.; MÃ¤lkiÃ¤, E.; Pohjolainen, T.; Karppi, S.L.; Kautiainen, H.; Airaksinen, O. | | 2003 | Manuelle Medizin | Not aerobic exercise |
| [Exercise of neck and shoulder muscles as a relief for the chronic neck pain]. | Ylinen, Jari; Takala, Esa-Pekka; Nykanen, Matti; Hakkinen, Arja; Kautiainen, Hannu; Malkia, Esko; Pohjolainen, Timo; Karppi, Sirkka-Liisa; Airaksinen, Olavi | | 2004 | Duodecim; laaketieteellinen aikakauskirja | Not aerobic exercise |
| Effects of twelve-month strength training subsequent to twelve-month stretching exercise in treatment of chronic neck pain. | Ylinen, Jari J; Takala, Esa-Pekka; Nykanen, Matti J; Kautiainen, Hannu J; Hakkinen, Arja H; Airaksinen, Olavi V P | | 2006 | Journal of strength and conditioning research | Not aerobic exercise |
| Clinical trials. Reducing fear and promoting activity had beneficial effects in a large randomized trial. |  | | 2005 | Bone & Joint | Not full text available |
| Endurance training to be no more effective than physiotherapy or chiropractic treatment in terms of reducing pain and disability or increasing muscle strength. | Ryan, J; Corry, J; Speldewinde, G | 1998 | | Spine (03622436) | Not RCT |

**Appendix 4 – Summary of Compiled Set of Items (CSoI)**

**Table A4** **Summary of the compiled set of items used to evaluate the risk of bias of the studies included in this systematic review.**

The judgments are shown for each risk of bias item for each included study. The disagreements between the two reviewers were resolved by consensus.

| Items | Andersen et al. 2008(a)(b)  Anderson et al 2009 (d)  Sogaar et al 2012 (c)  Mackey et al. 2011 | Daher et al. 2020  2021  2022 | Eftekharsadat et al. 2018 | Kocur et al.  2017 | Korshoj et al.  2018 | Saeterbakken et al.  2017 | Total (%) |
| --- | --- | --- | --- | --- | --- | --- | --- |
| Inclusion and exclusion criteria clearly defined consensus | Yes | Yes | Yes | Yes | Yes | Yes | 6 (100%) |
| Study described as randomized consensus | Yes | Yes | Yes | Yes | Yes | Yes | 6 (100%) |
| Method of randomization described and appropriate consensus | Unclear | Yes | Yes | Unclear | Yes | No | 3 (50%) |
| Method of randomization concealed consensus | Unclear | Yes | No | Unclear | Yes | Unclear | 2 (33.3%) |
| Baseline comparability regarding the most important prognostic indicators consensus | Unclear | Yes | Yes | Unclear | Yes | No | 3 (50%) |
| Study described as double-blind consensus | No | Yes | No | No | No | No | 1 (16.7%) |
| Method of blinding appropriate consensus | Unclear | Yes | No | Yes | No | Unclear | 2 (33.3%) |
| Blinding of investigator consensus | Unclear | Unclear | No | Unclear | Unclear | Unclear | 0 (0%) |
| Blinding of assessor consensus | No | Yes | No | Yes | No | Unclear | 2 (33.3%) |
| Blinding of subjects/patients consensus | No | Yes | No | No | No | Unclear | 1 (16.7%) |
| Blinding of therapists’ provider consensus | No | No | No | No | No | No | 0 (0%) |
| Blinding of the outcome analysis (statistician) consensus | Unclear | Yes | No | Unclear | Unclear | Unclear | 1 (16.7%) |
| Treatment protocol adequately described for the treatment group consensus | Yes | Yes | Yes | Yes | Yes | Yes | 6 (100%) |
| Treatment protocol adequately described for the control or comparison group consensus | Yes | Yes | Yes | Unclear | Yes | Yes | 5 (83.3%) |
| Control group adequate consensus | Yes | No | No | Unclear | No | Unclear | 1 (16.7%) |
| Placebo group adequate consensus | No | No | No | No | No | No | 0 (0%) |
| Co-interventions avoided or comparable consensus | Unclear | Yes | Yes | Unclear | Unclear | Unclear | 2 (33.3%) |
| Co-interventions reported for each group separately consensus | No | Yes | No | No | Unclear | No | 1 (16.7%) |
| Testing of subject compliance to treatment protocol consensus | Yes | Yes | Unclear | Unclear | Yes | Yes | 4 (66.7%) |
| Compliance acceptable in all group consensus | Yes | Unclear | Unclear | Unclear | No | No | 1 (16.7%) |
| Description of withdraws and dropout consensus | Yes | Yes | Yes | Yes | Unclear | No | 4 (66.7%) |
| Withdrawal/dropouts rate describe and acceptable | Yes | Yes | Yes | Yes | No | Yes | 5 (83.3%) |
| Reasons for dropout consensus | No | Yes | Unclear | Yes | Yes | No | 3 (50%) |
| Adverse effects described consensus | No | Yes | No | No | Unclear | No | 1 (16.7%) |
| Short term follow measurement performed consensus | Yes | No | Yes | No | No | Yes | 3 (50%) |
| Long term follow measurement performed consensus | No | Yes | No | No | Yes | No | 2 (33.3%) |
| The timing of the outcome assessment was comparable in all groups consensus | Yes | Yes | Yes | Yes | Yes | Yes | 6 (100%) |
| Description of outcome measures consensus | Yes | Yes | Unclear | Yes | Yes | Yes | 5 (83.3%) |
| Relevant outcomes were used consensus | Yes | Yes | Yes | Yes | Yes | Yes | 6 (100%) |
| Validity reported for main outcome measure consensus | No | Yes | Yes | No | No | No | 2 (33.3%) |
| Responsiveness for main outcome measure consensus | No | No | No | No | No | No | 0 (0%) |
| Reliability reported for main outcome measure consensus | No | Yes | Yes | No | No | No | 2 (33.3%) |
| Use of objective outcome measures consensus | Yes | Yes | Yes | Yes | Yes | Yes | 6 (100%) |
| Descriptive measures identified and reported for the primary outcome consensus | Yes | Yes | Yes | Yes | Yes | Yes | 6 (100%) |
| Appropriate statistical analysis used consensus | Yes | Yes | Yes | Yes | Yes | Yes | 6 (100%) |
| Sample size calculation performed prior to initiation of the study consensus | Yes | Yes | No | Unclear | Yes | No | 3 (50%) |
| Adequate sample size consensus | No | Yes | Unclear | Unclear | Yes | Unclear | 2 (33.3%) |
| Sample size described for each group consensus | Yes | Yes | Yes | Yes | Yes | Yes | 6 (100%) |
| Intention to treat analysis used consensus | No | Yes | No | No | Yes | No | 2 (33.3%) |
| Clinical Significance reported consensus | No | Yes | No | No | No | Yes | 2 (33.3%) |
| Number of items accomplished/ total of applicable items | 18/40 | 33/40 | 18/40 | 15/40 | 20/40 | 15/40 |  |
| Number of items applicable | 40 | 40 | 40 | 40 | 40 | 40 |  |
| % of items accomplished | 45% | 82.5% | 45% | 37.5% | 50% | 37.5% |  |
| Risk of bias Tool Assessment | High | High | High | High | High | High |  |

**Appendix 5 – GRADE approach results**

***Table A5.*** *GRADE Evidence profile for aerobic exercise vs. control groups and other interventions.*

| **Domains assessment** | | | | | | | **Summary of findings** | | | | |
| --- | --- | --- | --- | --- | --- | --- | --- | --- | --- | --- | --- |
|  |  |  |  |  |  |  | **№ of patients Aerobic** | **№ of patients Control** | **Estimate MD  [95%CI]** | **Quality** | **Importance** |
| **№ of studies** | **Study design** | **Risk of bias** | **Inconsistency** | **Indirectness** | **Imprecision** | **Publication bias** |  |  |  |  |  |
| **A5.1. Aerobic exercise vs. non-treatment**  **Pain intensity in general: mean after the end of the treatment; assessed with VAS (0-100mm)** | | | | | | | | | | | |
| 1) Andersen (a) et al., (2008)(Andersen, Andersen, et al., 2008)  2) Saeterbakken et al., (2017)(Saeterbakken et al., 2017a) | RCTs | Very serious ^a^ | Not serious ^b^ | Not serious | Serious ^b^ | None | 25 | 18 | 1) MD [95%CI]: **5.16 mm**  [-6.38, 16.7] | ⨁⨁◯◯ LOW | CRITICAL |
| **A5.2. Aerobic exercise vs. non-treatment**  **Pain intensity in general: mean after 10 weeks follow-up; assessed with VAS (0-100mm)** | | | | | | | | | | | |
| 1) Andersen (a) et al., (2008)(Andersen, Andersen, et al., 2008)  2) Saeterbakken et al., (2017)(Saeterbakken et al., 2017a) | RCTs | Very serious ^a^ | Not serious | Not serious | Serious ^b^ | None | 25 | 30 | 1) MD [95%CI]: 7.21 **mm** [-5.14, 19.57] | ⨁⨁◯◯ LOW | CRITICAL |
| **A5.3 Aerobic exercise vs. strength exercise**  **Pain intensity in general: mean after the end of the treatment; assessed with VAS (0-100mm)** | | | | | | | | | | | |
| 1) Andersen (a) et al., (2008)(Andersen, Andersen, et al., 2008)  2) Saeterbakken et al., (2017)(Saeterbakken et al., 2017a) | RCT | Very serious ^a^ | Serious ^b^ | Not serious | Serious ^c^ | None | 25 | 30 | MD [95%CI]: **-11.34 mm** [-21.60, -1.09] | ⨁◯◯◯ VERY LOW | CRITICAL |
| **A5.4 Aerobic exercise vs. strength exercise**  **Pain intensity in general: mean after 10 weeks follow-up; assessed with VAS (0-100mm)** | | | | | | | | | | | |
| 1) Andersen (a) et al., (2008)(Andersen, Andersen, et al., 2008)  2) Saeterbakken et al., (2017)(Saeterbakken et al., 2017a) | RCT | Very serious ^a^ | Serious ^b^ | not serious | Serious ^c^ | None | 25 | 30 | MD [95%CI]: -**2.93 mm**  [-13.86, 8.01] | ⨁◯◯◯ VERY LOW | CRITICAL |
| **A5.5 Aerobic exercise vs. education therapy**  **Pain intensity in general: mean after the end of the treatment; assessed with VAS (0-100mm)** | | | | | | | | | | | |
| 1) Korshoj et al., (2018)(Korshøj et al., 2018) | RCT | Serious ^a^ | NA | Not serious | Not serious | None | 38 | 44 | MD [95%CI]: -**0.40 mm**  [-1.32, 0.52] | ⨁⨁◯◯ LOW | CRITICAL |
| **A5.6 Aerobic exercise combined plus other therapies (i.e., (1) strength training and (2) acupuncture) vs. other active interventions (i.e., (1) strength training and (2) acupuncture)**  **Pain intensity in general: mean after the end of the treatment; assessed with VAS (0-100mm)** | | | | | | | | | | | |
| 1) Daher et al. (2020) (Daher et al., 2020)  2) Eftekharsadat et al., (2018)(Bina Eftekharsadat et al., 2018) | RCT | Serious ^a^ | Not serious | Not serious | Serious ^b^ | None | 93 | 92 | 1) MD [95%CI]: **7.71 mm** [1.07, 14.35] | ⨁⨁◯◯ LOW | IMPORTANT |

MD: Mean difference; CI: Confidence interval; RCT: Randomized controlled trial; NA: Not applicable; VAS: Visual Analog Scale

| **Reasons to downgrade** | |
| --- | --- |
| **A5.1 and A5.2 Aerobic exercise vs. non-treatment**  **a.** The studies were rated with a high risk of bias (both studies presented problems with deviations from the intended intervention, missing data, and measurement of the outcome).  **b**. Although they presented the same direction of the results, the width of the confidence intervals for both studies is large. | **A5.5 Aerobic exercise vs. education therapy**  **a**. The study presents a high risk of bias. The study presented risks related to missing outcome data and measurement of the outcome. |
| **A5.3 and A5.4. Aerobic exercise vs. strength exercise**  **a.** The studies were rated with a high risk of bias (both studies presented problems with deviations from the intended intervention, missing data, and measurement of the outcome).  **b**. The direction of the results is different. Andersen et al (a) presented favored results for strength training while Saeterbakken et al. did not find any difference between groups, also they presented a moderate heterogeneity (I^2^ = 40%).  **c.** (Andersen, Andersen, et al., 2008; Saeterbakken et al., 2017a) Although they presented the same direction of the results, the width of the confidence interval for both studies is large. | **A5.6 Aerobic exercise plus other therapies vs. other active interventions**  **a**. Both studies had a high risk of bias. Daher et al., (Daher et al., 2020) presented problems with missing data, while Eftekharsadat et al. (Bina Eftekharsadat et al., 2018) with deviations from the intended intervention, missing outcome data, selection of reported results, and measurement of the outcome).  **b**. Both studies crossed the null. The sample size was 185. It ranged from a negative to a positive effect. The width of the confidence intervals is large |

## Appendix 6 – Secondary outcomes results

This section provides the results for the secondary outcomes of this study, which were summarized and ordered according to the structure of the IMMPACT. (Turk et al., 2003) Due to the diversity of the selected studies and outcomes investigated, only a few treatment effect estimates were pooled. Therefore, only a qualitative and a narrative synthesis of the results was given for most of the comparisons. The MCID for PPT is reported as > 1.10 kg/cm^2^. (Lourenço et al., 2016; Pool et al., 2007). The remaining outcomes were assessed based on effect size interpretation guidelines for rehabilitation treatment effects. (Kinney et al., 2020)

### **Tenderness (measured with Pain Pressure Threshold - PPT)**

***Aerobic exercise vs. control group (Figure A6.1.1)***

Kocur et al. (2017) evaluated tenderness using an algometer and compared the effectiveness of **Nordic Walking** *(low to moderate intensity, 60 min, 3x/week, for 12 weeks)* vs. the **control group** *(not to change their movement routines and habits).* They examined six muscles using the PPT. Since neck pain was the condition of interest for this systematic review, only the values for the trapezius muscle were of interest. Following the analysis of the authors using the Whitney-Base Test, the p-value (> 0.05) was not significant for the PPT for the trapezius muscle. However, using a simple t-test with Revman, there was a statistically significant difference between groups favoring aerobic exercise (MD [95%CI]: 0.60 kg/cm² [0.06, 1.14], Figure A6.1. comparison A6.1.1). (Kocur et al., 2017)

***Aerobic exercise plus acupuncture vs. acupuncture alone (Figure A6.1.2)***

B. Eftekharsadat et al. (2018) also evaluated PPT for two trigger point locations per patient, but they did not provide information about the exact location or which muscles were used, respectively. These values showed no statistical significance between the **aerobic exercise group plus acupuncture** *(treadmill/ bicycle, high intensity, 50 min, 3x/week, ten training sessions)* vs. **acupuncture alone** *(24 needles, both with additional stretching, 30 min, 3x/week, ten times*) (MD [95%CI]: 0.01 kg/cm² [-0.28, 0.30], Figure A6.1 comparison A6.1.2). (B. Eftekharsadat et al., 2018)

When measuring tenderness at two trigger point locations at one-month follow-up, the **aerobic exercise group** *(treadmill/ bicycle, high intensity, 50 min, 3x/week, ten training sessions)* from Eftekharsadat et al., became worse than the group that received **acupuncture alone** *(24 needles, both with additional stretching, 30 minutes, 3x/week, ten times)*; although the difference in PPTs between groups was not statistically significant (MD [95%CI]: -0.10 kg/cm² [-0.43, 0.23], Figure A6.1 comparison A6.1.3). (B. Eftekharsadat et al., 2018)


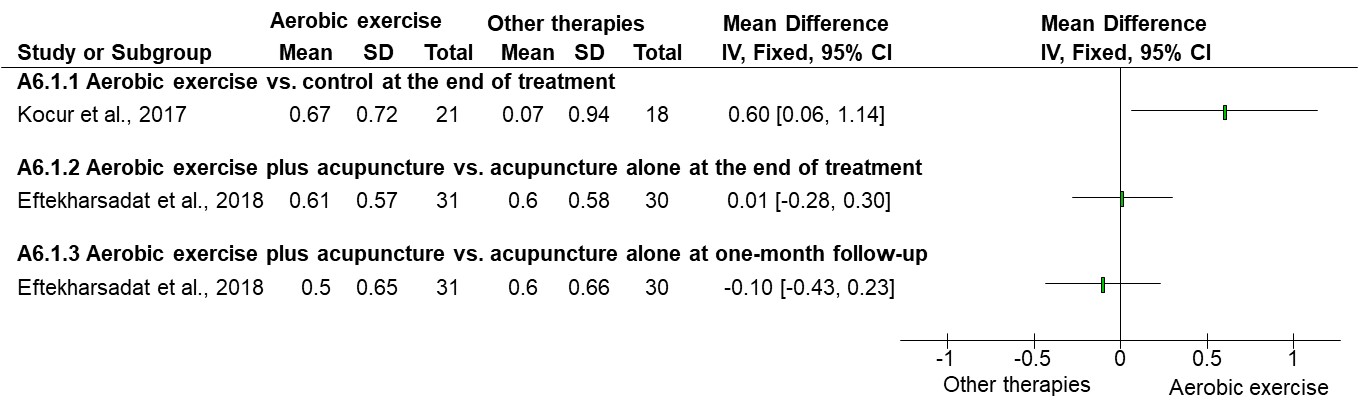


**Figure A6.1. Tenderness comparing aerobic exercise (AE) training vs. other therapies at the end of treatment and one-month follow-up**

All analyses are described as mean differences in kg/cm2 with fixed effects. The tenderness was measured using a pain pressure threshold (PPT) and an algometer. The effect sizes were calculated using the mean change before and after the treatments. Higher numbers mean greater tenderness improvement between before and after treatment.

#### Aerobic capacity (VO₂_max._, measured with Åstrand’s standardized method)

***Aerobic exercise vs. strength exercise (Figure A6.2.1)***

Andersen, Kjaer, et al. (2008) found no significant difference on VO₂_max_ between **aerobic exercise** when compared with the **strength exercise** group *(five dumbbell exercises specifically for the shoulder/neck muscles, 20 min, 3x/ week, ten weeks)* (MD [95%CI]: 3.00 ml/O₂/minute⁻¹ kg⁻¹ [-3.17, 9.17], Figure A6.2.1).(Andersen, Kjaer, et al., 2008) However, the effect size was considered moderate, according to Kinney et al.

***Aerobic exercise vs. education (Figure A6.2.2)***

Similarly, Andersen, Kjaer, et al. (2008) compared AE with the **education therapy** group *(health-promoting activities, 60 min, 1x/week, ten weeks),* and found no significant difference on VO₂_max_ between groups (MD [95%CI]: 3.00 ml/O₂/minute⁻¹kg⁻¹ [-4.76, 10.76] Figure A6.2.2).(Andersen, Kjaer, et al., 2008) However, the effect size was considered moderate, according to Kinney et al.,

**
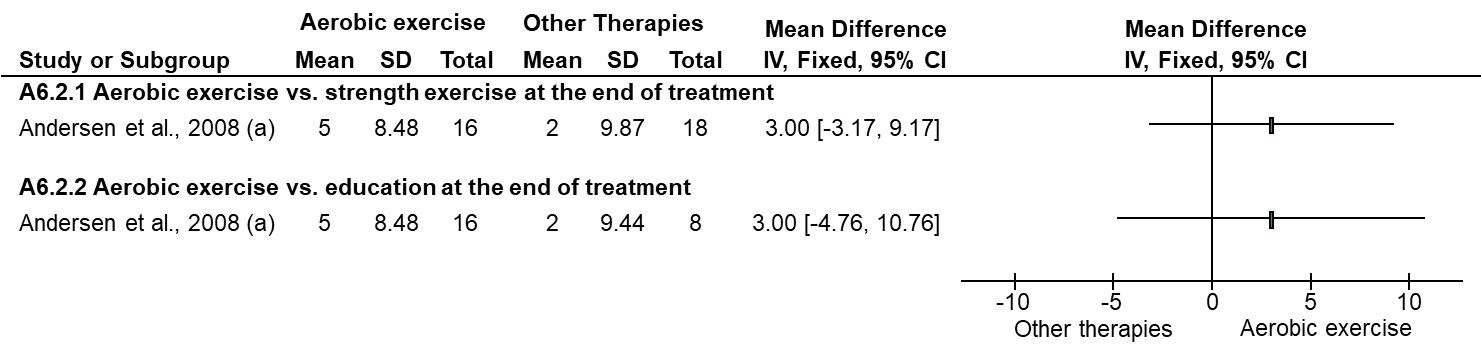
**

**Figure A6.2. Aerobic capacity comparing aerobic exercise (AE) training vs. other therapies (control) at the end of treatment**

All analyses are described as mean differences in VO₂max with fixed effects. The aerobic capacity was measured using a Åstrand’s standardized method. The effect sizes were calculated using the mean change before and after the treatments. Higher numbers mean greater aerobic capacity improvement between before and after treatment.

#### Maximal voluntary contraction (MVC)

#### Force cell measurement

***Aerobic exercise vs. strength exercise vs. no treatment***

Saeterbakken et al. (2017b) examined MVC isometric muscle strength during shoulder elevation and abduction for three groups. One group performed **aerobic exercise** *(Nordic walking, moderate intensity, 30 min, 2x/week, 10 weeks),* and one group performed **strength exercise** *(five exercises for neck and shoulder muscles with elastic bands (ropes), 30 min, 2x/week, 10 weeks)* and the control group received no intervention. The authors did not provide further values, just that there was no difference between groups (F=1.640, P=0.184). (Saeterbakken et al., 2017b)

#### Handheld dynamometer measurement

***Aerobic exercise vs. education or vs. strength exercise (Figure A6.3)***

Andersen, Kjaer, et al. (2008) compared **aerobic exercise** *(leg bicycling, moderate intensity, 20 min, 3x/week, ten weeks)* with **strength exercise** *(five dumbbell exercises specifically for the shoulder and neck muscles, 20 min, 3x/week, ten weeks)* and with an **education therapy** group *(health-promoting activities, 60 min, 1x/week, ten weeks*). The results showed that isometric muscle strength increased significantly in the strength group during shoulder elevation (right side: 30%, left side: 29%) and shoulder abduction (right side: 28%, left side: 29%). (Andersen, Kjaer, et al., 2008)

Figures A6.3 and A6.4 provide the between-group comparisons of maximal muscle strength values measured in Newtons for shoulder elevation and abduction. Even though aerobic exercise demonstrated lower values of muscle strength for all comparisons, there was only a significant difference between aerobic exercise and strength exercise for shoulder elevation on the left side (MD [95%CI]: -17 Newtons [-31.36, -2.64] Figure A6.3 comparison A6.3.2). This difference was considered clinically relevant according to interpretation guidelines, reaching a larger effect size. (Kinney et al., 2020)


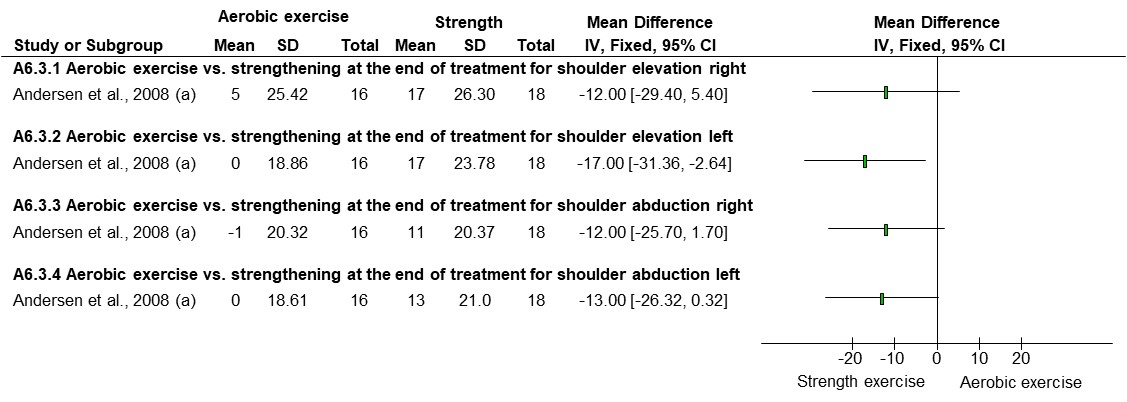


**Figure A6.3. Maximal voluntary contraction (MVC) measured with handheld dynamometer comparing aerobic exercise (AE) training vs. strength at the end of treatment**

All analyses are described as mean differences in Newtons with fixed effects. The MVC was measured using a force cell. The effect sizes were calculated using the mean change before and after the treatments. Higher numbers mean greater force improvement between before and after treatment.

**
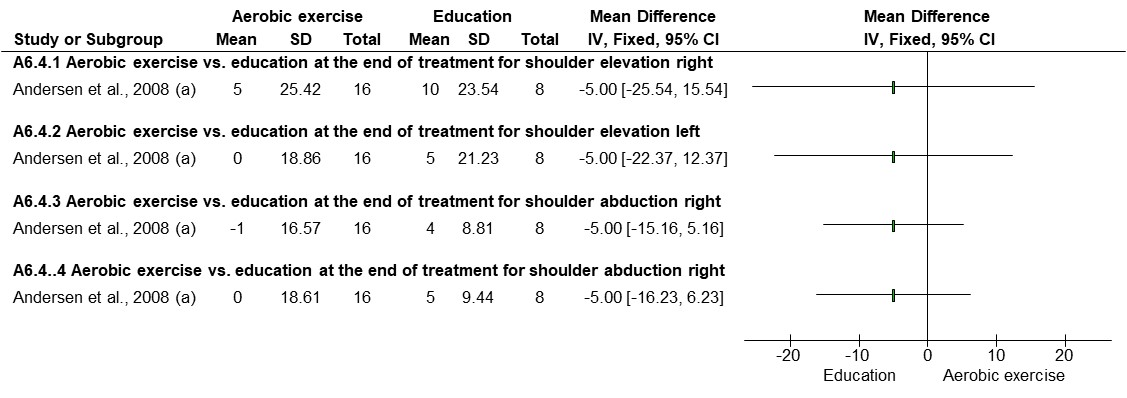
**

**Figure A6.4. Maximal voluntary contraction (MVC) measured with handheld dynamometer comparing aerobic exercise (AE) training vs. education therapy at the end of treatment**

All analyses are described as mean differences in Newtons with fixed effects. The MVC was measured using a force cell. The effect sizes were calculated using the mean change before and after the treatments. Higher numbers mean greater force improvement between before and after treatment.

#### Neck range of motion (ROM) and endurance (assessed during clinical examination)

***Aerobic exercise plus strength exercise vs. strength exercise alone (Figures A6.5 and A6.6)***

One study Daher et al. (2020) evaluated the range of motion and neck flexor muscle endurance, which were assessed during a clinical examination. The following Figure A6.5 provides an overview of the between-group differences *(****combined therapy*** *(aerobic exercise plus strengthening exercise) vs.* ***strengthening exercise alone*** *as described previously)* of range of motion for selected movements of the neck. (Daher et al., 2020) The authors reported that the combined therapy group showed better improvement in the ROM of neck rotation (p = 0.007, η2 = 0.063, 95%(CI) = 0.006, 0.162). (Daher et al., 2020) See Figure |A6.5 for detailed results regarding ROM.

For neck flexor muscle endurance, the combined group (aerobic exercise plus strength training) was statistically significantly superior to the strengthening exercise group (MD [95%CI]: 3.37 seconds [1.25, 5.49], Figure A6.6).


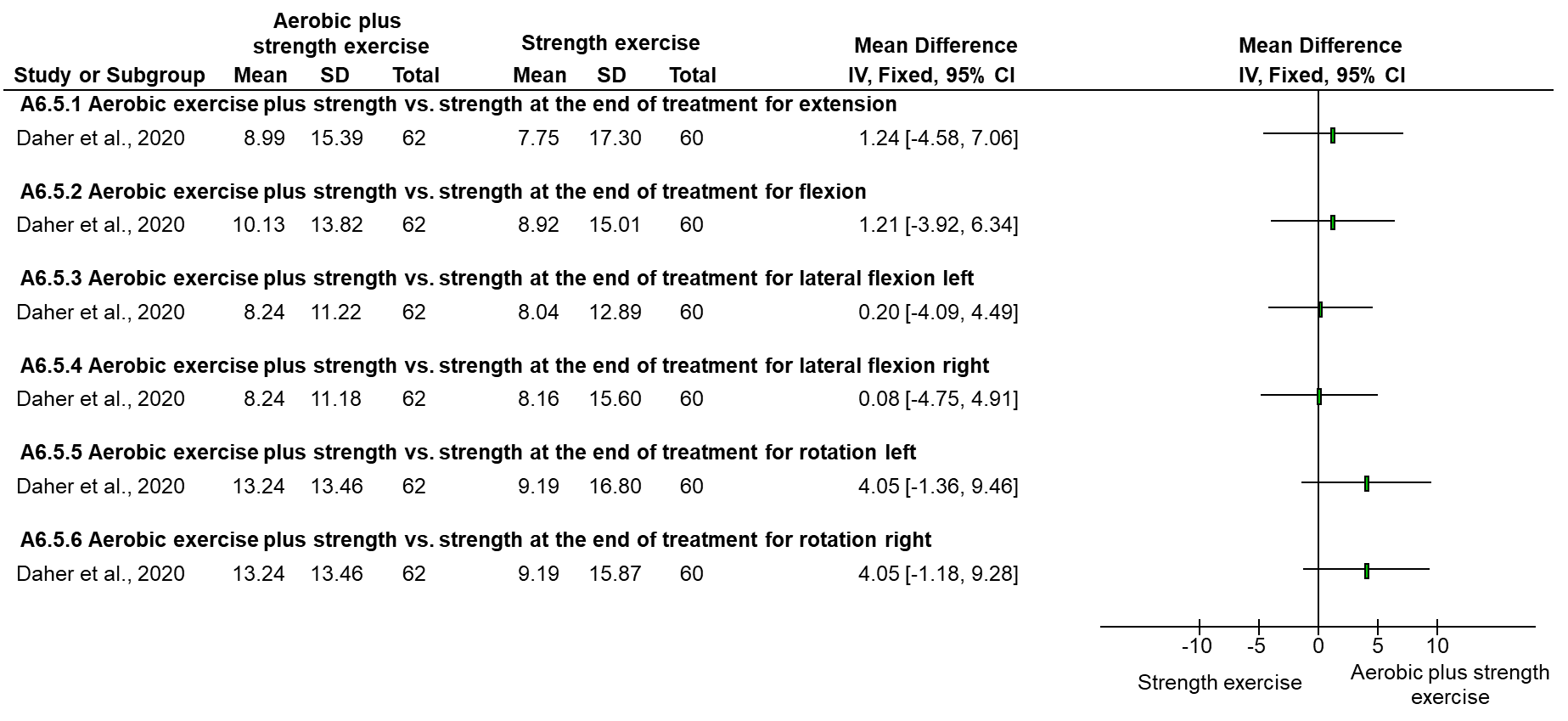


**Figure A6.5. Range of motion (ROM) comparing aerobic exercise (AE) training vs. other therapies at the end of treatment**

All analyses are described as mean differences in degrees with fixed effects. The effect sizes were calculated using the mean change before and after the treatments. Higher numbers mean greater range of motion improvement between before and after treatment.


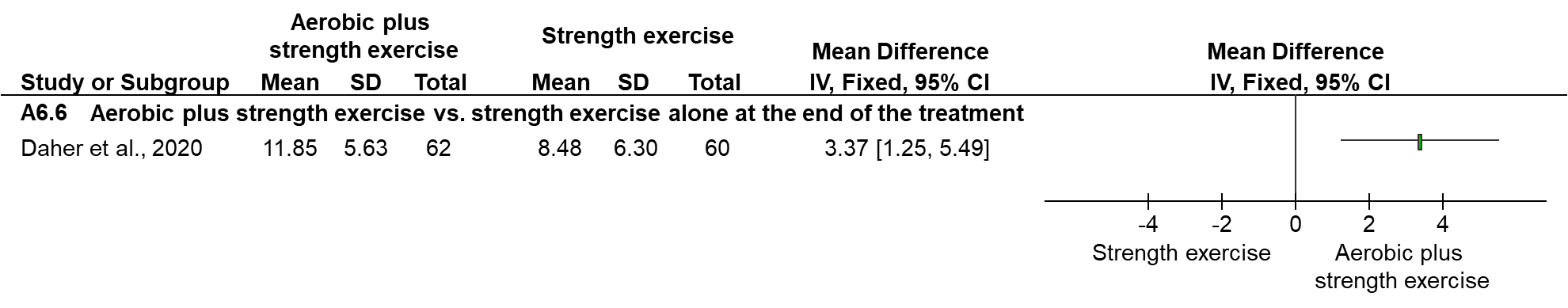


**Figure A6.6. Neck flexor endurance comparing aerobic exercise (AE) training vs. strength exercise at the end of treatment**

All analyses are described as mean differences in points with fixed effects. The neck flexor endurance was measured using the deep neck flexor muscle endurance test. The effect sizes were calculated using the mean change before and after the treatments. Higher numbers mean greater endurance improvement between before and after treatment.

#### Quality of life (measured with SF-36)

***Aerobic exercise plus acupuncture vs. acupuncture alone at the end of treatment (Figure A6.7)***

B. Eftekharsadat et al. (2018) evaluated quality of life using the SF-36 questionnaire and found no statistically significant difference between groups *(***combined therapy** *(aerobic plus acupuncture) vs.* **acupuncture alone** *as described previously)* neither at end of treatment (MD [95%CI]: 5.76 points [-16.55, 28.07]) nor at one month follow-up (MD [95%CI]: 5.33 points [-3.43, 14.09]) , Figure A6.7 comparisons A6.7.1 and A6.7.2). (B. Eftekharsadat et al., 2018)


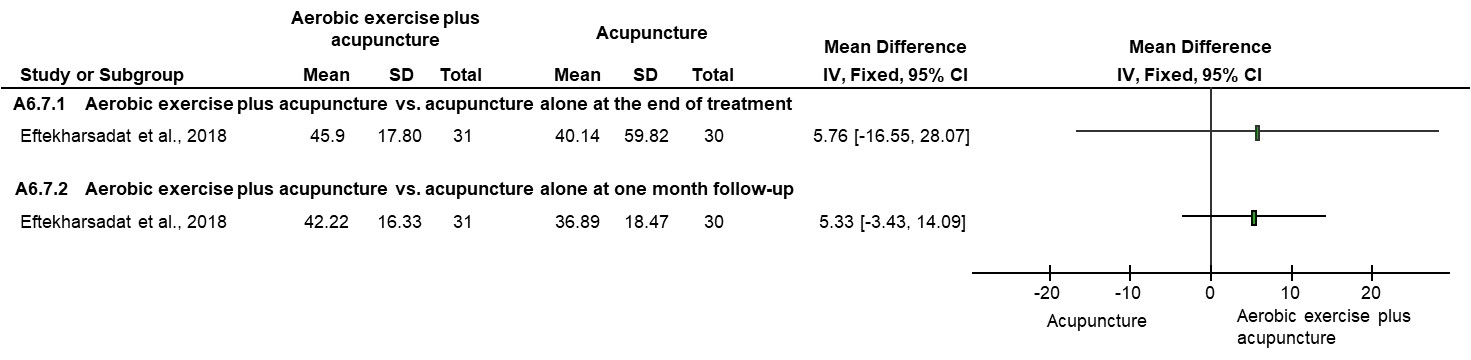


**Figure A6.7. Quality of life comparing aerobic exercise (AE) training plus acupuncture vs. acupuncture alone at the end of treatment and one-month follow-up**

All analyses are described as mean differences in points with fixed effects. The quality of life was measured using the SF-36 questionnaire. The effect sizes were calculated using the mean change before and after the treatments. Higher numbers mean greater quality of life improvement between before and after treatment.

#### Emotional functioning (measured with FABQ)

***Aerobic plus strength exercise vs. strength exercise alone (Figure A6.8)***

Daher et al., Daher et al. (2020) evaluated fear avoidance using the FABQ and found statistically significant differences between groups *(***combined therapy** *(aerobic exercise plus strength exercise) vs.* **strength exercise alone***; as described previously)* **at the end of the treatment** (MD [95%CI]: 5.97 points [1.18, 10.76] Figure A6.8, comparison A6.8.1). Furthermore, the authors differentiated between fear avoidance **during physical activity** (MD [95%CI]: 2.45 points [0.28, 4.62] Figure A6.8, comparison A6.8.2) and **during work** (MD [95%CI]: 3.53 points [-0.09, 7.15] Figure A6.8, comparison A6.8.3). (Daher et al., 2020) Based on the classification from Kinney et al., all values for this outcome were not only statistically significant but also of moderate clinical relevance, with the aerobic exercise group reaching moderate effect sizes at the end of the treatment during physical activity and **work**.


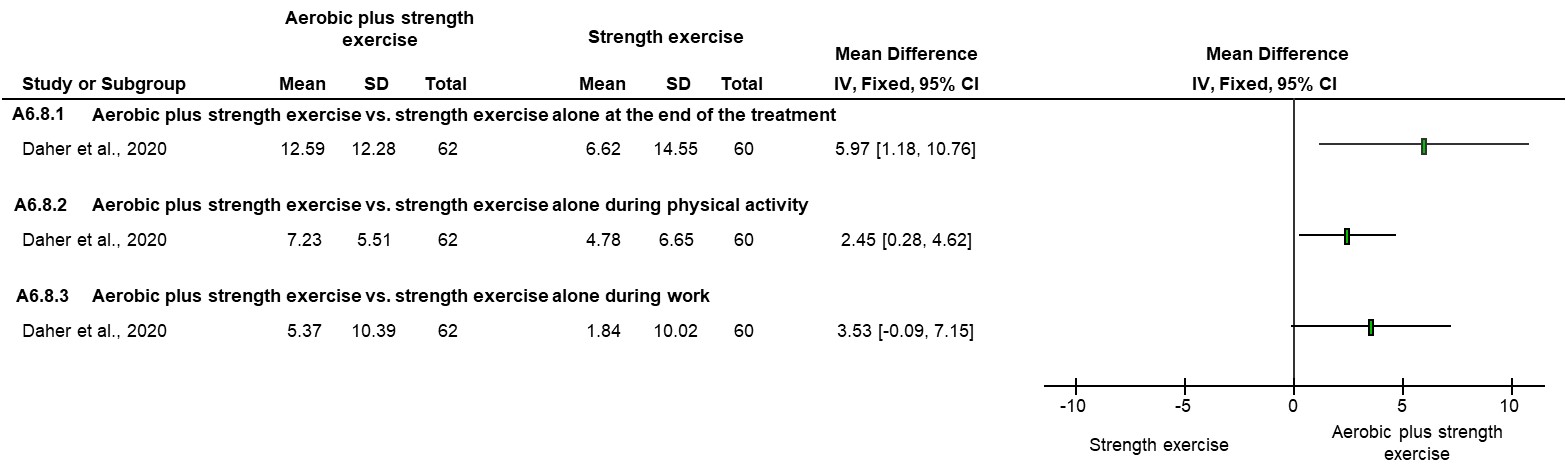


**Figure A6.8. Emotional functioning measured with the FABQ comparing aerobic exercise (AE) plus strength training vs. strength training alone and at the end of treatment**

All analyses are described as mean differences in points with fixed effects. The emotional function was measured using the Fear-Avoidance Beliefs Questionnaire (FABQ) questionnaire. The effect sizes were calculated using the mean change before and after the treatments. Higher numbers mean greater emotional function improvement between before and after treatment.

#### Participant ratings of global improvement (measured with 15-Point Global Rating of Change Scale - GRCS).

***Aerobic plus strength exercise vs. strength exercise alone at the end of treatment (Figure A6.9)***

Daher et al. (2020) evaluated participant ratings of global improvement and found non-significant differences between the groups; *(***combined therapy** *(aerobic exercise plus strength exercise) vs.* **strength exercise alone***; as described previously),* although the combined group had better scores than the group that received strength exercise alone (MD [95%CI]: 10 points [-9.30, 29.30] Figure A6.9, comparison A6.9.1). (Daher et al., 2020) However, the effect size for this comparison was small***Aerobic plus strength exercise vs. strength exercise alone at three-month follow-up (Figure A6.9)***

At a three-month follow-up, Daher et al. *(Daher et al., 2020)* found an increased difference between the groups [**combined therapy** (*aerobic exercise plus strength exercise*) *vs****.* strength exercise alone** (*as described previously*)] (Daher et al., 2020). However, this difference was still not statistically significant (MD [95%CI]: 20 points [-0.37, 40.37] Figure A6.9 comparison A6.9.2)*.* The effect size of this comparison was moderate according to the guidelines of Kinney et al. (Kinney et al., 2020)

***Aerobic plus strength exercise vs. strength exercise alone at six months follow-up (Figure A6.9)***

At six months follow-up, the difference between groups [**combined therapy** *(aerobic exercise plus strength exercise) vs****.* strength exercise alone** *(as described previously)*] increased further (Daher et al., 2020). This difference reached significance and favored the combined therapy (MD [95%CI]: 24 points [2.24, 45.76] Figure A6.9 comparison A6.9.3)*.* Furthermore, the effect size was considered moderate according to Kinney et al. (Kinney et al., 2020)


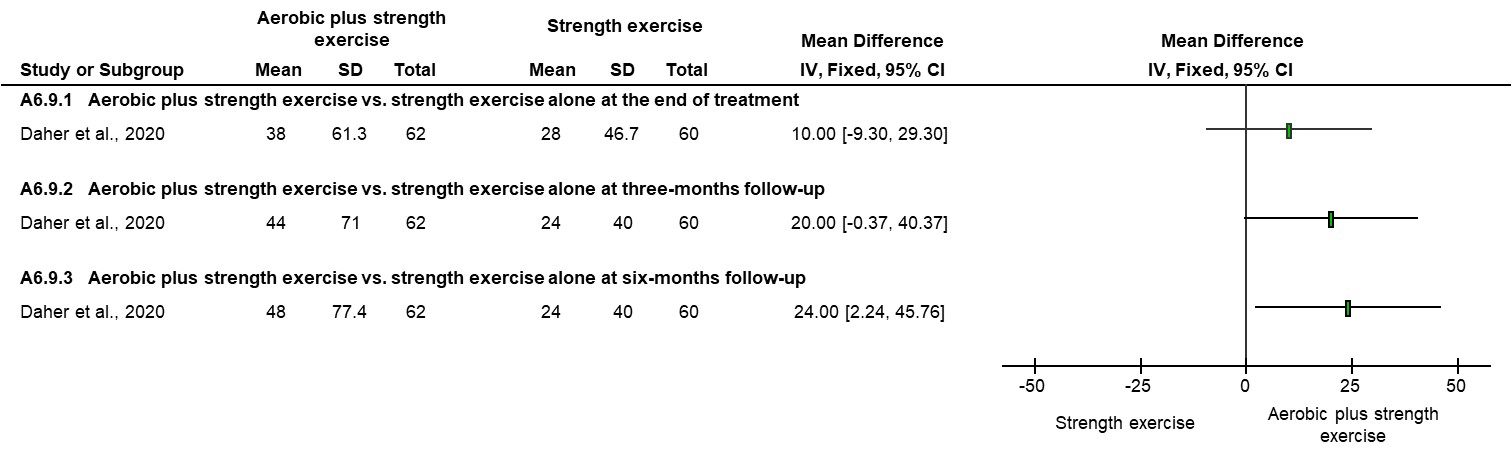


**Figure A6.9. Global improvement comparing aerobic exercise (AE) plus strength training vs. strength training alone and at the end of treatment and three- and six-months follow-up**

All analyses are described as mean differences in points with fixed effects. The global improvement was measured using the 15-points Global Rating of Change Scale (GRCS). The effect sizes were calculated using the mean change before and after the treatments. Higher numbers mean greater global improvement between before and after treatment.

**References**

Andersen, L. L., Andersen, C. H., Zebis, M. K., Nielsen, P. K., Søgaard, K., & Sjøgaard, G. (2008). Effect of physical training on function of chronically painful muscles: a randomized controlled trial. *J Appl Physiol (1985)*, *105*(6), 1796-1801. <https://doi.org/10.1152/japplphysiol.91057.2008>

Andersen, L. L., Kjaer, M., Sogaard, K., Hansen, L., Kryger, A. I., & Sjogaard, G. (2008). Effect of two contrasting types of physical exercise on chronic neck muscle pain. *Arthritis & Rheumatism-Arthritis Care & Research*, *59*(1), 84-91. <https://doi.org/10.1002/art.23256>

Daher, A., Carel, R. S., Tzipi, K., Esther, H., & Dar, G. (2020). The effectiveness of an aerobic exercise training on patients with neck pain during a short- and long-term follow-up: a prospective double-blind randomized controlled trial. *Clin Rehabil*, *34*(5), 617-629. <https://doi.org/10.1177/0269215520912000>

Eftekharsadat, B., Porjafar, E., Eslamian, F., Shakouri, S. K., Fadavi, H. R., Raeissadat, S. A., & Babaei-Ghazani, A. (2018). Combination of exercise and acupuncture versus acupuncture alone for treatment of myofascial pain syndrome: a randomized clinical trial. *Journal of Acupuncture and Meridian Studies*, *11*(5), 315-322.

Eftekharsadat, B., Porjafar, E., Eslamian, F., Shakouri, S. K., Fadavi, H. R., Raeissadat, S. A., & Babaei-Ghazani, A. (2018). Combination of Exercise and Acupuncture Versus Acupuncture Alone for Treatment of Myofascial Pain Syndrome: A Randomized Clinical Trial. *J Acupunct Meridian Stud*, *11*(5), 315-322. <https://doi.org/10.1016/j.jams.2018.04.006>

Kinney, A. R., Eakman, A. M., & Graham, J. E. (2020). Novel Effect Size Interpretation Guidelines and an Evaluation of Statistical Power in Rehabilitation Research. *Arch Phys Med Rehabil*, *101*(12), 2219-2226. <https://doi.org/10.1016/j.apmr.2020.02.017>

Kocur, P., Pospieszna, B., Choszczewski, D., Michalowski, L., Wiernicka, M., & Lewandowski, J. (2017). The effects of Nordic Walking training on selected upper-body muscle groups in female-office workers: A randomized trial. *Work*, *56*(2), 277-283. <https://doi.org/10.3233/wor-172497>

Korshøj, M., Birk Jørgensen, M., Lidegaard, M., Mortensen, O. S., Krustrup, P., Holtermann, A., & Søgaard, K. (2018). Decrease in musculoskeletal pain after 4 and 12 months of an aerobic exercise intervention: a worksite RCT among cleaners. *Scand J Public Health*, *46*(8), 846-853. <https://doi.org/10.1177/1403494817717833>

Lourenço, A. S., Lameiras, C., & Silva, A. G. (2016). Neck Flexor and Extensor Muscle Endurance in Subclinical Neck Pain: Intrarater Reliability, Standard Error of Measurement, Minimal Detectable Change, and Comparison With Asymptomatic Participants in a University Student Population. *J Manipulative Physiol Ther*, *39*(6), 427-433. <https://doi.org/10.1016/j.jmpt.2016.05.005>

Pool, J. J., Ostelo, R. W., Hoving, J. L., Bouter, L. M., & de Vet, H. C. (2007). Minimal clinically important change of the Neck Disability Index and the Numerical Rating Scale for patients with neck pain. *Spine (Phila Pa 1976)*, *32*(26), 3047-3051. <https://doi.org/10.1097/BRS.0b013e31815cf75b>

Saeterbakken, A. H., Nordengen, S., Andersen, V., & Fimland, M. S. (2017a). Nordic walking and specific strength training for neck- and shoulder pain in office workers: a pilot-study. *Eur J Phys Rehabil Med*, *53*(6), 928-935. <https://doi.org/10.23736/s1973-9087.17.04623-8>

Saeterbakken, A. H., Nordengen, S., Andersen, V., & Fimland, M. S. (2017b). Nordic walking and specific strength training for neck- and shoulder pain in office workers: A pilot-study [Article]. *European Journal of Physical and Rehabilitation Medicine*, *53*(6), 928-935. <https://doi.org/10.23736/S1973-9087.17.04623-8>

Turk, D. C., Dworkin, R. H., Allen, R. R., Bellamy, N., Brandenburg, N., Carr, D. B., Cleeland, C., Dionne, R., Farrar, J. T., Galer, B. S., Hewitt, D. J., Jadad, A. R., Katz, N. P., Kramer, L. D., Manning, D. C., McCormick, C. G., McDermott, M. P., McGrath, P., Quessy, S., . . . Witter, J. (2003). Core outcome domains for chronic pain clinical trials: IMMPACT recommendations. *Pain*, *106*(3), 337-345. <https://doi.org/10.1016/j.pain.2003.08.001>
